# Multi-omics links microbial dysbiosis, systemic inflammation, and metabolomic disruptions to SNAE risk in treated HIV

**DOI:** 10.1172/jci.insight.205379

**Published:** 2026-07-22

**Authors:** Christopher M. Basting, Jodi Anderson, Kevin Escandón, Garritt Wieking, Candace Guerrero, Jarrett Reichel, Ross T. Cromarty, Erik Swanson, Ty Schroeder, Elaina Creagan, Maura Barrett, Fernanda Torres-Ruiz, Maribel Soto-Nava, Lady Carvajal-Ruiz, Karla Krystel Ordaz-Candelario, Olivia Briceño, Nicholas Funderburg, Melanie Graham, Peter Hunt, Santiago Avila-Rios, Gonzalo Salgado Montes de Oca, Timothy W. Schacker, Nichole R. Klatt

**Affiliations:** 1 Division of Surgical Outcomes and Precision Medicine Research, Department of Surgery,; 2 Division of Infectious Diseases and International Medicine, Department of Medicine, and; 3 Masonic Cancer Center, University of Minnesota, Minneapolis, Minnesota, USA.; 4 Instituto Nacional de Enfermedades Respiratorias Ismael Cosío Villegas, Centro de Investigación en Enfermedades Infecciosas, Mexico City, Mexico.; 5 School of Health and Rehabilitation Sciences, Ohio State University, Columbus, Ohio, USA.; 6 Pre-Clinical Research Center, Department of Surgery, University of Minnesota, Minneapolis, Minnesota, USA.; 7 Division of Experimental Medicine, University of California San Francisco. San Francisco, California, USA.

**Keywords:** AIDS/HIV, Inflammation, Microbiology, Cytokines, Metabolomics, Microbiome

## Abstract

Serious non-AIDS events (SNAEs), including non-AIDS malignancies, cardiovascular disease, and hepatic complications, remain major causes of mortality in treated HIV infection. These outcomes are driven by persistent immune activation, systemic inflammation, and metabolic dysfunction despite effective viral suppression with antiretroviral therapy (ART). To investigate mechanisms underlying SNAE pathogenesis, we performed a cross-site multi-omic analysis integrating plasma proteins, plasma metabolites, and mucosal microbiomes in 82 ART-treated people with HIV (PWH) and 10 people without HIV from the United States and Mexico. Geography was the dominant source of variation, particularly across lipid classes. However, individuals at high risk for SNAEs, defined by low CD4^+^ T cell counts and low CD4/CD8 ratios, shared a consistent signature of systemic inflammation, mitochondrial dysfunction, and microbial dysbiosis, including elevated plasma IL-6 and ω-oxidation products (adipic and suberic acids) and depletion of short-chain fatty acid–producing commensals in the gut mucosa, including *Akkermansia muciniphila*, *Bacteroides uniformis*, and *Ruminococcus*. *A*. *muciniphila* abundance correlated with lower IL-6 levels, fewer HIV RNA-producing cells in lymph nodes, and higher CD4/CD8 ratios. These findings identify a shared inflammatory and metabolic phenotype in PWH and implicate *A*. *muciniphila* as a potential microbiome-based target to mitigate immune activation and SNAE risk in treated HIV.

## Abstract 2

People with treated HIV still face serious health risks driven by inflammation. Those at highest risk share a pattern of gut bacterial loss and metabolic dysfunction, with one gut microbe showing particular protective potential.

## Introduction

Antiretroviral therapy (ART) has transformed HIV into a chronic condition with near-normal life expectancy, yet a gap remains largely due to serious non-AIDS events (SNAEs). These include non-AIDS malignancies, cardiovascular events (e.g., myocardial infarction and stroke), and hepatic disease, which remain major causes of morbidity and mortality in treated HIV infection despite ART (1, 2). The pathogenesis of SNAEs is multifactorial, attributed in part to chronic immune activation sustained by microbial translocation, coinfections, and persistent HIV reservoirs (3–7). This chronic immune activation drives systemic inflammation as well as immune dysfunction characterized by altered T cell counts, immune cell senescence, and metabolic dysfunction, further contributing to SNAE pathogenesis (8, 9). T cell criteria including low CD4^+^ T cell counts and a low CD4/CD8 T cell ratio are particularly well-established correlates of immune activation and dysfunction, often used as endpoints in interventional studies and predictors of SNAE risk (4, 10).

Current strategies to prevent SNAEs focus on early ART initiation, treatment of coinfections such as CMV and Epstein-Barr virus, and cardioprotective lifestyle changes (1), though these do not fully address the sources of immune activation and dysfunction that underlie SNAE pathogenesis. Interventional studies targeting these sources, such as fibrosis or microbial translocation, have had mixed results at improving correlates of SNAE risk (1, 11–14), highlighting the need for a better understanding of mechanisms driving SNAE pathogenesis. This is especially crucial for individuals at highest risk, such as those with less than 350 CD4^+^ T cells/μL after 2 years of ART, who are nearly 3 times more likely to have SNAE over the subsequent 5 years than individuals with greater than 350 CD4^+^ T cells/ μL, with similar associations observed for low CD4/CD8 ratios (10). Interventions that effectively reduce immune activation and restore immune homeostasis in these individuals could substantially lower SNAE incidence and improve long-term survival of people with HIV (PWH).

To address this knowledge gap, we conducted a cross-site, multi-omic analysis integrating plasma proteins (cytokines, microbial translocation, and gut barrier damage markers), the mucosal microbiome (ileum and rectum), and the plasma metabolome in 82 ART-treated PWH and 10 people without HIV (PWoH) enrolled from the United States and Mexico. Our objectives were 3-fold: (a) to characterize the multi-omic differences between PWH from the 2 geographic cohorts; (b) to identify geography-independent biomarkers of SNAE risk that may reveal shared biological mechanisms underlying SNAE pathogenesis; and (c) to highlight potential therapeutic interventions that could mitigate the drivers of SNAE pathogenesis in high-risk individuals.

## Results

### Differences in baseline characteristics.

Baseline characteristics for enrolled participants from the United States and Mexico are shown in Table 1. Compared with PWH from the United States, PWH from Mexico were older (*P* = 0.037) and had significantly lower CD4^+^ T cell counts (*P* = 0.006) and a lower nadir CD4^+^ T cell count (*P* = 0.003). When stratified by SNAE risk group (Table 2, with PWoH included as a reference), the high-risk group had significantly lower CD4^+^ T cell counts (*P* < 0.001), a lower CD4/CD8 ratio (*P* = 0.001), and lower nadir CD4^+^ T cell count (*P* = 0.002) compared with the low-risk group. There was no significant difference in country of enrollment (*P* = 0.6) or race (*P* = 0.8) between low- and high-risk groups. CMV IgG serostatus was assessed in all HIV-positive participants, of which all but 1 were seropositive (Supplemental Tables 1 and 2; supplemental material available online with this article; https://doi.org/10.1172/jci.insight.205379DS1). Summaries of ART regimens are provided with stratification by country (Supplemental Table 3) and SNAE risk group (Supplemental Table 4). Most enrolled participants (78%) were on an ART regimen consisting of 2 nucleoside/nucleotide reverse transcriptase inhibitors (NRTIs) and 1 integrase strand transfer inhibitor (INSTI). There was a significant difference in ART regimens between countries (*P* < 0.001), reflecting differences in national treatment guidelines. There was no difference in ART regimens between SNAE risk groups (*P* = 0.5). Additionally, clinical characteristics did not differ significantly between PWoH across countries (Supplemental Table 5).

### Multi-omic geographic differences in PWH on ART.

Geographic and environmental factors shape the microbiome, metabolome, and immune landscape, yet most HIV omic studies have focused on cohorts from high-income countries. The global heterogeneity of the gut microbiome in PWH, and its intersection with metabolic and immune phenotypes, remains poorly characterized.

**Figure 1:**
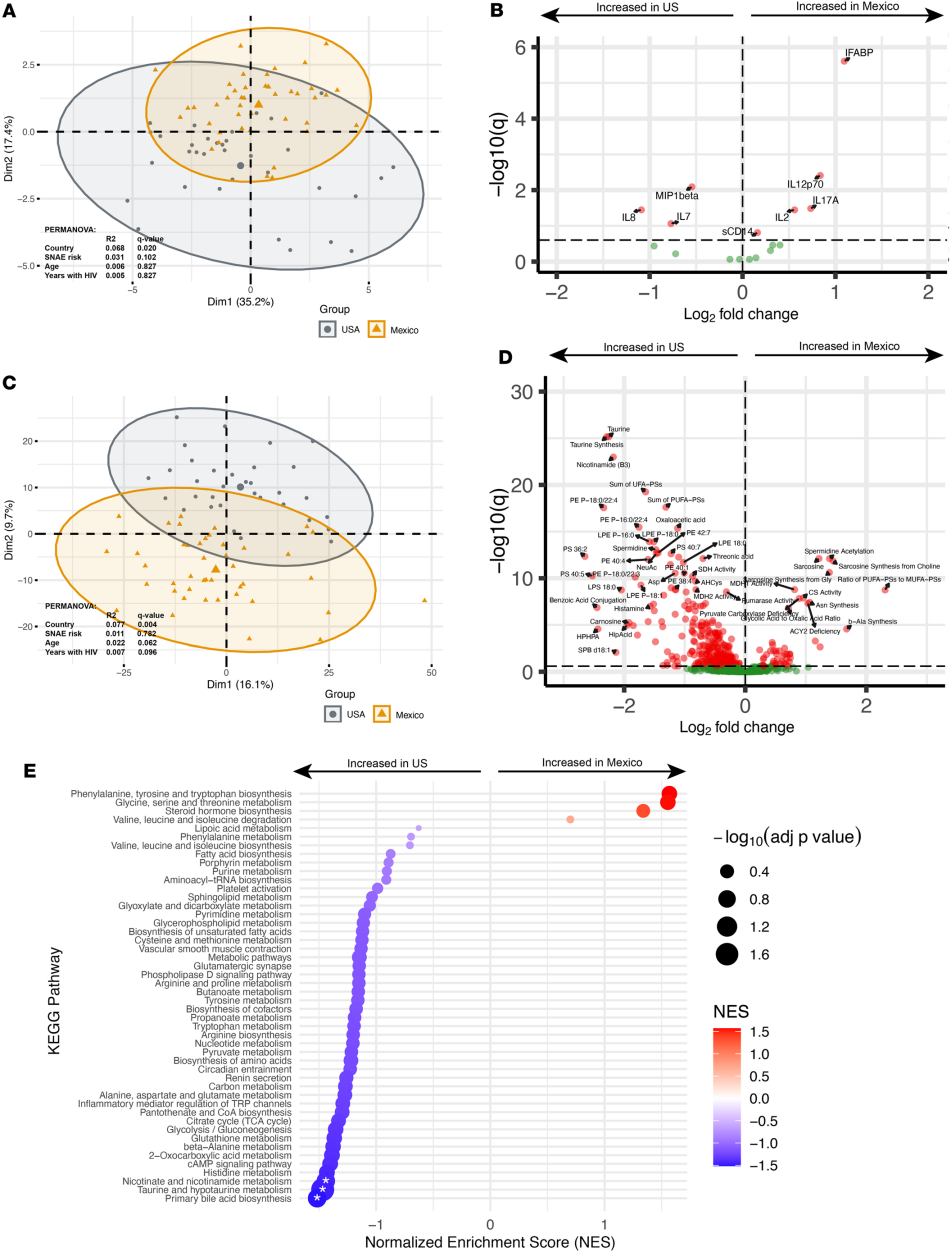
Differences in plasma proteins and metabolome between PWH enrolled in the United States and Mexico. (**A**) Principal component analysis of plasma protein concentrations, colored by country of enrollment and tested by PERMANOVA. (**B**) Volcano plot of cytokines and gut barrier integrity markers differentially associated with country of enrollment. (**C**) Principal component analysis of plasma metabolomic features, colored by country of enrollment and tested by PERMANOVA. (**D**) Volcano plot of metabolomic features differentially associated with country of enrollment. (**E**) GSEA of metabolomic data comparing KEGG pathway enrichment between PWH in the United States and Mexico; asterisks denote FDR-adjusted *q* ≤ 0.25.

We first looked at plasma concentrations of cytokines, markers of gut epithelial barrier dysfunction, and microbial translocation, including intestinal fatty acid binding protein (I-FABP), lipopolysaccharide binding protein (LBP), and soluble CD14 (sCD14), by principal component analysis (PCA). Country of enrollment was the largest and only significant effect on the overall composition of these biomarkers when tested by PERMANOVA (*R*^2^ = 0.068, *q* = 0.020, Figure 1A). Numerous plasma proteins were differentially abundant between participants in different countries (Figure 1B). Compared with PWH from the United States, those from Mexico had significantly higher concentrations of I-FABP (*P* < 0.001, *q* < 0.001) and sCD14 (*P* = 0.018, *q* = 0.1637). Cytokines involved in antigen presentation (15), mucosal function (16), and proliferation (17) were also elevated in patients from Mexico including IL-12p70 (*P* < 0.001, *q* = 0.0028), IL-17A (*P* = 0.002, *q* = 0.0341), and IL-2 (*P* = 0.003, *q* = 0.0341). PWH from the United States were characterized by increased concentrations of macrophage inflammatory protein 1β (MIP-1β) (*P* < 0.001, *q* = 0.0086), IL-7 (*P* = 0.009, *q* = 0.0916), and IL-8 (*P* = 0.003, *q* = 0.0341).

We next evaluated the plasma metabolome of PWH from the United States or Mexico using a comprehensive targeted liquid chromatography–tandem mass spectrometry panel of 1,006 metabolites, including 751 lipids, 255 small molecules, and 273 sums or ratios, for a total of 1,279 metabolomic features after filtering. Once again, PCA showed that country was the largest and only significant effect on the overall metabolomic profile composition (*R*^2^ = 0.077, *q* = 0.004, Figure 1C), followed by age. Differential analysis revealed 357 metabolomic features that were significantly different between PWH in the United States and Mexico at an adjusted *P* value threshold of 0.25 or less, and 236 at a threshold of 0.05 or less, most of which were elevated in PWH from the United States (Figure 1D and Supplemental Data 1). When looking at the classes of significantly different metabolites (*q* ≤ 0.25, Supplemental Figure 1), phospholipids were especially higher in PWH from the United States, including phosphatidylethanolamines (PEs), phosphatidylcholines, phosphatidylglycerols (PGs), and phosphatidylserines, which represented 39.6% of the significantly different metabolites. The lyso-forms of these lipid classes were also elevated in PWH from the United States including lyso-PEs and lysophosphatidylcholines. Sphingomyelins were also exclusively elevated in PWH from the United States. The primary metabolite class that was most represented by PWH in Mexico was acylcarnitines. When looking at individual metabolites, taurine, nicotinamide, oxaloacetic acid, spermidine, and acetylneuraminic acid were some of the top US-associated metabolites (*P* < 0.001 and *q* < 0.001 for all metabolites), and sarcosine (*P* < 0.001, *q* < 0.001), biotin (*P* < 0.001, *q* < 0.001), glycolic acid (*P* < 0.001, *q* = 0.001), octanoylcarnitine (*P* < 0.001, *q* = 0.012), and decanoylcarnitine (*P* < 0.001, *q* = 0.012) were some of the top Mexico-associated metabolites. Gene set enrichment analysis (GSEA) of the metabolite data showed that the Kyoto Encyclopedia of Genes and Genomes (KEGG) pathways for primary bile acid biosynthesis, taurine/hypotaurine metabolism, and nicotinate/nicotinamide metabolism were all enriched in PWH from the United States (*q* < 0.25, Figure 1E).

**Figure 2:**
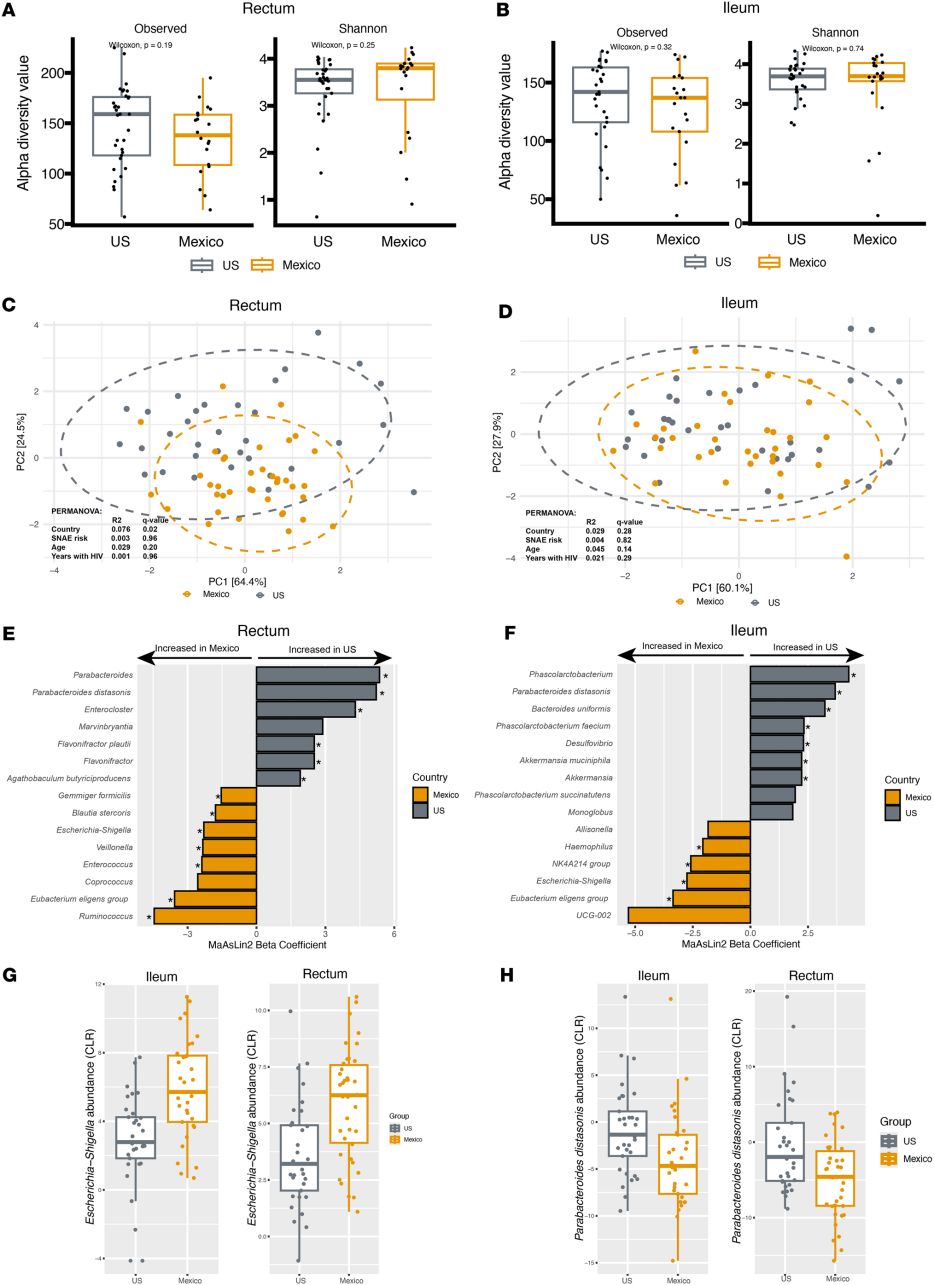
Differences in the ileal and rectal microbiomes of PWH enrolled from the United States and Mexico. Alpha diversity metrics for rectum (**A**) and ileum (**B**) biopsies between PWH from the United States and Mexico. Beta diversity of rectum (**C**) and ileum (**D**) biopsies, tested by PERMANOVA using the robust Aitchison distance of genus-level taxa. Differential abundance of genus- and species-level taxa between the United States and Mexico, tested by MaAsLin2 for rectum (**E**) and ileum (**F**) biopsies, showing the top 15 differentially abundant taxa; asterisks denote FDR-adjusted *q* ≤ 0.25. Representative box plots of *Escherichia-Shigella* (**G**) and *Parabacteroides distasonis* (**H**) in both the ileum and rectum.

At the phylum level, ileal and rectal biopsies were primarily composed of *Bacillota* (formerly *Firmicutes*) and *Bacteroidota* (Supplemental Figure 2A). We did not observe any difference in alpha diversity metrics for rectal or ileal biopsies between the United States and Mexico (Figure 2, A and B, respectively). Overall microbial composition of the rectal biopsies (Figure 2C), but not ileal biopsies (Figure 2D), was significantly associated with country of enrollment (PERMANOVA: *R*^2^ = 0.076, *q* = 0.02). Differential abundance analysis performed at the genus and species level showed increased abundances of *Ruminococcus, Eubacterium eligens group, Enterococcus, Veillonella, Escherichia-Shigella, Blautia stercoris,* and *Gemmiger formicilis* in the rectal biopsies of PWH from Mexico and increased abundances of *Agathobaculum butyriciproducens, Flavonifractor, Flavonifractor plautii, Enterocloster, Parabacteroides distasonis,* and *Parabacteroides* in PWH from the United States (*q* < 0.25, Figure 2E). When comparing the ileal biopsies, we observed increased abundance of *Eubacterium eligens group, Escherichia-Shigella*, *Oscillospiraceae* NK4A214 group, and *Haemophilus* in PWH from Mexico, whereas *Akkermansia*, *Akkermansia muciniphila*, *Desulfovibrio, Phascolarctobacterium faecium, Bacteroides uniformis, Parabacteroides distasonis*, and *Phascolarctobacterium* were all increased in PWH from the United States (*q* < 0.25, Figure 2F). Overall, some of the most striking differences between countries were the consistent increased abundance of *Escherichia-Shigella* (Figure 2G) and decreased abundance of *Parabacteroides distasonis* (Figure 2H) in PWH from Mexico versus the United States in both the ileal and rectal biopsies. We did not see any significant correlations between *Escherichia-Shigella* abundance in PWH from Mexico with mucosal cytokines or markers of gut barrier damage (Supplemental Figure 3).

We next examined whether these multi-omic differences between geographic locations were also present in PWoH. No significant differences in cytokine concentrations were observed between PWoH from the United States and Mexico, though this may reflect the limited sample size of the HIV-negative cohort given that several cytokines, including IL-17A, showed nonsignificant but concordant effect directions (Supplemental Figure 4A). For rectal and ileal biopsy taxa, none of the significant differences identified in PWoH overlapped with those seen in PWH, although a number of PWH-significant taxa again showed nonsignificant concordant effects in PWoH (e.g., ileal *Escherichia-Shigella* and *Bacteroides uniformis*; Supplemental Figure 4B). In contrast, 98 of the metabolites that differed significantly between PWH cohorts were also significantly different (*q* < 0.25) and directionally concordant in the PWoH comparison (Supplemental Figure 4C), suggesting that many of these metabolic differences are a geographic effect not unique to PWH.

### Systemic inflammation and metabolomic disruptions in PWH at high risk of SNAEs.

**Figure 3:**
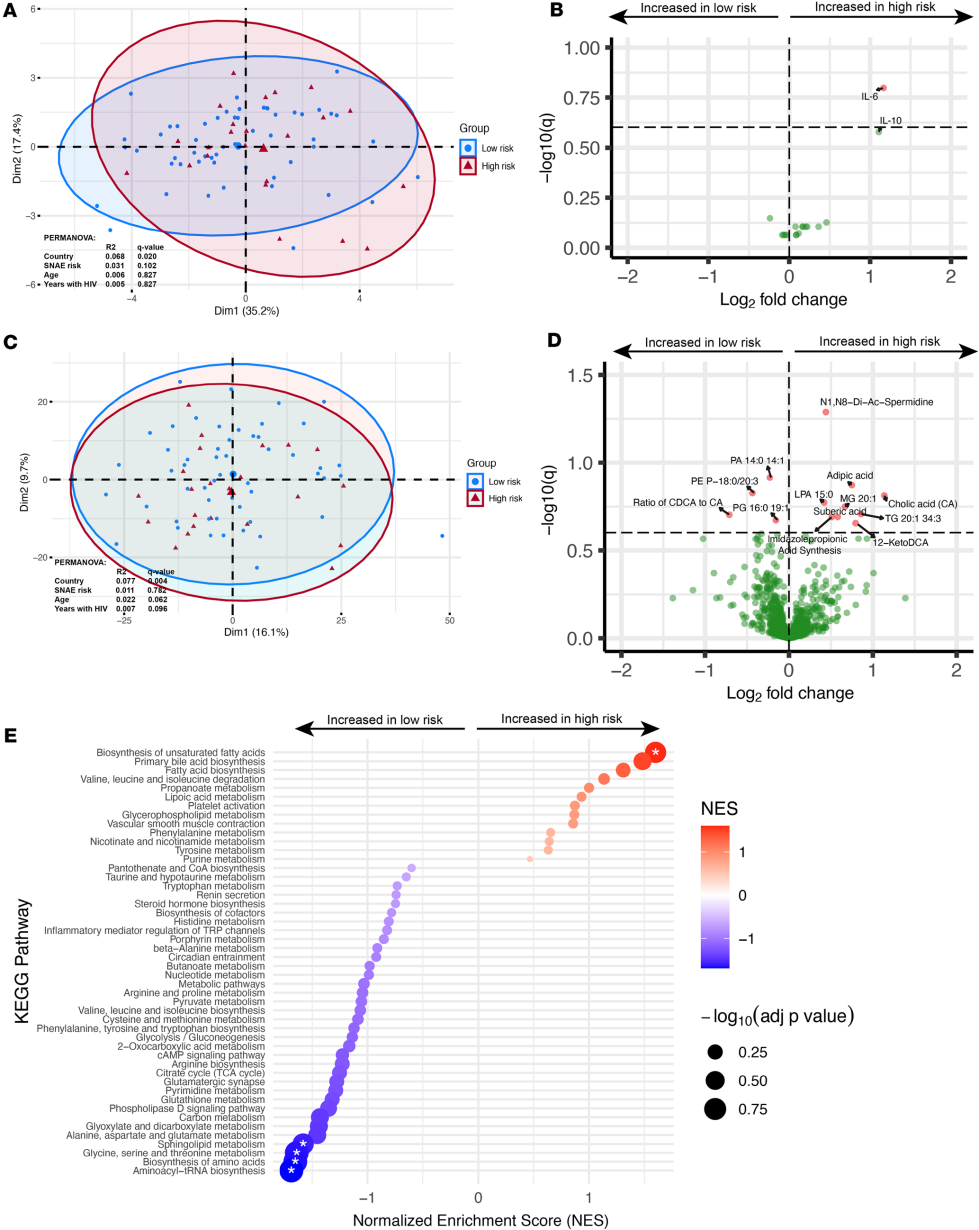
Differences in plasma proteins and metabolome between PWH at low or high risk of SNAEs. (**A**) Principal component analysis of plasma protein concentrations, colored by SNAE risk group, tested by PERMANOVA. (**B**) Volcano plot of cytokines and gut barrier integrity markers differentially associated with SNAE risk group. (**C**) Principal component analysis of plasma metabolomic features, colored by SNAE risk group and tested by PERMANOVA. (**D**) Volcano plot of metabolomic features differentially associated with SNAE risk group. (**E**) GSEA of metabolomic data comparing KEGG pathway enrichment between low and high SNAE risk groups; asterisks denote FDR-adjusted *q* ≤ 0.25.

Given the geographic differences between participants from the United States and Mexico, we next examined whether there were biomarkers distinguishing PWH at low versus high risk for SNAEs, defined by CD4^+^ T cell counts and the CD4/CD8 ratio, that were independent of geographic effects. Such biomarkers could provide important insights into the processes driving SNAE risk. We first compared concentrations of plasma cytokines and markers of gut epithelial dysfunction. In contrast to country of enrollment, SNAE risk group did not have a significant effect on the overall plasma protein profile when tested by PERMANOVA (*R*^2^ = 0.031, *q* = 0.102, Figure 3A). When looking at individual markers, IL-6 was the only plasma protein we measured that was significantly increased in PWH at high risk for SNAEs (*P* = 0.021, *q* = 0.159, Figure 3B). Although not statistically significant after *P* value adjustment, IL-10 was also elevated in the high-risk group (*P* = 0.039, *q* = 0.264). Of note, when stratified by country, IL-6 was nonsignificantly (*q* > 0.25) increased in PWH at high-risk of SNAEs (Supplemental Figure 5), and only reached statistical significance when the cohorts were combined.

We next compared the plasma metabolome of individuals at low and high risk of SNAE development. Risk group did not have a significant effect on the overall profile of metabolomic features when tested by PERMANOVA (*R*^2^ = 0.011, *q* = 0.782, Figure 3C). Overall, we found 13 metabolomic features that were significantly different between SNAE risk groups (*q* < 0.25, Figure 3D). Individuals with high risk of SNAEs had increased plasma concentrations of dicarboxylic acids (adipic acid and suberic acid), bile acids (cholic acid and 12-ketodeoxycholic acid), triglycerides (TG 20:1 34:3), polyamines (N1,N8-Di-Ac-spermidine), monoglycerides (MG 20:1), and lysophosphatidic acids (LPA 15:0), whereas those at low risk had higher concentrations of specific phosphatidic acids (PAs) (PA 14:0 14:1), PGs (PG 16:0 19:1), and PEs (PE P-18:0/20:3). Individuals at high risk also had a reduced ratio of chenodeoxycholic acid to cholic acid, and higher activity of imidazole propionic acid synthesis as measured by the ratio of imidazole propionic acid to histidine. GSEA showed enrichment of KEGG pathways for aminoacyl-tRNA biosynthesis, biosynthesis of amino acids, glycine/serine/threonine metabolism, and sphingolipid metabolism in patients with low risk, and enrichment of biosynthesis of unsaturated fatty acids in patients at high SNAE risk (*q* < 0.25, Figure 3E).

### Reduced commensal bacteria in gut biopsies of PWH at high risk of SNAEs.

**Figure 4:**
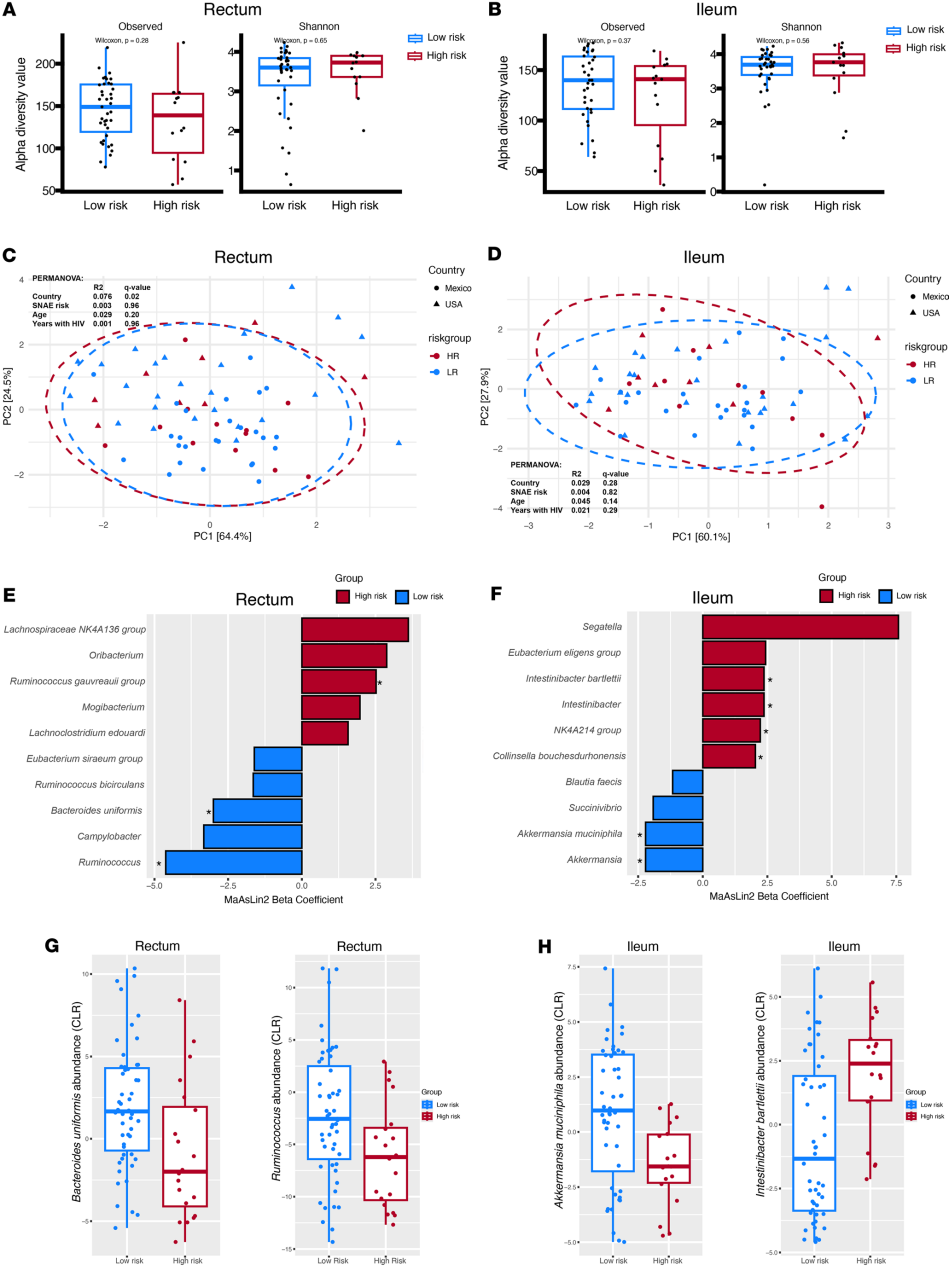
Differences in the ileal and rectal microbiomes of PWH at low or high risk of SNAEs. Alpha diversity metrics for rectum (**A**) and ileum (**B**) biopsies between SNAE risk groups. Beta diversity of rectum (**C**) and ileum (**D**) biopsies, tested by PERMANOVA using the robust Aitchison distance of genus-level taxa. Differential abundance of genus- and species-level taxa between SNAE risk groups, tested by MaAsLin2 for rectum (**E**) and ileum (**F**) biopsies, showing the top 10 differentially abundant taxa; asterisks denote FDR-adjusted *q* values ≤ 0.25. Representative box plots of differentially abundant taxa in the rectum (**G**) and ileum (**H**) biopsies.

We did not see any significant differences in alpha or beta diversity metrics in both the rectum and ileum of PWH with low versus high risk for SNAEs (Figure 4, A–D). However, differential abundance analysis revealed several genus and species level bacterial taxa that were associated with SNAE risk group. In the rectal biopsies, the *Ruminococcus gauvreauii* group was increased in PWH at high risk for SNAEs, while *Ruminococcus* and *Bacteroides uniformis* were both decreased (*q* < 0.25, Figure 4, E and G). In the ileal biopsies, *Intestinibacter, Intestinibacter bartlettii,* *Oscillospiraceae* NK4A214 group, and *Collinsella bouchesdurhonensis* were increased in PWH at high risk of SNAEs, while *Akkermansia* and *A*. *muciniphila* were decreased (*q* < 0.25, Figure 4, F and H). Notably, *Segatella* (formerly part of the *Prevotella* genus) was increased in the ileal biopsies of the high-risk SNAE group, though it did not reach statistical significance after adjusting for multiple comparisons (*P* = 0.016, *q* = 0.289, Figure 4F).

### Multi-omic machine learning discriminates between SNAE risk groups.

**Figure 5:**
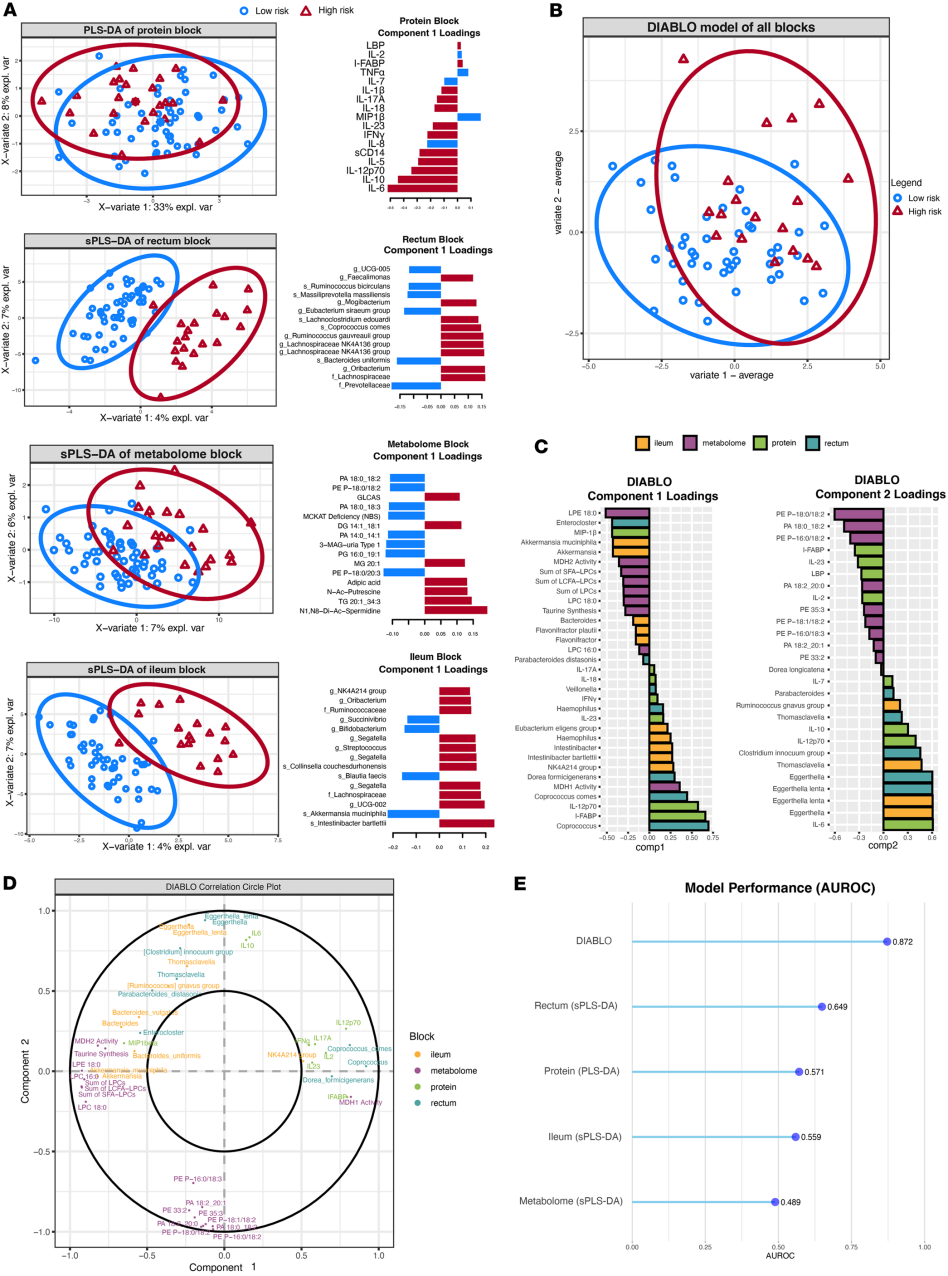
Supervised machine learning of SNAE risk groups. (**A**) Scores plot and top loading vectors on component 1 for the protein block PLS-DA, metabolome block sparse PLS-DA (sPLS-DA), rectum block sPLS-DA, and ileum block sPLS-DA. Loading vectors are colored by the group with the highest mean value. (**B**) Scores plot of the integrated DIABLO model, combining all 4 data blocks and showing the weighted average of components 1 and 2 according to their correlation with SNAE risk group. (**C**) Top loading scores on components 1 and 2 for each block in the DIABLO model; bars are colored by the corresponding block the variable belongs to. (**D**) Correlation circle plot of the DIABLO model, showing variables with correlations (>0.5) on components 1 and 2. (**E**) A variable’s location depicts both its correlation on each component and its correlation with other variables in proximity. Performance of each model in predicting SNAE risk based on AUROC, determined by 5-fold cross-validation repeated 50 times.

We next applied supervised machine learning methods from the mixOmics R package to evaluate how well each ome (i.e., proteins, metabolome, ileum, rectum) classified SNAE risk, to identify key discriminative variables, and to examine cross-omic relationships. For single-ome models, we used partial least squares discriminant analysis (PLS-DA) or sparse PLS-DA when there were more variables than samples (Figure 5A). To integrate all omes, we used Data Integration Analysis for Biomarker discovery using Latent variable approaches for Omics studies (DIABLO, Figure 5B).

Many of the top discriminatory features for each model were also identified in our differential analysis. For example, IL-6 and N1,N8-Di-Ac-spermidine were the top features for component 1 in the protein and metabolome models, both discriminatory for the high-risk SNAE group. Similarly, *A*. *muciniphila* and *Intestinibacter bartlettii* were the top features in the ileum model, in the direction of low- and high-risk SNAE groups, respectively. In the integrated DIABLO model, top features in the direction of PWH at high risk included rectum abundance of *Coprococcus* and *Coprococcus comes* as well as I-FAPB, IL-12p70, and MDH1 activity as measured by the ratio of malic to oxaloacetic acid (Figure 5, C and D). Top features in the direction of PWH at low risk included LPE 18:0, rectum *Enterocloster* abundance, MIP1-β, and ileum abundances of *Akkermansia* and *A*. *muciniphila*. On component 2, top features in the direction of PWH at high risk included IL-6 and both ileum and rectum abundances of *Eggerthella* and *Eggerthella lenta*, whereas features in the direction of PWH at low risk included the lipids PE P-18:0/18:2, PA 18:0/18:2, and PA P-16:0/18:2.

The ability of each model to discriminate between low- and high-risk groups was estimated by the mean area under the receiver operator curve (AUROC) from 5-fold cross-validation repeated 50 times. Individually, blocks performed modestly, with the rectal microbiome having the relative best performance (AUROC = 0.649, Figure 5E). In contrast, the integrated DIABLO model combining all omes achieved the top performance (AUROC = 0.872), indicating a strong ability to discriminate between low- and high-risk SNAE groups.

### Individual associations between omic features and T cell criteria.

**Figure 6:**
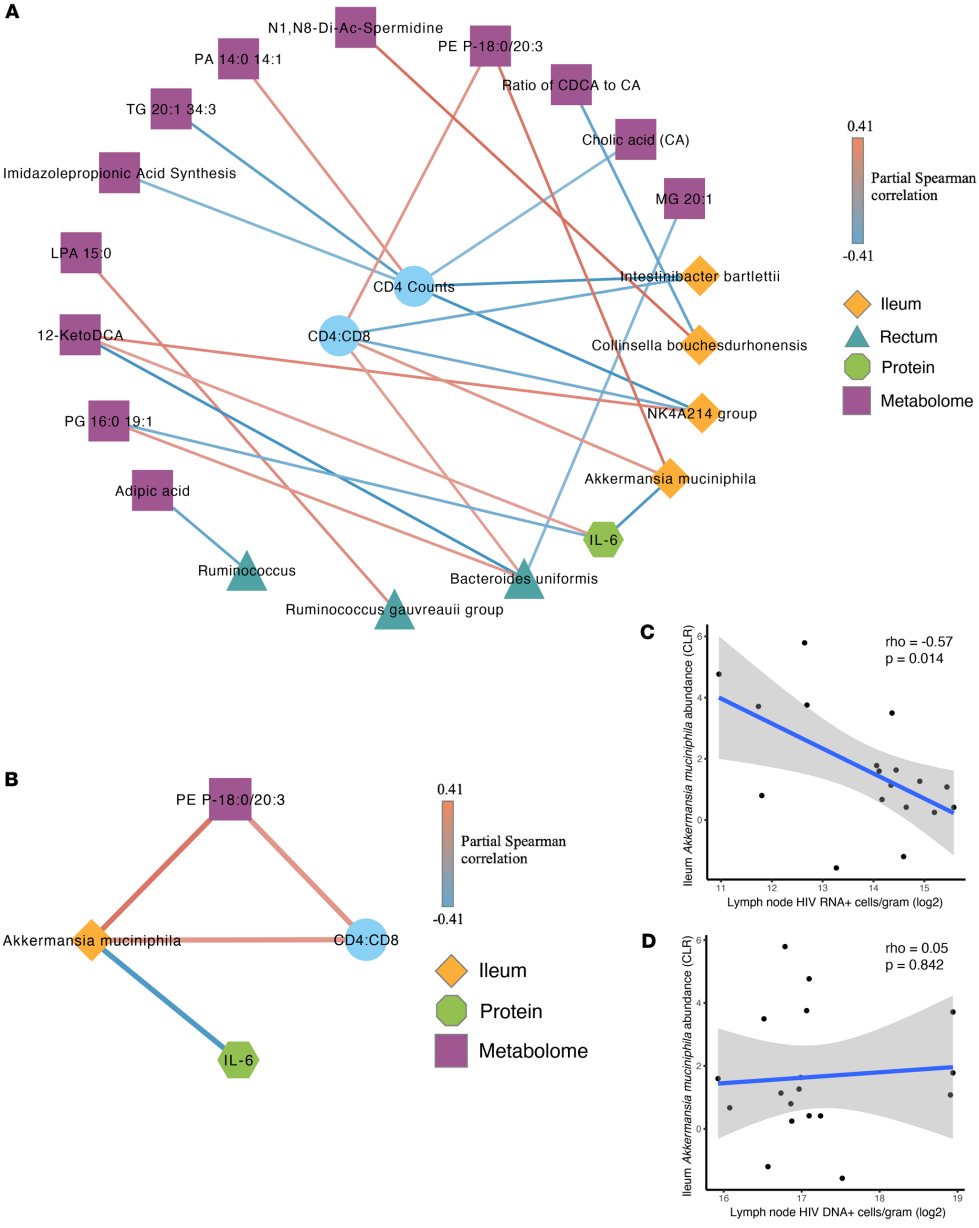
Partial correlations between variables of interest, CD4 counts, and the CD4/CD8 ratio. (**A**) Network of partial Spearman’s correlations between variables that were significantly different between SNAE risk groups, adjusted for country, age, and years living with HIV. (**B**) Selected correlations between bacterial taxa and the CD4/CD8 ratio with all connected nodes, highlighting potential mechanisms involved in driving immune activation. All correlations with an FDR-adjusted *q* ≤ 0.25 and nominal *P* ≤ 0.05 are shown. (**C** and **D**) Spearman’s correlations between ileum *A*. *muciniphila* abundance and HIV RNA^+^ (**C**) and DNA^+^ (**D**) cells/gram in lymph node tissue.

Because our high-risk SNAE group included criteria for both CD4^+^ T cell counts and the CD4/CD8 ratio, we next sought to understand how each biomarker was correlated with individual T cell criteria. We performed partial Spearman’s correlations between each biomarker and CD4^+^ T cell counts, CD8^+^ T cell counts, and the CD4/CD8 ratio, adjusting for the effect of country, age, and years living with HIV (Figure 6A). To reduce the number of comparisons, we focused only on biomarkers that were significantly different between our low- and high-risk groups. Notably, CD4^+^ T cell counts were negatively correlated with the ileum abundance of both *Intestinibacter bartlettii* (*r* = –0.405, *P* = 0.001, *q* = 0.011) and the NK4A214 group (*r* = –0.41, *P* < 0.001, *q* = 0.011). These taxa were similarly negatively correlated with the CD4/CD8 ratio (*r* = –0.312, *P* = 0.014, *q* = 0.15 and *r* = –0.305, *P* = 0.016, *q* = 0.15 respectively). In the opposite direction, ileum abundance of *A*. *muciniphila* and rectum abundance of *Bacteroides uniformis* were positively correlated with the CD4/CD8 ratio (*r* = 0.264, *P* = 0.038, *q* = 0.164 and *r* = 0.248, *P* = 0.043, *q* = 0.164, respectively). In the metabolomic data, CD4^+^ T cell counts were negatively correlated with TG 20:1 34:4 (*r* = –0.359, *P* = 0.002, *q* = 0.012), imidazole propionic acid synthesis (*r* = –0.269, *P* = 0.021, *q* = 0.089), and cholic acid (*r* = –0.253, *P* = 0.031, *q* = 0.107), while positively correlated with PA 14:0 14:1 (*r* = 0.274, *P* = 0.019, *q* = 0.089). The CD4/CD8 ratio was positively correlated with PE P-18:0/20:3 (*r* = 0.269, *P* = 0.021, *q* = 0.15). We did not observe any significant correlations with CD8^+^ T cell counts.

### Correlation analysis highlights the potential role of A. muciniphila in reducing immune activation via IL-6 and the active HIV reservoir.

We next performed partial correlations between these omic layers to understand how the relationships between them may be driving SNAE risk (Figure 6, A and B). Interestingly, we observed a negative correlation between ileum *A*. *muciniphila* abundance and plasma IL-6 concentrations (*r* = –0.388, *P* = 0.002, *q* = 0.007), with the same pattern observed when stratified by country (Supplemental Figure 6). IL-6 was further positively correlated with the secondary bile acid 12-ketodeoxycholic acid (*r* = 0.247, *P* = 0.035, *q* = 0.23) and negatively correlated with PG 16:0 19:1 (*r* = –0.302, *P* = 0.009, *q* = 0.121). Rectum *Bacteroides uniformis* abundance was similarly positively correlated with PG 16:0 19:1 (*r* = 0.269, *P* = 0.028, *q* = 0.242) and negatively correlated with 12-ketodeoxycholic acid (*r* = –0.415, *P* < 0.001, *q* = 0.019). Other notable correlations included a positive correlation between ileum *Collinsella bouchesdurhonensis* abundance and N1, N8-Di-Ac-spermidine (*r* = 0.375, *P* = 0.003, *q* = 0.118), and a positive correlation between *A*. *muciniphila* and PE P-18:0/20:3 (*r* = 0.354, *P* = 0.005, *q* = 0.112).

Given the negative correlation we observed between *A*. *muciniphila* and plasma IL-6, we next asked whether ileal *A*. *muciniphila* abundance was associated with the HIV reservoir. For a subset (*n* = 20) of the US participants, we were able to obtain lymph node tissue and measure the quantity of HIV RNA-producing cells and proviral DNA^+^ cells, as we have done previously (13, 18). Interestingly, ileal *A*. *muciniphila* abundance was negatively correlated with the number of HIV RNA^+^ cells/gram of lymph node tissue (Spearman’s *r* = –0.57, *P* = 0.014, Figure 6C), though was not correlated with the number of HIV DNA^+^ cells/gram (Spearman’s *r* = 0.05, *P* = 0.842, Figure 6D). Further, we did not observe a significant correlation between either IL-6 or IL-10 plasma concentrations and lymph node HIV reservoir measures (Supplemental Figure 7). Collectively, these findings suggest that *A*. *muciniphila* may reduce SNAE risk through a potential mechanism involving suppression of IL-6 and HIV RNA production in latently infected cells.

### Multi-omic SNAE risk signatures contextualized in PWoH.

**Figure 7:**
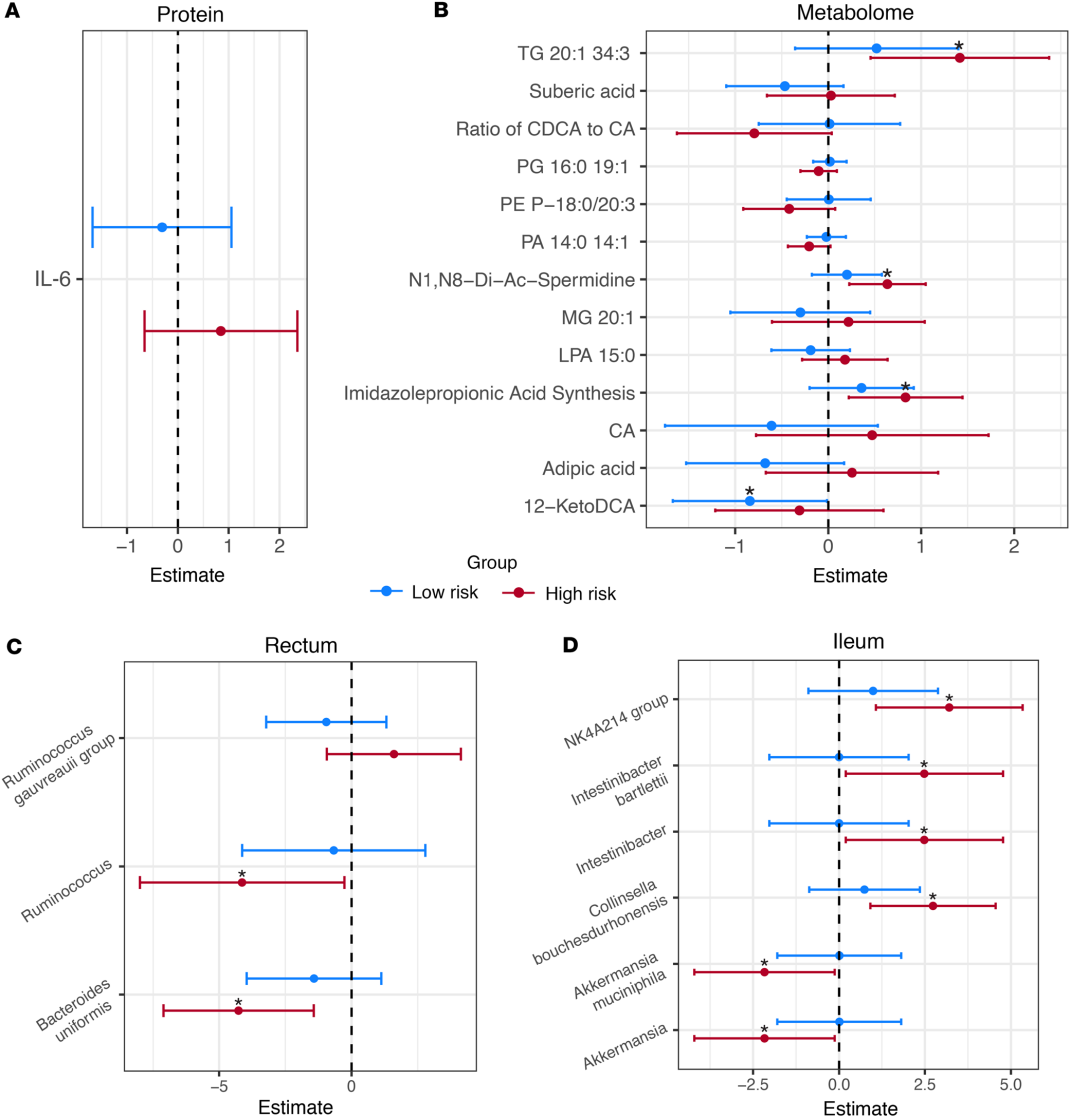
Comparison of SNAE risk groups to people without HIV. Variables that were significantly different between SNAE risk groups (*q* < 0.25) were compared against a PWoH reference group (*n* = 10, *n* = 5 per country) using linear regression adjusted for age and country. Panels display the linear model estimate and 95% CI for each SNAE risk group relative to PWoH for log_2_-transformed plasma IL-6 (**A**), log_2_-transformed metabolomic features (**B**), and CLR-transformed rectal (**C**) and ileal (**D**) microbiome taxa abundances, with microbiome estimates derived from MaAsLin2. Asterisks denote nominally significant differences from PWoH (*P* < 0.05).

To assess whether the observed differences between SNAE risk groups reflected true biological signals rather than confounding between groups, we compared the significantly different variables to a group of PWoH (*n* = 10) from both the United States and Mexico (*n* = 5 per country) using linear regression adjusted for age and country. Although limited statistical power likely hindered significance in many comparisons with PWoH, nearly all variables followed the expected ordinal trend, whereby features elevated in the high-risk relative to low-risk group were also higher than in PWoH and vice versa. For example, IL-6 was nonsignificantly elevated in the high-risk group relative to PWoH (β = 0.85, *P* = 0.272) and nonsignificantly reduced in the low-risk group (β = –0.31, *P* = 0.657, Figure 7A). Among metabolomic features, N1,N8-Di-Ac-spermidine, TG 20:1 34:3, and imidazole propionic acid synthesis were each significantly higher in the high-risk group compared with PWoH (*P* < 0.05, Figure 7B). Microbiome trends were similarly conserved, with *Ruminococcus* and *Bacteroides uniformis* significantly reduced in the high-risk group relative to PWoH in the rectum (*P* < 0.05, Figure 7C). In the ileum, *Akkermansia* and *A*. *muciniphila* were likewise reduced in the high-risk group, while the NK4A214 group, *Intestinibacter*, *Intestinibacter bartlettii*, and *Collinsella bouchesdurhonensis* were all significantly increased compared with PWoH (*P* < 0.05, Figure 7D). The only exception to these patterns was 12-ketodeoxycholic acid, which was lowest in the low-risk group, followed by the high-risk group and PWoH. Collectively, the significant differences observed between the high-risk group and PWoH in key metabolomic features and microbiome taxa, combined with the directional consistency of nearly all remaining variables, supports the biological plausibility of these signatures as indicators of SNAE risk.

## Discussion

In this multisite analysis, we first examined country-level differences within our HIV cohort. Country of enrollment was the largest determinant of variation across plasma proteins, the plasma metabolome, and the gastrointestinal microbiome. Participants from Mexico exhibited higher plasma cytokines involved in adaptive and mucosal immunity (IL-12p70, IL-17A) and markers of gut epithelial injury and microbial translocation (I-FABP, sCD14), collectively suggesting greater mucosal immune activation in this cohort. IL-12p70 and IL-17A support Th1-driven antiviral responses and gut barrier integrity (19, 20), functions that are impaired in HIV infection. These immune features coincided with substantially greater mucosal abundance of *Escherichia-Shigella,* a common intestinal pathogen in developing countries (21), in both ileal and rectal biopsies, which we suspected could be leading to increased mucosal inflammation in PWH from Mexico, although this taxon did not directly correlate with these plasma markers. In contrast, US participants had higher levels of MIP-1β, IL-8, and IL-7, indicating distinct patterns of immune activation between countries that may reflect differences in HIV care, environmental exposures or coinfections; however, CMV serostatus did not differ between them.

The ileal and rectal microbiomes were dominated by *Bacillota* and *Bacteroidota*, consistent with previous reports (22). Rectal biopsy composition differed most strongly by country, although alpha diversity metrics did not vary. Given differences in baseline characteristics between countries (e.g., race, sex) and unmeasured effects such as diet, it is not surprising that the gastrointestinal microbiomes of these countries would significantly differ. Some of the most notable differences we observed were the increased abundance of *Escherichia-Shigella* and the *Eubacterium eligens* group in participants from Mexico in both biopsy sites. In comparison, US participants displayed increased *Parabacteroides distasonis* in both biopsy sites. *Escherichia-Shigella* is a Gram-negative genus whose members include diarrheal pathogens and potent inducers of inflammatory signaling (21), whereas the *Eubacterium eligens* group has been linked to reduced visceral fat accumulation and shown to be protective for pulmonary arterial hypertension (23, 24).

Country effects were most pronounced in the plasma metabolome. US participants had significantly higher concentrations of numerous lipid classes. Compared with Mexican men living in Mexico, Mexican men living in the United States are at a greater risk of obesity, abdominal obesity, and diabetes, which may be reflective of the lipid changes we see (25). Consistent with this, sphingomyelins, phosphatidylcholines, and lyso-phosphatidylcholines, all previously linked to obesity (26), were enriched in our US cohort. These differences could potentially reflect differences in BMI between countries. However, BMI was unavailable as a covariate in our analysis and is a limitation in interpreting these findings. US-associated metabolites in our study, such as taurine and oxaloacetic acid, also aligned with a previous study comparing the plasma metabolome of US-born versus foreign-born (primarily from Mexico) Hispanic men (27), attributed to dietary differences. Mexico participants exhibited higher levels of medium-chain acylcarnitines (C8, C10, C10:1, C12:1), several of which have been shown to increase after moderate-intensity exercise, promote lipid oxidation, and decrease with oral food intake (28), potentially reflecting differences in physical activity, energy metabolism, and dietary practices between cohorts.

Understanding how geography influences multi-omic profiles in PWH is essential for capturing the global impact of HIV. We therefore examined whether PWH from the United States and Mexico who were at high risk for SNAEs, based on low CD4^+^ T cell counts and CD4/CD8 ratios, shared consistent biomarkers. Although SNAE risk explained far less variance than country of enrollment, a clear pattern emerged involving systemic inflammation, microbial dysbiosis, and metabolomic alterations suggestive of mitochondrial dysfunction.

First, we observed increased plasma IL-6 concentrations in participants at high risk for SNAEs, which was consistent when stratified by country, in agreement with previous studies (29, 30), and followed the expected ordinal trend relative to PWoH, collectively indicating an elevated level of systemic inflammation in these individuals. Further, IL-6 was a topmost discriminatory variable in the supervised models built to discriminate between low- and high-risk SNAE groups. Similarly, other cytokines including IL-10 and IL-12p70 were nonsignificantly elevated in the high-risk group but were top cytokines in contributing to class separation. Given the known increase of IL-10 with HIV progression (31) and its dual role in moderating inflammation (32, 33) and supporting reservoir persistence (34), this may reflect a compensatory yet potentially pathogenic response.

Among plasma metabolites, dicarboxylic acids, especially suberic and adipic acid, were markedly increased in the high-risk SNAE group. These products of ω-oxidation accumulate when mitochondrial β-oxidation is impaired or overloaded, as observed in fatty-acid oxidation disorders such as medium-chain acyl-coenzyme A dehydrogenase deficiency (35). Because β-oxidation normally fuels ATP generation via the electron transport chain, diversion toward ω-oxidation and the buildup of dicarboxylic acids indicate mitochondrial stress (36, 37). Thus, higher adipic and suberic acids in high-risk PWH support the presence of disrupted β-oxidation and mitochondrial dysfunction. Several mechanisms could contribute to this dysfunction. Carnitine deficiency, which is more common in PWH (38), can restrict β-oxidation by limiting fatty acid transport into mitochondria. Inflammation may further impair β-oxidation by suppressing PGC-1α, a key regulator of mitochondrial biogenesis and metabolic gene expression (39). Notably, the HIV Tat protein has been shown to downregulate PGC-1α, linking viral activity to mitochondrial defects observed in HIV-associated neurocognitive disorder (40). Elevated adipic and suberic acids may also exert direct cellular effects by inducing DNA damage in leukocytes (35), a process associated with CD4^+^ T cell apoptosis, immune senescence, and the heightened inflammaging characteristic of PWH (41). Importantly, these dicarboxylic acids are increased even in treatment-naive PWH compared with healthy controls (42), suggesting that their accumulation is not driven by ART.

Additional metabolites elevated in high-risk PWH included N1,N8-diacetylspermidine and primary bile acid cholic acid. N1,N8-diacetylspermidine has been identified as a tumor biomarker associated with poor cancer prognosis (43, 44), potentially reflecting the increased malignancy risk in this group. Cholic acid and the cholic acid/chenodeoxycholic acid ratio, both linked to liver fibrosis and inflammation (45, 46), were also higher in high-risk participants, and cholic acid negatively correlated with CD4^+^ T cell counts, suggesting a link between primary bile acids and T cell reconstitution. Further, imidazole propionic acid, an exclusive microbially derived metabolite derived from histidine, has been linked to dysbiosis and increased risk of cardiovascular disease (47) and type 2 diabetes (48). Here, imidazole propionic acid synthesis (ratio of imidazole propionic acid to histidine) was increased in the high-risk group and was also negatively correlated with both CD4^+^ T cell counts. Notably, N1,N8-diacetylspermidine, TG 20:1 34:3, and imidazole propionic acid synthesis were also significantly elevated in the high-risk group relative to PWoH, further supporting their relevance as markers of heightened SNAE risk. Several lipids were elevated in the low-risk group, most notably PE P-18:0/20:3, which positively correlated with both the CD4/CD8 ratio and ileal *A*. *muciniphila* abundance. This finding is of particular interest given that a separate PE lipid derived from the *A*. *muciniphila* cell membrane (PE a15:0-i15:0) was recently shown to promote homeostatic immune responses through low-level TLR2-TLR1 signaling (49), raising the possibility that other PE lipids associated with *A*. *muciniphila* have similar immunomodulatory effects.

The ileal and rectal microbiomes were similar between low- and high-risk SNAE groups, with no significant differences in alpha or beta diversity. However, several taxa differed in abundance, with notable reductions in commensal, short-chain fatty acid producers in the high-risk group, including *A*. *muciniphila* in the ileum and *Bacteroides uniformis* and *Ruminococcus* in the rectum, both of which were also significantly reduced relative to PWoH. These organisms are associated with antiinflammatory and beneficial effects, and *A*. *muciniphila* and *B*. *uniformis* are being developed as next-generation probiotics (50, 51). Both these taxa positively correlated with the CD4/CD8 ratio, whereas ileal *Intestinibacter bartlettii*, previously linked to irritable bowel syndrome (52), was negatively correlated with CD4^+^ counts and CD4/CD8 ratios. Notably, *A*. *muciniphila* abundance was also inversely associated with plasma IL-6 and HIV RNA-producing cells in lymph nodes, suggesting a potential role in limiting systemic inflammation and active HIV transcription. That this association was absent for proviral DNA+ cells in lymph nodes points toward an effect on latency reversal rather than latent reservoir size with important implications for how the gut microbiota may influence HIV persistence. Consistent with this, recent evidence that lymph node HIV RNA-producing cell frequency negatively correlates with the CD4/CD8 ratio suggests a broader relationship between *A*. *muciniphila* depletion, HIV transcriptional activity, and immune activation (53).

Probiotic or prebiotic interventions, especially in PWH at high risk of SNAEs, represents a potential strategy to improve immune recovery and mitigate comorbidity development. The use of *A*. *muciniphila* is particularly promising, given its link to a wide range of beneficial host effects relevant to HIV-associated inflammation including mitochondrial dysfunction, hepatic steatosis, and diabetes, in addition to a growing body of mechanistic evidence supporting these effects (54–61). No human studies have directly tested *A*. *muciniphila* supplementation in PWH, though a recently completed clinical trial (NCT04058392) utilized a prebiotic specifically meant to increase *A*. *muciniphila* abundance in PWH with a CD4/CD8 ratio less than 1, with the goal of reducing inflammation in these individuals. Supporting our observations in PWH, *A*. *muciniphila* has repeatedly been shown to inversely correlate with IL-6 (62–64), possibly via a process that inhibits IL-6/JAK/STAT signaling (65, 66). JAK/STAT inhibitors such as tofacitinib and ruxolitinib have been shown to reduce HIV replication in latently infected cell lines (67, 68); thus, it is conceivable that an *A*. *muciniphila–*mediated effect on JAK/STAT signaling could explain the negative correlation we observed with HIV RNA-producing cells in the lymph nodes. An alternative explanation is that increased *A*. *muciniphila* abundance reflects enhanced gut barrier integrity and reduced microbial translocation; however, we did not observe differences in LBP, sCD14, or I-FABP between low- and high-risk groups. Together with our findings, these data suggest *A*. *muciniphila* as a compelling candidate for a microbiome-based intervention to mitigate systemic inflammation and lower SNAE risk in PWH, supporting the need for clinical evaluation.

This study should be interpreted in the context of its limitations. First, the lack of metadata on covariates, including sexual orientation, BMI, diet, coinfections (beyond CMV), medication use, and comorbidities, represents a major constraint on the interpretation of the multi-omic readouts we measured, particularly given the well-established influence of diet on both metabolomic and microbiome profiles. Second, although most participants received similar ART regimens, the remaining individuals were sparsely distributed across many different ART combinations, preventing meaningful assessment of ART-specific effects. Further, CMV serostatus was positive for nearly all patients, but the absence of quantitative CMV replication data (e.g., IgG titers or CMV DNA) and other markers of systemic inflammation (e.g., C-reactive protein) limits our ability to fully evaluate inflammatory drivers, including the contribution of CMV to IL-6. Our SNAE risk groups were also limited by a modest sample size composed almost entirely of men, reducing generalizability. Given the exploratory nature of this work, we also opted to use a relaxed *P* value threshold for highly dimensional analyses, requiring an FDR-adjusted *q* value of 0.25 or less to prioritize hypothesis generation. Finally, our composite SNAE risk definition used CD4^+^ T cell counts and CD4/CD8 ratios as surrogate markers in the absence of longitudinally observed SNAE data. Future studies may benefit from investigating the biomarkers we observed with longitudinal SNAE data to associate them with specific health outcomes. These T cell criteria additionally represent bulk rather than antigen-specific populations, limiting our ability to disentangle more granular associations between immune cells and inflammatory biomarkers. Notwithstanding these limitations, many of our findings are consistent with previous research and support biologically plausible conclusions.

In summary, this cross-site multi-omic analysis revealed geography as the dominant driver of variation among PWH, with distinct inflammatory, metabolic, and microbial signatures observed between cohorts from the United States and Mexico. Despite these differences, individuals at high risk for SNAEs shared a consistent pattern of systemic inflammation, mitochondrial dysfunction, and loss of key commensal taxa. Elevated plasma IL-6, increased ω-oxidation products (adipic and suberic acids), and accumulation of bile acid and polyamine metabolites indicated persistent immune activation, hepatic stress, and dysfunctional mitochondrial β-oxidation. Along with reductions in mucosal short-chain fatty acid producers *A*. *muciniphila*, *B*. *uniformis*, and *Ruminococcus,* this finding suggests that microbiome disruption may contribute to this phenotype. In particular, *A*. *muciniphila* stands out as a mechanistically validated commensal that promotes mucosal integrity, enhances mitochondrial metabolism, and attenuates hepatic and systemic inflammation in experimental models (54, 55, 69). The depletion of *A*. *muciniphila* and its negative association with IL-6 and the active HIV reservoir underscores its potential therapeutic relevance. Exploring *A*. *muciniphila*–based therapeutics may represent a promising strategy to reduce inflammation and improve long-term health outcomes in PWH.

## Methods

### Sex as a biological variable.

Sex was considered as a biological variable. The study protocol permitted enrollment of all eligible participants regardless of sex, gender, race, or ethnicity. However, the cohort enrolled in this study was predominantly male.

### Participant enrollment.

This analysis included 82 PWH from Mexico (*n* = 48) and the United States (*n* = 34) enrolled in a study led by the University of Minnesota (UMN) between July 2021 and December 2023. Participants were enrolled at either the Center for Research in Infectious Disease (CIENI) of the National Institute of Respiratory Diseases (INER) in Mexico City or UMN in Minneapolis. Eligibility required age of 18 years or older, laboratory-confirmed HIV-1 infection, stable ART for over 12 months, plasma HIV RNA less than 48 copies/mL (isolated single blips up to 200 copies/mL were allowed if preceded and followed by undetectable viral load), and screening tests within institutional normal ranges. Exclusion criteria included pregnancy or breastfeeding, BMI of 30 kg/m^2^ or higher, and unsuitable for an inguinal lymph node biopsy (e.g., current use of anticoagulants, ≥3 prior lymph node biopsies). No participants were taking antibiotics at enrollment. A reference group of PWoH (*n* = 10; *n* = 5 per country) was recruited from the same sites for contextual comparison of SNAE risk–associated signatures. PWoH met the same age and general health eligibility criteria but had no laboratory-confirmed HIV infection and were not on ART.

### SNAE risk group definition.

CD4^+^ T cell count and CD4/CD8 ratio independently predict SNAE risk (10), with lower values being associated with higher risk. PWH were grouped as high or low SNAE risk based on these criteria. High SNAE risk was defined as having a CD4^+^ T cell count less than 350 cells/μL or a CD4/CD8 ratio less than 0.4 regardless of CD4^+^ T cell count after 2 years of ART, criteria associated with a nearly 3-fold increased SNAE risk over 5 years. Participants not meeting these criteria were defined as low risk.

### Sample collection.

Blood was collected in EDTA tubes prior to colonoscopy or lymph node biopsy with instructions to fast. Plasma was isolated by centrifugation within 30 minutes and stored at –80°C. Colonoscopies were conducted at the Endoscopy Center of the UMN Medical Center and at CIENI-INER after overnight bowel preparation. Pinch biopsies of the ileum and rectum were placed in QIAGEN Allprotect solution and stored at –80°C. Excisional inguinal lymph node biopsies were performed for a subset of US participants by UMN surgeons under local anesthesia via a 1-inch inguinal incision, with participants observed for 2 or more hours after the procedure. All procedures occurred within a 2-week period.

### Plasma protein measurements.

Concentrations of cytokines were measured in EDTA plasma using a 13-plex (IL-1β, IL-2, IL-5, IL-6, IL-7, IL-8, IL-10, IL-12p70, IL-17A, IL-23, TNF-α, IFN-γ, and MIP-1β) Luminex panel (HSTCMAG-28SK, MILLIPLEX) and by ELISA (IL-18) (DY318-05, R&D Systems). Biomarkers for gut barrier damage and microbial translocation were measured in EDTA plasma by ELISA including I-FABP (DFBP20, R&D Systems), sCD14 (QK383, R&D Systems), and LBP (Ab279407, Abcam). All processing was performed according to the kit manufacturer’s instructions. Values below the limit of detection for each analyte were replaced with the lowest detectable concentration divided by 2. CMV IgG serostatus was determined qualitatively using a commercially available ELISA (KA6698, Abnova).

### Plasma metabolomics.

The plasma metabolome was characterized in EDTA plasma using the commercially available MxP Quant 1000 kit (Biocrates). Lipids were measured by flow injection analysis–tandem mass spectrometry using a 5500 QTRAP instrument (AB Sciex) with an electrospray ionization source, and small molecules were measured by liquid chromatography–tandem mass spectrometry using a 5500+ Triple Quad instrument (AB Sciex). All concentrations were quantified using the Biocrates WebIDQ software. Metabolites that were present in less than 50% of samples were removed from the analysis, and any values that were below the limit of detection were replaced with the lowest detectable concentration divided by 2. A comprehensive list of the metabolites used in this analysis are provided (Supplemental Data 2), as well as any sum or ratio features (Supplemental Data 3).

### HIV reservoir quantification.

The HIV reservoir was quantified in 4% paraformaldehyde-fixed lymph node biopsies as previously described (13, 70, 71). Briefly, 5 to ten 5-μm sections separated by 20 μm were analyzed by RNAscope 2.5 (Advanced Cell Diagnostics) using in situ hybridization with HIV-specific probes from Advanced Cell Diagnostics to identify Clade B viruses (RNA antisense 416111 and DNA sense 425531). Quantitative image analysis was then used to determine the number of HIV RNA^+^ and DNA^+^ cells per tissue area.

### Microbiome analysis.

DNA was extracted from ileal and rectal biopsies using the QIAGEN PowerSoil Pro DNA extraction kit. The V4 rRNA region was amplified from template DNA using the 515F-806R primer set described in the Earth microbiome project (72), using a 30-cycle PCR. PCR products were then subjected to a second 10-cycle PCR to attach Illumina sequencing primer-compatible DNA regions as well as individual barcodes for each sample. Samples were all uniquely dual-indexed, as previously described (73). Sequencing libraries were loaded onto an Illumina NextSeq using a 2x300 P2 flow cell and sequenced to an average depth of 79,756 reads per sample.

Variable region primers were removed from the demultiplexed sequences using cutadapt (74) and then quality filtered, trimmed, denoised, and merged in R (v4.4.1) using the dada2 (75) package. Merged sequences were further filtered to only include those within the expected base-pair length of the V4 amplicon and then used to generate an amplicon sequence variant (ASV) table. Taxonomy was assigned using the SILVA v138.2 database (76). Unassigned ASVs or those belonging to archaea, Eukaryota, mitochondria, chloroplast, or *limnobacter* were discarded. The decontam (77) R package was then used to filter out any ASVs that were possible contaminants based on their prevalence in reagent-only negative control samples. ASVs were further filtered by requiring a prevalence of at least 50 reads (~0.001% median read depth) in 10% of samples, followed by removal of any samples with less than 1,000 total filtered reads. The filtered ASV tables for rectal and ileal biopsies were used for all downstream analyses.

For alpha diversity assessment, samples were first normalized by scaling with ranked subsampling (78). Metrics including Shannon and a number of observed taxa were compared at the ASV level between groups using Wilcoxon rank-sum tests with an α of 0.05 to determine significance. Beta diversity was tested by PERMANOVA with the adonis function in the vegan R package (79), using the Euclidean distance of the robust centered log-ratio (CLR) transformed (80) counts (i.e., robust Aitchison distance) for genus-level taxa. Effects included in the PERMANOVA model were evaluated individually using the “margin” argument and included SNAE risk group, country, age, and years living with HIV, using 999 permutations and an α of 0.05 after adjustment with the Benjamini-Hochberg (FDR) method (81). Differential abundance of taxa was determined using the MaAsLin2 R package (82) at the genus and species level using CLR transformed counts and the default FDR threshold of 0.25 for determining significance.

### Supervised machine learning analysis.

Supervised machine learning algorithms including PLS-DA (plasma proteins), sparse PLS-DA (ileum, rectum, and metabolome), and DIABLO (all omes) were implemented using the mixOmics R package (83) to characterize the prediction capability of each omic data type and to identify the top discriminatory features for SNAE risk. Models were tuned for the number of components and number of features to keep as appropriate for each model, using 5-fold cross-validation repeated 50 times. Performance of each model was assessed by the AUROC again by 5-fold cross-validation repeated 50 times. For the DIABLO model, a design matrix with a moderate correlation of 0.5 was used between each data type.

### Statistics.

Statistical analysis was performed using R. Serological biomarkers (e.g., cytokines, metabolites) were log-transformed prior to downstream analysis. Multiple linear regression was used to evaluate the effect of SNAE risk group (i.e., low risk versus high risk) and country on biomarker concentrations adjusting for age and years living with HIV. Partial Spearman’s correlations between biomarkers and T cell criteria (CD4^+^ T cell count, CD8^+^ T cell count, and CD4/CD8 ratio) were performed using the ppcor R package (84), adjusting for the effects of country, age, and years living with HIV. Comparisons and correlations were FDR-adjusted separately for each ome and considered significant with both a nominal *P* value of 0.05 or less and an adjusted *q* value of 0.25 or less. GSEA was performed using the Human Metabolome Database (HMDB) matched identifiers for each available metabolite with the multiGSEA and fgsea R packages (85, 86). Metabolites were supplied as a pre-ranked list of the *P* value multiplied by the metabolite’s fold change from the multiple linear regression models and matched to gene sets from the KEGG database, requiring a minimum gene set size of 3 and an adjusted *P* value of 0.25 or less to determine significance. Differences in the overall composition of the plasma metabolome or proteins were tested by PERMANOVA, similar to the microbiome analysis, using Euclidean distances of the respective log-transformed biomarkers. Differences in baseline characteristics were tested using Welch’s 2-tailed *t* tests or χ^2^ tests without *P* value adjustment.

### Study approval.

All participants gave written informed consent using IRB-approved forms. The UMN IRB approved the study (STUDY00009216) in the United States and the INER Ethics and Research Committees approved the study (C71-18) in Mexico.

### Data availability.

Sequencing data for the 16S rRNA analysis are available at NCBI’s BioProject (PRJNA1405126). Code used for this analysis is available from the corresponding author upon reasonable request. Data values for all applicable figures can be found in the Supplemental Data Values file.

## Author contributions

NRK, TWS, GSMD, and CMB conceptualized the study. NRK, TWS, GSMD, SAR, JA, and CMB supervised the study. NRK, TWS, and GS acquired funding. CMB, JA, KE, GW, CG, JR, RTC, TS, EC, SAR, FTR, MSN, LCR, KKOC, and OB conducted the investigation. CMB performed the formal analysis and visualization and wrote the original draft of the manuscript. NRK, ES, MB, NF, PH, MG, KE, SAR, and TWS performed manuscript review and editing.

## Conflict of interest

The authors have declared that no conflict of interest exists.

## Funding support

This work is the result of NIH funding, in whole or in part, and is subject to the NIH Public Access Policy. Through acceptance of this federal funding, the NIH has been given a right to make the work publicly available in PubMed Central.

- NIH (AI147912) to TWS.
- UMN Department of Surgery funds to NRK.
- CIENI-INER is supported by the Mexican government (Programa Presupuestal P016; Anexo 13 del Decreto del Presupuesto de Egresos de la Federación).

## Supplementary Material

Supplemental data

Supplemental data sets 1-4

Supporting data values

## Figures and Tables

**Table 1:**
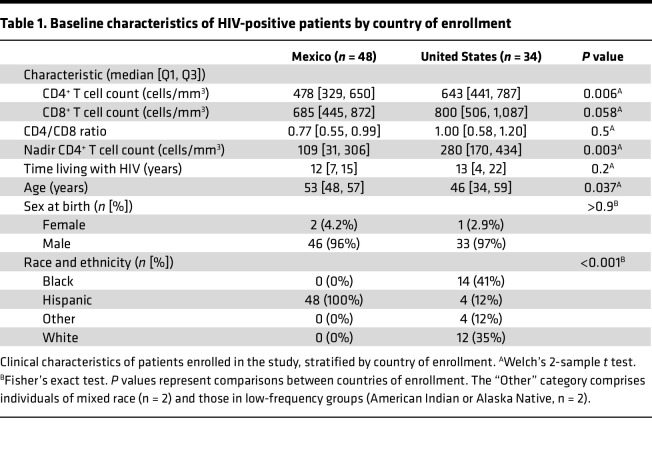
Baseline characteristics of HIV-positive patients by country of enrollment

**Table 2:**
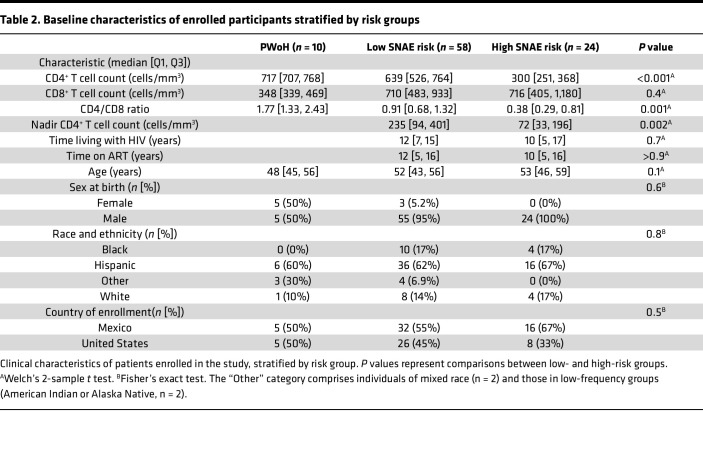
Baseline characteristics of enrolled participants stratified by risk groups

## Version 1 07/22/2026

Electronic publication
