# Supplementary material for: Multi-omics links microbial dysbiosis, systemic inflammation, and metabolomic disruptions to SNAE risk in treated HIV
Source: JCI Insight. 2026 Jul 22;11(14):e205379. doi: 10.1172/jci.insight.205379 (PMC13461162; doi:10.1172/jci.insight.205379)
Supplement: Supplementary file 1 [file jciinsight-11-205379-s348.pdf]

Supplemental Material:

Supplemental Table 1. CMV serostatus by country of enrollment

Characteristic	Mexico N = 48[1]	US N = 34[1]	p-value[2]
**CMV status**			0.4
Negative	0 (0%)	1 (2.9%)	
Positive	43 (100%)	33 (97%)	

[1]n (%)

[2]Fisher's exact test

Supplemental Table 2. CMV serostatus by SNAE risk group

Characteristic	Low risk N = 58[1]	High risk N = 24[1]	p-value[2]
**CMV status**			>0.9
Negative	1 (1.9%)	0 (0%)	
Positive	53 (98%)	23 (100%)	

[1]n (%)

[2]Fisher's exact test

Supplemental Table 3. ART regimens by country of enrollment

Characteristic	Mexico N = 48[1]	US N = 34[1]	p-value[2]
**ART regimen**			<0.001
2NRTI + 2INSTI + PI + NNRTI	1 (2.1%)	0 (0%)	
2NRTI + INSTI	45 (96%)	19 (58%)	
2NRTI + INSTI + Booster	0 (0%)	1 (3.0%)	
2NRTI + INSTI + PI	0 (0%)	1 (3.0%)	
2NRTI + INSTI + PI + Booster	1 (2.1%)	0 (0%)	
2NRTI + NNRTI	0 (0%)	1 (3.0%)	
2NRTI + PI + Booster	1 (2.1%)	0 (0%)	
NNRTI + INSTI	0 (0%)	9 (27%)	
NRTI + INSTI	0 (0%)	2 (6.1%)	

[1] n (%)

[2] Fisher's exact test

Supplemental Table 4. ART regimens by serious non-AIDs event risk group

Characteristic	Low SNAE risk<br>N = 58[1]	High SNAE risk<br>N = 24[1]	p-value[2]
**ART regimen**			0.5
2NRTI + 2INSTI + PI + NNRTI	0 (0%)	1 (4.2%)	
2NRTI + INSTI	45 (80%)	19 (79%)	
2NRTI + INSTI + Booster	1 (1.8%)	0 (0%)	
2NRTI + INSTI + PI	0 (0%)	1 (4.2%)	
2NRTI + INSTI + PI + Booster	1 (1.7%)	0 (0%)	
2NRTI + NNRTI	1 (1.8%)	0 (0%)	
2NRTI + PI + Booster	1 (1.8%)	0 (0%)	
NNRTI + INSTI	7 (13%)	2 (8.3%)	
NRTI + INSTI	1 (1.8%)	1 (4.2%)	

[1]n (%)

[2]Fisher's exact test

Supplemental Table 5. Clinical characteristics of the HIV-negative cohort by country

**Characteristic** (Median [Q1, Q3])	**Mexico** N = 5†	**US** N = 5	**p-value**
CD4$^+$ T cell count (cells/mm^3)	659 [531, 779]	722 [716, 768]	0.3[1]
CD8$^+$ T cell count (cells/mm^3)	343 [270, 347]	469 [434, 560]	0.14[1]
CD4/CD8 ratio	2.27 [1.72, 2.71]	1.53 [1.29, 1.77]	0.8[1]
Age (years)	56 [47, 60]	45 [45, 48]	0.11[1]
Sex at birth (n [%])			0.2[2]
Female	4 (80%)	1 (20%)	
Male	1 (20%)	4 (80%)	

†T-cell values not available for one individual from Mexico

[1]Welch's t-test

[2]Fisher's exact test

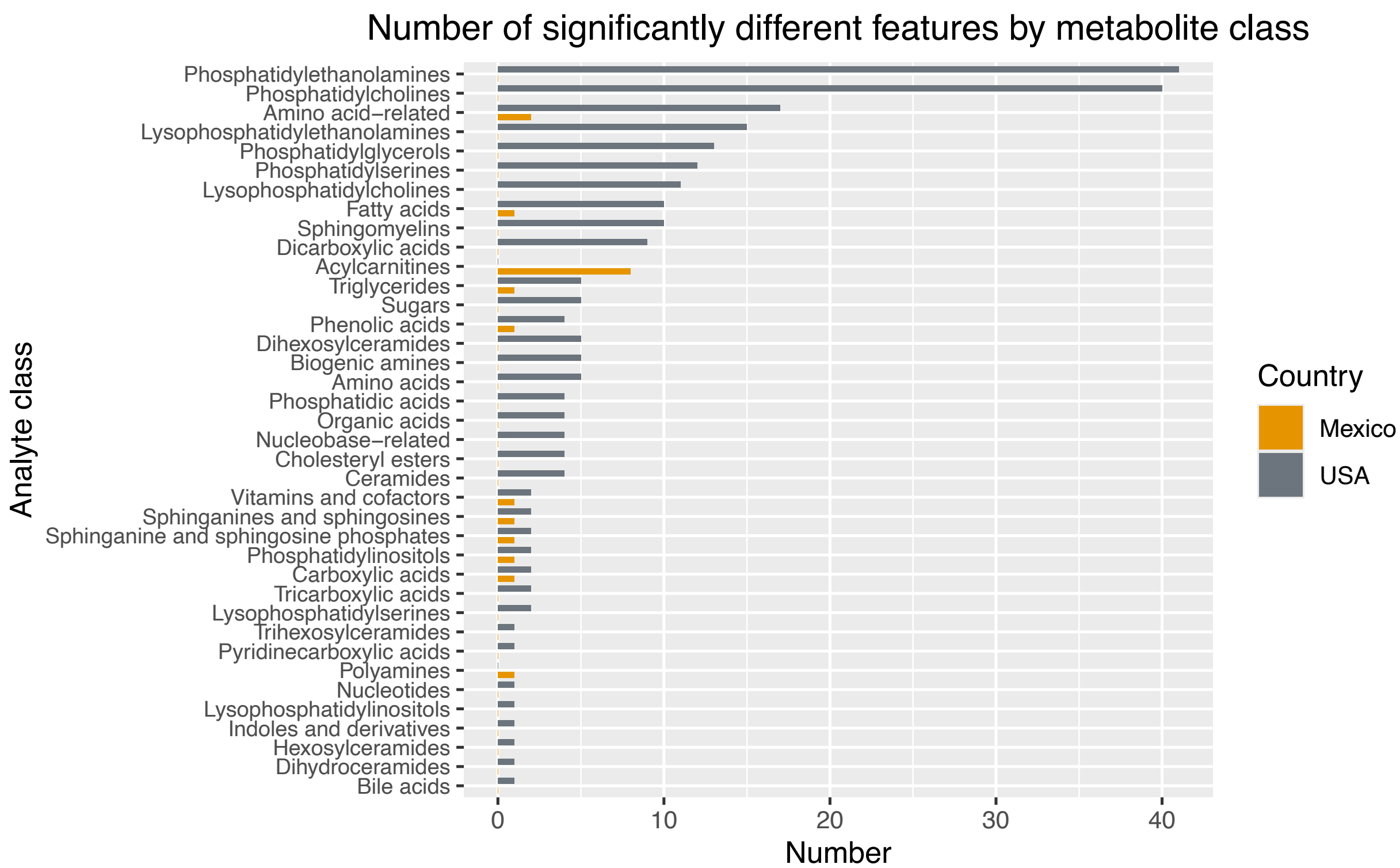

Supplemental Figure 1 Bar plot representing the number of significantly different metabolites (q-value < 0.25) per each analyte class and whether they were more represented in participants from the US or Mexico (determined by fold change).

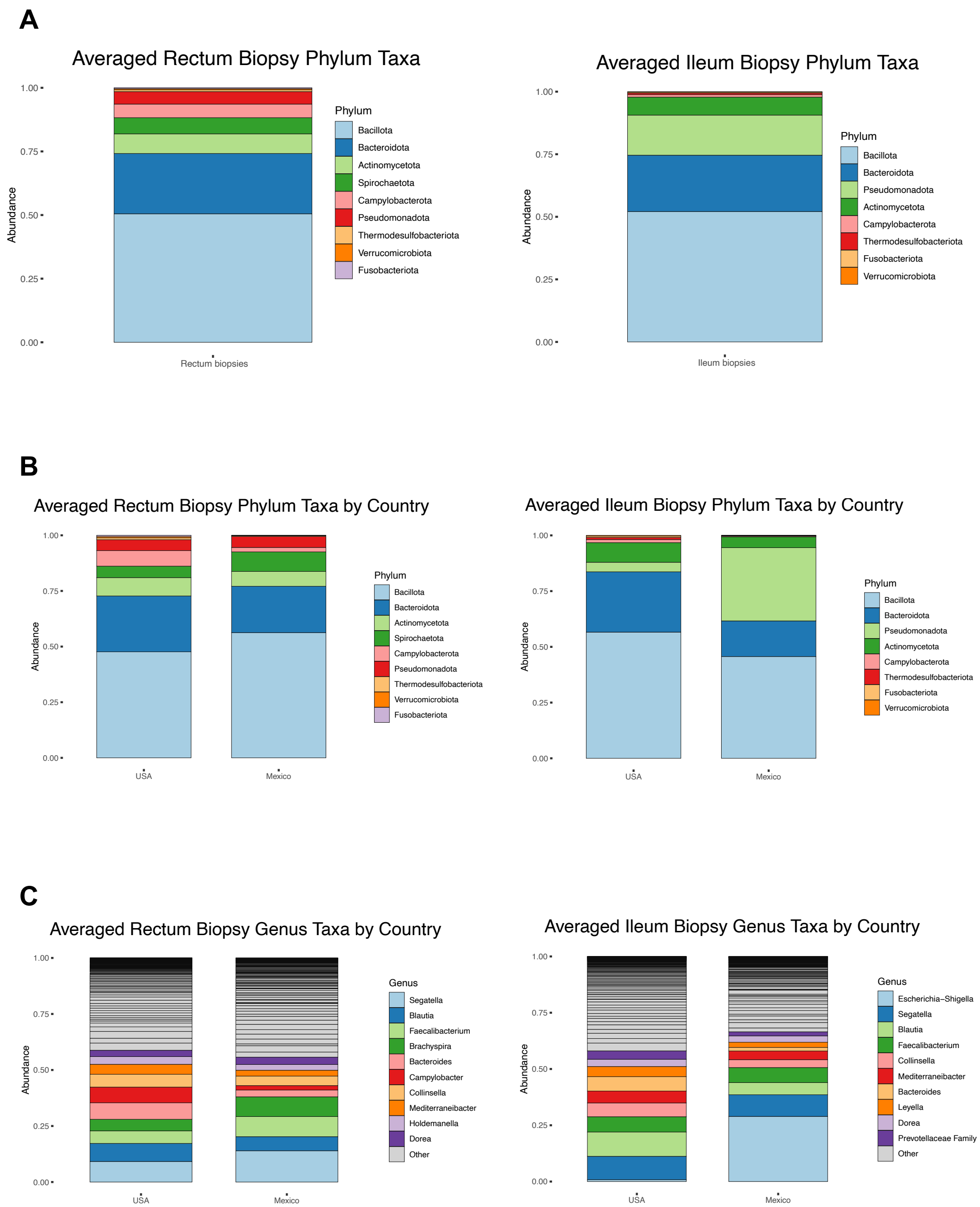

Supplemental Figure 2 Stacked bar plots showing the average relative abundance of bacterial taxa at the phylum level in rectal and ileal biopsies (A), stratified by country (B), and at the genus level (C).

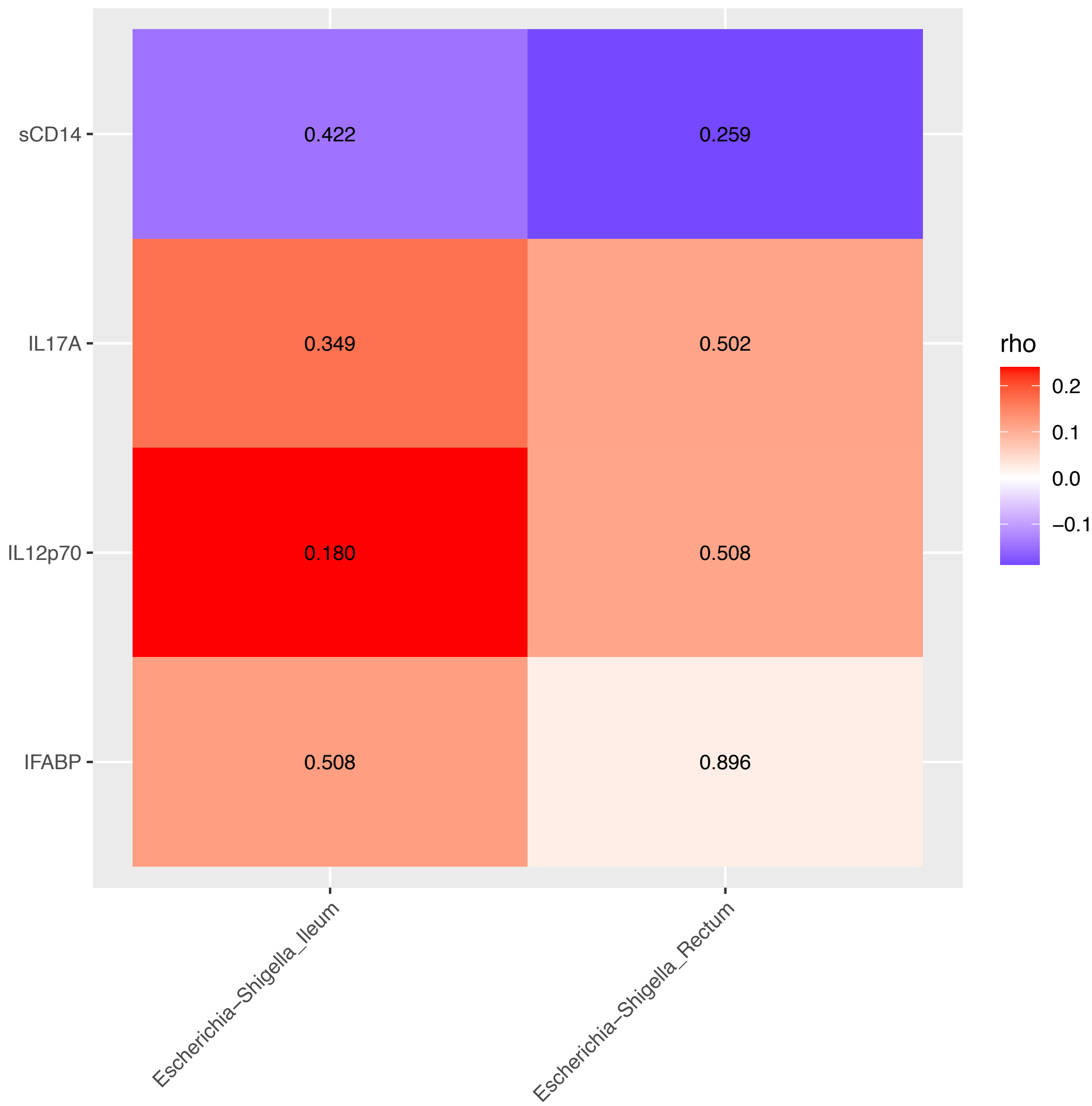

Supplemental Figure 3 Spearman correlations of *Escherichia-Shigella* abundance (CLR transformed) in the ileum and rectum biopsies of PWH enrolled from Mexico with mucosal cytokines and gut barrier damage markers. Values in each cell are the nominal p-values for each correlation.

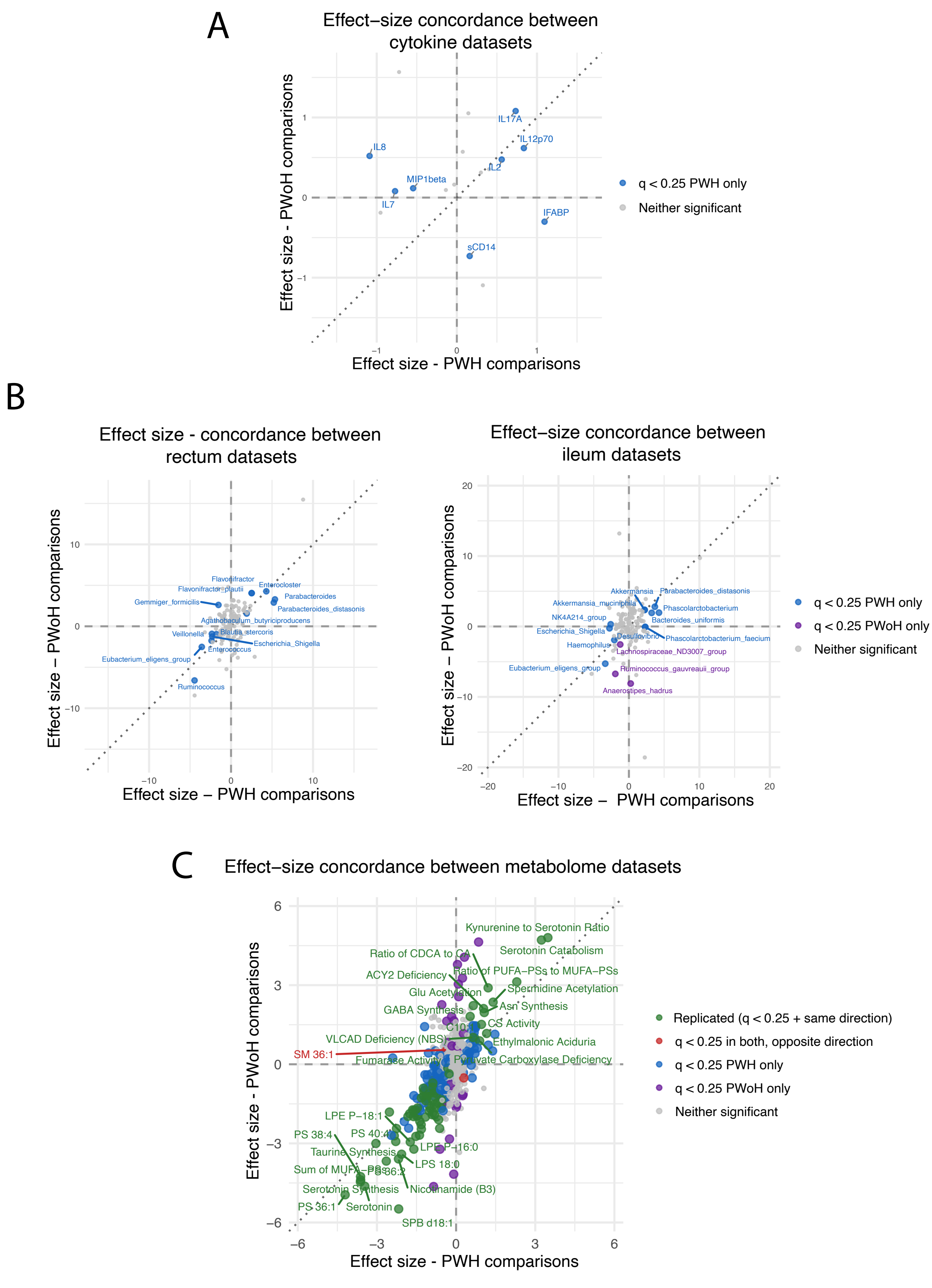

A
Effect–size concordance between cytokine datasets
Effect size - PWoH comparisons
Effect size - PWH comparisons
IL17A
IL12p70
IL2
IL8
MIP1beta
IL7
IFABP
sCD14
q < 0.25 PWH only
Neither significant
B
Effect size - concordance between rectum datasets
Effect size – PWoH comparisons
Effect size – PWH comparisons
Flavonifractor
Flavonifractor_plautii
Enterocloster
Parabacteroides
Parabacteroides_distasonis
Gemmiger_formicilis
Agathobaculum_butyriciproducens
Veillonella
Blautia_stercoris
Escherichia_Shigella
Enterococcus
Eubacterium_eligens_group
Ruminococcus
Effect–size concordance between ileum datasets
Effect size – PWoH comparisons
Effect size – PWH comparisons
Akkermansia
Parabacteroides_distasonis
Akkermansia_muciniphila
Phascolarctobacterium
NK4A214_group
Bacteroides_uniformis
Escherichia_Shigella
Desulfovibrio
Phascolarctobacterium_faecium
Haemophilus
Lachnospiraceae_ND3007_group
Eubacterium_eligens_group
Ruminococcus_gauvreauii_group
Anaerostipes_hadrus
q < 0.25 PWH only
q < 0.25 PWoH only
Neither significant
C
Effect–size concordance between metabolome datasets
Effect size - PWoH comparisons
Effect size - PWH comparisons
Kynurenine to Serotonin Ratio
Serotonin Catabolism
Ratio of CDCA to CA
Ratio of PUFA–PSs to MUFA–PSs
ACY2 Deficiency
Spermidine Acetylation
Glu Acetylation
Asn Synthesis
GABA Synthesis
CS Activity
C10
VLCAD Deficiency (NBS)
Ethylmalonic Aciduria
SM 36:1
Fumarase Activity
Pyruvate Carboxylase Deficiency
LPE P–18:1
PS 40:4
PS 38:4
LPE P–16:0
Taurine Synthesis
LPS 18:0
Sum of MUFA–PSs
PS 36:2
Serotonin Synthesis
Nicotinamide (B3)
PS 36:1
Serotonin
SPB d18:1
Replicated (q < 0.25 + same direction)
q < 0.25 in both, opposite direction
q < 0.25 PWH only
q < 0.25 PWoH only
Neither significant

Supplemental Figure 4 Concordance of differential abundance analysis for cytokines (A), rectal and ileal biopsy taxa abundances (B), and metabolomic features (C) between individuals from the US and Mexico and compared between PWH and PWoH. Values along the diagonal represent features that had similar effect sizes, reflecting agreement between cohorts.

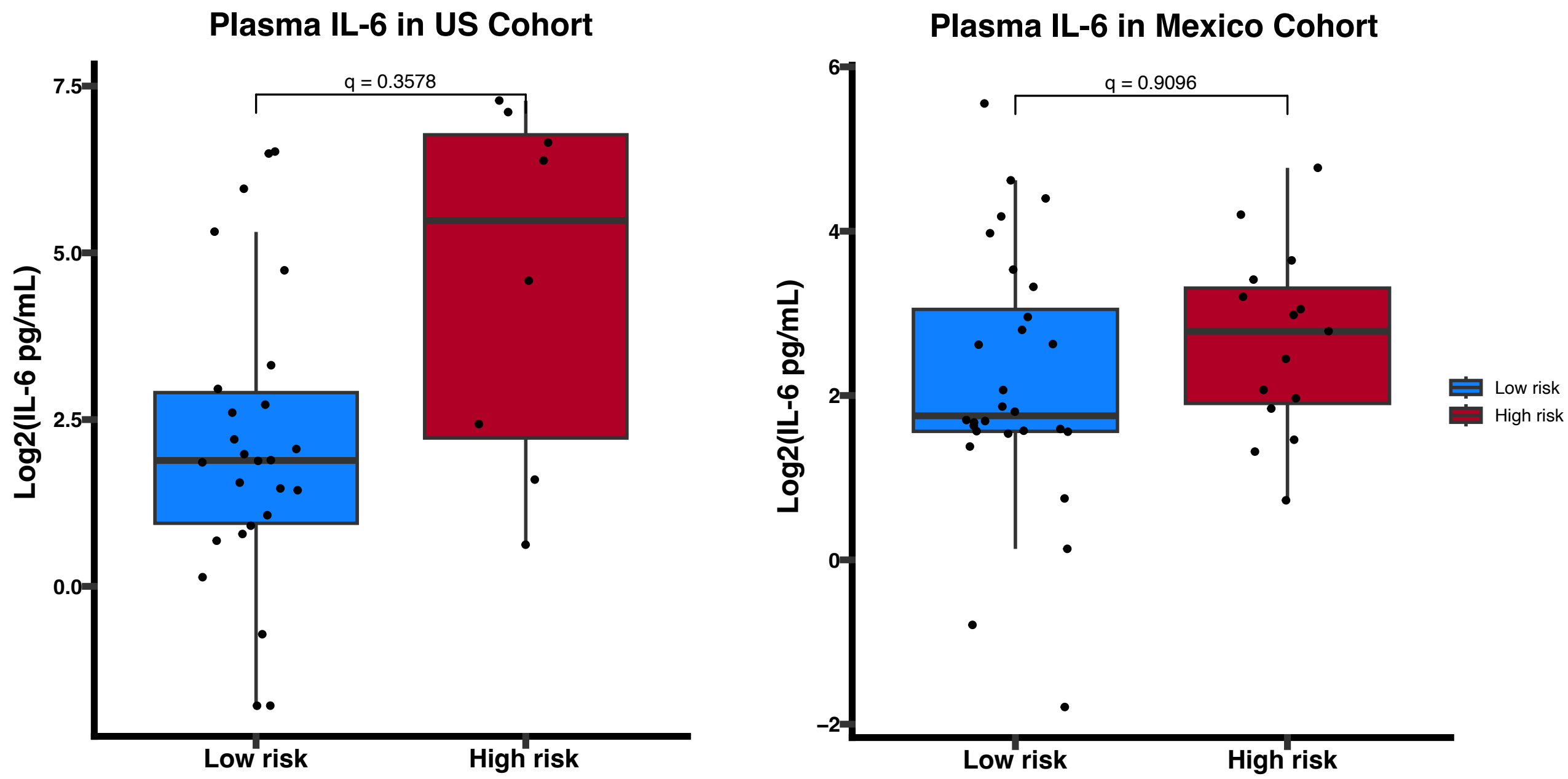

Supplemental Figure 5 Comparison of log2 transformed plasma IL-6 concentrations between low and high risk SNAE groups when stratified by country, performed using multiple linear regression adjusting for age and years living with HIV. IL-6 concentrations are non-significantly ($q > 0.25$) higher in the high-risk group within both cohorts of PWH and only becomes significant when combined.

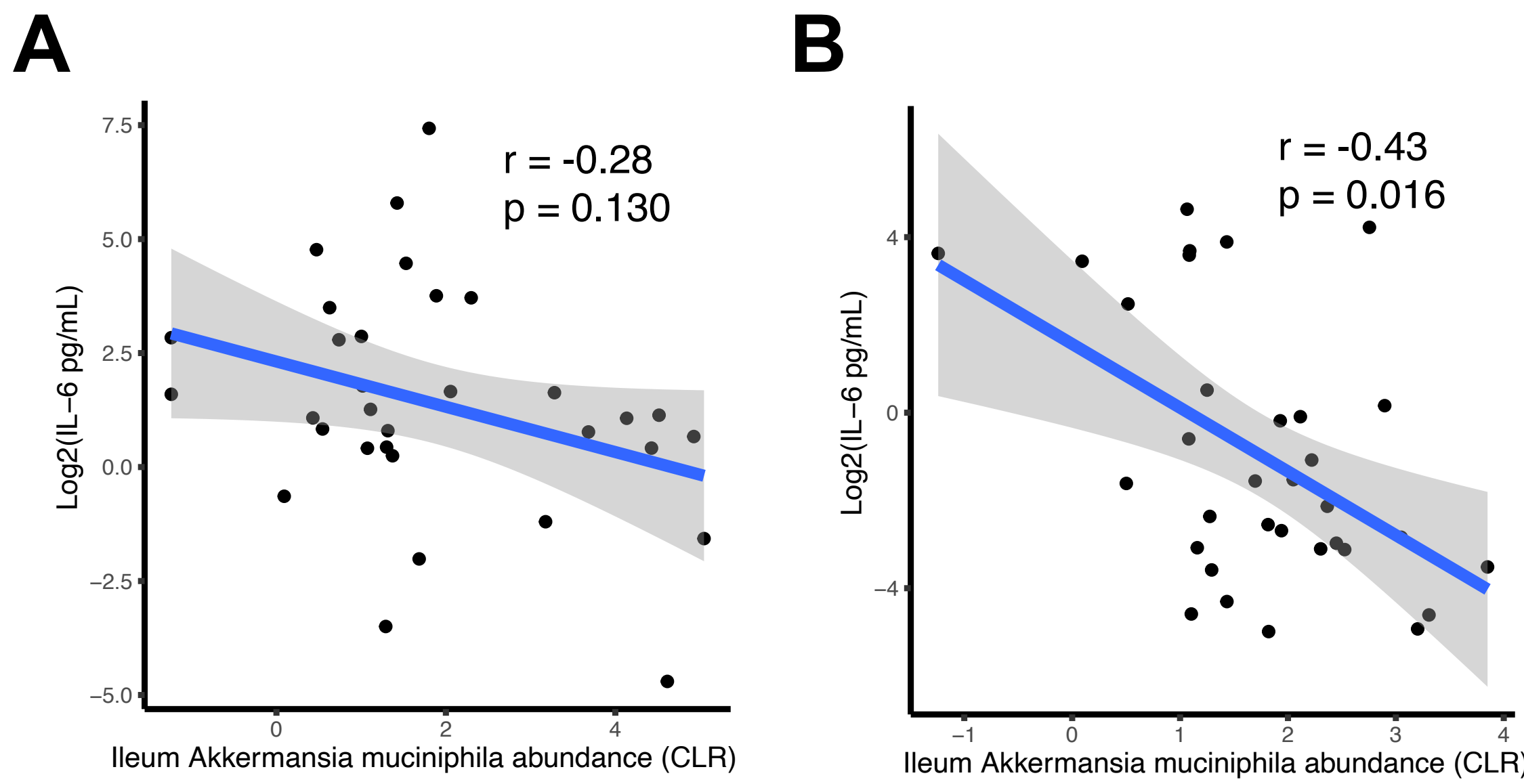

Supplemental Figure 6 Partial Spearman correlations (adjusted for age and years living with HIV) of log2 transformed plasma IL-6 concentrations with CLR transformed abundance of *Akkermansia muciniphila* in the ileum, stratified by US patients (A) and Mexico patients (B). A negative correlation is seen in both cohorts, though only statistically significant ($p < 0.05$) in the Mexico cohort.

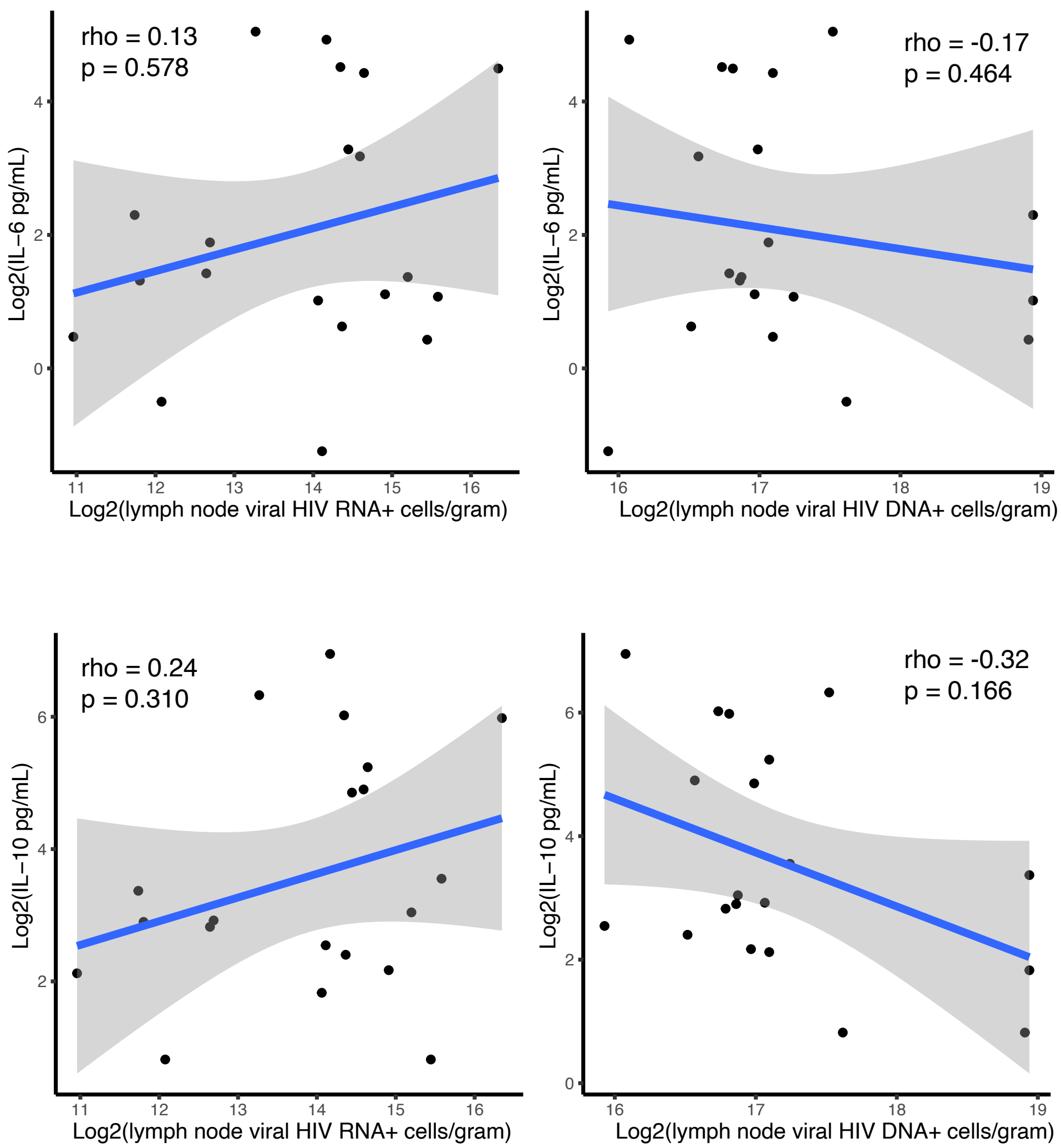

Supplemental Figure 7 Spearman correlations of log2 transformed plasma IL-6 and IL-10 concentrations with log2 transformed HIV reservoir measures in lymph nodes (RNA$^+$ and DNA$^+$ cells/gram).

Supplemental Data 1: List of country specific effects on metabolomic features

Supplemental Data 2: List of metabolites and their shorthand names

Supplemental Data 3: List of formulas used for sums and ratios calculated from available metabolites

Supplemental Data 4: List of analyte classes and number of molecules per class used in this analysis

SUPPLEMENTAL DATA 1

term	estimate	std.error	statistic	R2	Variable	q.value
CountryMexico	-2.259	0.118	-19.214	0.864	Taurine	2.60E-26
CountryMexico	-2.287	0.120	-19.122	0.862	Taurine Synthesis	2.60E-26
CountryMexico	-2.185	0.126	-17.400	0.840	Nicotinamide (B3)	4.40E-24
CountryMexico	-3.620	0.236	-15.359	0.806	PS 38:4	3.70E-21
CountryMexico	-1.649	0.112	-14.769	0.788	Sum of UFA-PSs	2.48E-20
CountryMexico	-1.316	0.096	-13.666	0.758	Sum of PUFA-PSs	1.24E-18
CountryMexico	-2.342	0.172	-13.612	0.765	PE P-18:0/22:4	1.31E-18
CountryMexico	-1.761	0.143	-12.307	0.726	PE P-16:0/22:4	1.79E-16
CountryMexico	-1.118	0.091	-12.219	0.748	Oxaloacetic acid	2.26E-16
CountryMexico	-3.039	0.266	-11.415	0.709	PS 40:4	5.10E-15
CountryMexico	-3.617	0.318	-11.380	0.723	Sum of MUFA-PSs	5.36E-15
CountryMexico	-1.600	0.143	-11.165	0.721	LPE P-16:0	1.14E-14
CountryMexico	-1.525	0.137	-11.154	0.692	LPE P-18:0	1.14E-14
CountryMexico	-1.489	0.139	-10.700	0.656	Spermidine	6.82E-14
CountryMexico	-1.449	0.138	-10.516	0.628	PE 42:7	1.36E-13
CountryMexico	-1.226	0.117	-10.491	0.638	PS 40:7	1.42E-13
CountryMexico	-1.467	0.140	-10.460	0.628	NeuAc	1.52E-13
CountryMexico	-2.646	0.257	-10.296	0.681	PS 36:2	2.83E-13
CountryMexico	-1.107	0.108	-10.226	0.660	2-OH-Glutaric acid	3.59E-13
CountryMexico	1.220	0.120	10.148	0.620	Sarcosine	4.73E-13
CountryMexico	1.403	0.139	10.093	0.653	Spermidine Acetylation	5.66E-13
CountryMexico	-1.614	0.160	-10.065	0.613	PE 40:4	6.07E-13
CountryMexico	-0.693	0.070	-9.943	0.608	Threonic acid	9.71E-13
CountryMexico	-1.031	0.105	-9.815	0.635	LPE 18:0	1.59E-12
CountryMexico	1.469	0.150	9.797	0.579	Sarcosine Synthesis from Choline	1.65E-12
CountryMexico	-1.242	0.128	-9.731	0.646	2-OH-Glutarate Synthesis	2.09E-12
CountryMexico	-4.205	0.433	-9.705	0.663	PS 36:1	2.25E-12
CountryMexico	-3.460	0.364	-9.497	0.627	Serotonin	5.21E-12
CountryMexico	3.479	0.370	9.410	0.640	Kynurenine to Serotonin Ratio	7.29E-12
CountryMexico	-3.627	0.389	-9.322	0.620	Serotonin Synthesis	1.02E-11
CountryMexico	-1.165	0.127	-9.177	0.592	Asp	1.77E-11
CountryMexico	-1.009	0.110	-9.179	0.589	PE 40:1	1.77E-11
CountryMexico	1.384	0.151	9.157	0.566	Sarcosine Synthesis from Gly	1.87E-11
CountryMexico	-2.527	0.281	-9.001	0.584	PS 40:5	3.52E-11
CountryMexico	-1.826	0.205	-8.923	0.547	PE P-18:0/22:3	4.77E-11
CountryMexico	-0.884	0.100	-8.831	0.608	SDH Activity	6.85E-11
CountryMexico	-0.832	0.097	-8.615	0.515	AHCys	1.63E-10
CountryMexico	3.235	0.375	8.618	0.605	Serotonin Catabolism	1.63E-10
CountryMexico	-1.736	0.206	-8.420	0.584	LPE P-18:1	3.65E-10

CountryMexico	-1.228	0.148	-8.276	0.541	PE P-18:0/22:5	6.58E-10
CountryMexico	-1.167	0.143	-8.172	0.496	PE 38:4	1.00E-09
CountryMexico	-1.148	0.141	-8.120	0.532	PE P-20:0/20:4	1.22E-09
CountryMexico	-2.066	0.255	-8.092	0.525	LPS 18:0	1.34E-09
CountryMexico	2.301	0.286	8.052	0.596	Ratio of PUFA-PSs to MUFA-PSs	1.56E-09
CountryMexico	0.808	0.101	8.004	0.600	MDH1 Activity	1.83E-09
CountryMexico	-0.808	0.101	-8.004	0.600	MDH2 Activity	1.83E-09
CountryMexico	-0.979	0.123	-7.981	0.479	PG 16:1_20:4	1.98E-09
CountryMexico	-0.314	0.040	-7.951	0.560	Fumarase Activity	2.20E-09
CountryMexico	-1.356	0.174	-7.785	0.536	PS 40:6	4.37E-09
CountryMexico	0.962	0.126	7.621	0.578	CS Activity	8.62E-09
CountryMexico	0.894	0.118	7.587	0.552	Pyruvate Carboxylase Deficiency	9.78E-09
CountryMexico	1.067	0.145	7.352	0.508	Asn Synthesis	2.61E-08
CountryMexico	-0.936	0.128	-7.305	0.493	Hex2Cer d18:1/22:0	3.13E-08
CountryMexico	1.041	0.143	7.285	0.535	ACY2 Deficiency	3.33E-08
CountryMexico	-0.662	0.091	-7.270	0.464	Aconitase Activity	3.48E-08
CountryMexico	-1.516	0.210	-7.219	0.449	Adenosine	4.24E-08
CountryMexico	-0.879	0.123	-7.121	0.463	Suc	6.21E-08
CountryMexico	-0.696	0.098	-7.123	0.471	PS 38:5	6.21E-08
CountryMexico	-0.736	0.104	-7.095	0.457	PE 38:2	6.82E-08
CountryMexico	-1.014	0.143	-7.091	0.479	Suc to a-Ketoglutarate Ratio	6.82E-08
CountryMexico	-0.818	0.115	-7.084	0.440	Isocitric acid	6.91E-08
CountryMexico	-2.456	0.351	-6.999	0.428	Benzoic Acid Conjugation	9.72E-08
CountryMexico	0.717	0.103	6.966	0.444	Glycolic Acid to Oxalic Acid Ratio	1.10E-07
CountryMexico	-0.932	0.135	-6.910	0.412	PE 42:8	1.37E-07
CountryMexico	-0.942	0.137	-6.895	0.501	Spermidine Synthesis	1.44E-07
CountryMexico	-1.611	0.235	-6.841	0.458	Histamine	1.78E-07
CountryMexico	-0.833	0.123	-6.784	0.404	AHCys to Leu Ratio	2.23E-07
CountryMexico	-1.537	0.232	-6.632	0.437	Histamine Synthesis	4.15E-07
CountryMexico	-1.163	0.177	-6.583	0.437	PA 18:2_18:3	5.02E-07
CountryMexico	-0.840	0.133	-6.304	0.417	PE P-16:0/22:5	1.58E-06
CountryMexico	-0.979	0.158	-6.189	0.370	Hex2Cer d18:1/24:0	2.50E-06
CountryMexico	-0.538	0.087	-6.172	0.390	PE 44:11	2.65E-06
CountryMexico	-1.601	0.263	-6.095	0.356	4-OH-Phenylacetic acid	3.58E-06
CountryMexico	-1.376	0.226	-6.081	0.419	PS 34:1	3.73E-06
CountryMexico	-0.716	0.118	-6.072	0.394	LPC 17:0	3.82E-06
CountryMexico	-1.927	0.319	-6.048	0.384	Carnosine	4.16E-06
CountryMexico	-0.548	0.091	-6.016	0.346	Aconitate to Citrate Ratio	4.69E-06
CountryMexico	-0.861	0.143	-6.013	0.390	LPC 20:4	4.69E-06
CountryMexico	-0.636	0.106	-6.002	0.366	PC O-40:1	4.85E-06
CountryMexico	-0.648	0.109	-5.941	0.362	LPE 17:0	6.13E-06

CountryMexico	-1.963	0.335	-5.867	0.353	HipAcid	8.17E-06
CountryMexico	-0.726	0.124	-5.850	0.364	Homovanillic acid	8.54E-06
CountryMexico	-0.774	0.132	-5.851	0.329	PE 40:7	8.54E-06
CountryMexico	-0.875	0.150	-5.841	0.433	Glu to a-Ketoglutarate Ratio	8.75E-06
CountryMexico	-1.853	0.318	-5.825	0.370	Carnosine Synthesis	9.24E-06
CountryMexico	-0.621	0.107	-5.809	0.335	PC O-34:0	9.76E-06
CountryMexico	-0.733	0.126	-5.803	0.325	PE 40:8	9.86E-06
CountryMexico	-1.382	0.240	-5.755	0.362	4-OH-HipAcid	1.18E-05
CountryMexico	-0.437	0.076	-5.741	0.347	PC O-40:4	1.24E-05
CountryMexico	1.675	0.300	5.592	0.365	b-Ala Synthesis	2.23E-05
CountryMexico	-0.705	0.127	-5.547	0.321	AconAcid	2.64E-05
CountryMexico	-0.892	0.162	-5.508	0.303	PE 40:5	3.03E-05
CountryMexico	-0.723	0.131	-5.507	0.302	Sum of PUFA-PEs	3.03E-05
CountryMexico	-2.403	0.439	-5.475	0.331	HPHPA	3.40E-05
CountryMexico	-0.591	0.108	-5.454	0.331	LPE 20:4	3.65E-05
CountryMexico	-0.612	0.113	-5.426	0.381	LPC 18:0	4.04E-05
CountryMexico	-0.367	0.068	-5.414	0.316	PC 32:0	4.19E-05
CountryMexico	-0.875	0.162	-5.402	0.351	PE P-18:0/20:4	4.36E-05
CountryMexico	-0.501	0.093	-5.385	0.365	Sum of SFA-LPCs	4.61E-05
CountryMexico	-0.448	0.084	-5.337	0.386	Cit	5.52E-05
CountryMexico	-0.574	0.108	-5.324	0.353	LPE 16:0	5.75E-05
CountryMexico	-0.501	0.094	-5.310	0.366	Sum of LPCs	6.00E-05
CountryMexico	-0.503	0.095	-5.307	0.366	Sum of LCFA-LPCs	6.02E-05
CountryMexico	-0.843	0.159	-5.297	0.332	PA 20:0_20:4	6.20E-05
CountryMexico	-0.453	0.086	-5.255	0.308	PC O-36:0	7.24E-05
CountryMexico	-1.798	0.346	-5.190	0.306	HipAcid Synthesis	9.23E-05
CountryMexico	-0.745	0.145	-5.148	0.282	AHCY Deficiency	0.000107659
CountryMexico	-0.478	0.093	-5.125	0.341	LPC 16:0	0.000116767
CountryMexico	-0.613	0.120	-5.118	0.324	PE 38:1	0.00011877
CountryMexico	-0.665	0.130	-5.107	0.272	3-HMGA	0.00012266
CountryMexico	-1.047	0.206	-5.093	0.285	cAMP	0.000128769
CountryMexico	-0.418	0.082	-5.090	0.308	PC O-42:1	0.000128779
CountryMexico	-0.740	0.146	-5.064	0.342	Glu	0.000141123
CountryMexico	-0.458	0.092	-4.992	0.330	CPS Deficiency (NBS)	0.000184839
CountryMexico	-0.511	0.103	-4.948	0.339	5-Oxo-Pro	0.000216076
CountryMexico	-0.233	0.048	-4.867	0.259	Glyceric acid	0.000292171
CountryMexico	-0.423	0.087	-4.845	0.252	PC O-38:4	0.000314408
CountryMexico	-0.434	0.090	-4.821	0.293	PC 28:1	0.000338109
CountryMexico	-0.542	0.114	-4.754	0.363	Sum of PUFA-LPCs	0.000431943
CountryMexico	-0.871	0.183	-4.747	0.334	PS 36:3	0.000439907
CountryMexico	1.157	0.244	4.738	0.344	Ethylmalonic Aciduria	0.000451918

CountryMexico	-0.608	0.129	-4.709	0.283	LPE 22:4	0.00049858
CountryMexico	-0.848	0.180	-4.701	0.255	Orotic acid	0.000509054
CountryMexico	-1.481	0.316	-4.681	0.244	PE P-18:0/16:1	0.000545451
CountryMexico	0.447	0.096	4.646	0.253	Biotin (B7)	0.000616769
CountryMexico	-0.513	0.112	-4.585	0.261	PE 20:0	0.000766375
CountryMexico	-0.496	0.109	-4.549	0.240	PS 38:7	0.000867852
CountryMexico	-0.497	0.109	-4.547	0.324	Sum of UFA-LPCs	0.000867852
CountryMexico	-0.389	0.086	-4.514	0.223	PC O-42:5	0.000971512
CountryMexico	0.463	0.103	4.478	0.270	Glycolic acid	0.001101189
CountryMexico	-0.704	0.157	-4.472	0.302	PE P-16:0/20:4	0.001114874
CountryMexico	-0.342	0.077	-4.467	0.259	3-Deoxyglucosone	0.001127902
CountryMexico	-0.354	0.080	-4.398	0.220	PC O-40:5	0.001439422
CountryMexico	-0.265	0.060	-4.394	0.251	Ratio of PI 18:0_20:4 to PIs	0.001446244
CountryMexico	-0.751	0.172	-4.376	0.228	N-Ac-Arg	0.001533001
CountryMexico	-0.675	0.155	-4.368	0.225	2-OH-2-Met-butyric acid	0.00156578
CountryMexico	-0.291	0.067	-4.341	0.242	Sum of MUFA-PCs O	0.001714008
CountryMexico	-0.460	0.106	-4.336	0.223	2-OH-Phenylacetic acid	0.001734352
CountryMexico	-0.547	0.127	-4.316	0.233	Putrescine	0.00184871
CountryMexico	-0.550	0.128	-4.304	0.212	PE 38:7	0.00191397
CountryMexico	-0.584	0.136	-4.301	0.223	3-OH-Glutaric acid	0.001919427
CountryMexico	-0.276	0.065	-4.263	0.258	N-Ac-Ser	0.002183872
CountryMexico	-0.608	0.143	-4.250	0.205	Hex2Cer d18:1/14:0	0.00227444
CountryMexico	-0.601	0.142	-4.228	0.209	PE 38:5	0.002443686
CountryMexico	-0.457	0.108	-4.224	0.204	PS 40:8	0.002463375
CountryMexico	-0.586	0.140	-4.197	0.203	PE 36:4	0.002692178
CountryMexico	1.215	0.290	4.182	0.316	Ratio of CDCA to CA	0.002815856
CountryMexico	0.642	0.156	4.121	0.221	MCAD Deficiency (NBS)	0.003466826
CountryMexico	-0.450	0.109	-4.114	0.208	PC O-44:6	0.003519807
CountryMexico	-0.348	0.085	-4.073	0.211	PC O-32:1	0.004039965
CountryMexico	-0.655	0.162	-4.036	0.188	PE 32:0	0.004503952
CountryMexico	-0.324	0.080	-4.039	0.250	PC O-36:1	0.004503952
CountryMexico	0.479	0.119	4.037	0.265	Betaine Synthesis	0.004503952
CountryMexico	0.236	0.059	4.018	0.255	SPBP d17:0	0.004734638
CountryMexico	0.575	0.143	4.019	0.250	Phenylacetate to PAGln Ratio	0.004734638
CountryMexico	-0.623	0.156	-4.004	0.268	Glutaminase Activity	0.004930193
CountryMexico	-0.762	0.190	-4.001	0.237	PE P-20:0/20:5	0.004959638
CountryMexico	-0.424	0.107	-3.966	0.217	PC O-30:0	0.005555707
CountryMexico	-0.875	0.221	-3.963	0.238	Fructose	0.005581785
CountryMexico	-0.498	0.126	-3.958	0.206	PG 20:4_22:3	0.005631842
CountryMexico	0.504	0.127	3.953	0.273	Ratio of Pro to Cit	0.00569505
CountryMexico	-1.316	0.334	-3.940	0.208	SPB d18:0	0.005921107

CountryMexico	-0.881	0.224	-3.937	0.239	Fructose to Glucose Ratio	0.00594052
CountryMexico	-0.277	0.071	-3.929	0.200	FA 20:0	0.006069419
CountryMexico	-2.176	0.558	-3.897	0.259	SPB d18:1	0.006700937
CountryMexico	-0.588	0.153	-3.858	0.242	Homovanillate to Vanillylmandelate Ratio	0.007597339
CountryMexico	0.408	0.106	3.841	0.190	FA 8:0 to FA 10:0 Ratio	0.007984715
CountryMexico	-0.402	0.106	-3.783	0.214	PC O-28:1	0.00942312
CountryMexico	-0.472	0.125	-3.773	0.184	PG 20:3_20:4	0.009706144
CountryMexico	-0.652	0.174	-3.757	0.176	PE 36:1	0.010166085
CountryMexico	0.512	0.136	3.755	0.226	Carnitine Uptake Defect (NBS)	0.010166085
CountryMexico	-0.909	0.243	-3.749	0.272	LPE P-20:0	0.010334332
CountryMexico	0.694	0.185	3.741	0.254	C10:1	0.010527096
CountryMexico	0.737	0.199	3.701	0.223	C10	0.011964854
CountryMexico	-0.455	0.123	-3.691	0.309	LPC 18:2	0.012316522
CountryMexico	0.730	0.198	3.689	0.212	C8	0.012328755
CountryMexico	-0.364	0.099	-3.683	0.174	SM 35:1	0.012467943
CountryMexico	-0.409	0.111	-3.681	0.216	Sum of MUFA-LPCs	0.012498794
CountryMexico	0.651	0.178	3.662	0.242	Glu Acetylation	0.013240178
CountryMexico	-0.702	0.192	-3.655	0.166	FA 20:5n-3 (EPA)	0.013359103
CountryMexico	-0.415	0.114	-3.654	0.221	LPC 18:1	0.013359103
CountryMexico	-0.246	0.067	-3.656	0.166	PG 16:0_20:4	0.013359103
CountryMexico	-0.592	0.162	-3.645	0.188	PE 30:0	0.013687122
CountryMexico	-0.354	0.097	-3.642	0.167	SM 36:1	0.013748118
CountryMexico	-0.625	0.175	-3.577	0.168	FA 20:3n-9	0.016803702
CountryMexico	-0.735	0.206	-3.565	0.161	FA 14:1n-5	0.017248456
CountryMexico	0.638	0.179	3.564	0.251	VLCAD Deficiency (NBS)	0.017248456
CountryMexico	-0.720	0.202	-3.560	0.176	3-IAA	0.017418926
CountryMexico	-0.309	0.087	-3.546	0.160	PC 36:4	0.018118459
CountryMexico	-1.228	0.347	-3.544	0.188	PE 34:0	0.018134033
CountryMexico	-0.403	0.114	-3.540	0.160	N-Ac-Met	0.01820223
CountryMexico	0.344	0.097	3.534	0.187	Uric Acid to Creatinine Ratio	0.018466998
CountryMexico	-0.464	0.132	-3.516	0.190	PG 20:4_22:4	0.019438871
CountryMexico	-0.601	0.172	-3.499	0.188	Cer d16:1/22:0	0.020373295
CountryMexico	-0.828	0.237	-3.493	0.170	CE 20:5	0.02062523
CountryMexico	-0.516	0.148	-3.482	0.201	LPC 20:3	0.021297211
CountryMexico	-0.310	0.089	-3.464	0.160	Malic acid	0.022391923
CountryMexico	-0.489	0.142	-3.452	0.158	FA 11:0	0.023147786
CountryMexico	-0.374	0.108	-3.448	0.168	PC 38:4	0.023327319
CountryMexico	-0.350	0.102	-3.442	0.178	PC O-44:4	0.023692629
CountryMexico	-0.397	0.115	-3.434	0.179	PG 16:2_18:1	0.024180578
CountryMexico	-0.354	0.103	-3.431	0.167	SM 33:1	0.024298122
CountryMexico	-0.211	0.062	-3.405	0.251	N-Ac-Asn	0.026215225

CountryMexico	-0.582	0.171	-3.397	0.219	CE 17:1	0.026719155
CountryMexico	-0.239	0.070	-3.396	0.161	Sum of SMs	0.026719155
CountryMexico	-0.300	0.089	-3.391	0.195	PC O-30:2	0.026818291
CountryMexico	-0.383	0.113	-3.394	0.271	Ribose	0.026818291
CountryMexico	-0.445	0.131	-3.385	0.177	N-Ac-Gln	0.02716437
CountryMexico	-0.264	0.078	-3.366	0.180	Maleic acid	0.028688578
CountryMexico	0.744	0.222	3.349	0.189	Asp Methylation	0.030142725
CountryMexico	-0.306	0.092	-3.339	0.259	Cit Synthesis	0.030635396
CountryMexico	0.306	0.092	3.339	0.259	OTC Deficiency (NBS)	0.030635396
CountryMexico	-0.230	0.069	-3.334	0.159	Sum of EC-FA SMs	0.031027974
CountryMexico	-1.019	0.306	-3.327	0.219	LPE P-17:0	0.031469371
CountryMexico	-0.255	0.077	-3.325	0.213	Sum of VLCFA-SMs	0.031469371
CountryMexico	-0.262	0.079	-3.317	0.159	PE 30:1	0.032098097
CountryMexico	-0.312	0.094	-3.309	0.210	SM 42:1	0.032550216
CountryMexico	-0.159	0.048	-3.309	0.238	Ratio of His to HCys+Phe+Sar	0.032550216
CountryMexico	-0.486	0.147	-3.305	0.174	Hypoxanthine	0.032639435
CountryMexico	-0.232	0.070	-3.298	0.149	Sum of UFA-PCs O	0.03316137
CountryMexico	-0.400	0.122	-3.263	0.140	Cer d18:1/20:0	0.036451251
CountryMexico	-0.316	0.097	-3.265	0.149	Sum of OC-FA SMs	0.036451251
CountryMexico	-0.231	0.071	-3.252	0.140	Sum of LCFA-SMs	0.037447885
CountryMexico	-1.095	0.337	-3.245	0.138	UDCA Synthesis from CDCA	0.038123927
CountryMexico	0.686	0.212	3.233	0.244	C12:1	0.039390813
CountryMexico	-0.310	0.096	-3.215	0.165	PC O-42:4	0.041178681
CountryMexico	-0.298	0.093	-3.210	0.133	PI 18:0_20:4	0.041690882
CountryMexico	0.534	0.166	3.208	0.230	GABA Synthesis	0.041807462
CountryMexico	-0.650	0.203	-3.200	0.173	3-Met-adipic acid	0.04243752
CountryMexico	-0.245	0.076	-3.201	0.284	Citrullinemia	0.04243752
CountryMexico	-0.550	0.173	-3.175	0.143	Hex-Cer d18:1/14:0	0.045381038
CountryMexico	-0.380	0.120	-3.172	0.140	PG 20:4_20:4	0.045602398
CountryMexico	-1.182	0.375	-3.154	0.136	Phenylacetylglutamine	0.047966853
CountryMexico	-0.427	0.135	-3.153	0.143	PE 35:1	0.047966853
CountryMexico	-0.629	0.200	-3.141	0.191	FA 5:0-3M	0.049255356
CountryMexico	-0.248	0.079	-3.137	0.139	Sum of PUFA-PCs O	0.049579085
CountryMexico	-0.245	0.079	-3.118	0.138	PC O-40:3	0.051565481
CountryMexico	-0.374	0.120	-3.120	0.123	PC O-44:5	0.051565481
CountryMexico	-0.666	0.215	-3.092	0.161	Quinaldic acid	0.055288358
CountryMexico	0.291	0.094	3.080	0.169	a-Ketoglutarate to Citrate Ratio	0.05665583
CountryMexico	-0.320	0.105	-3.066	0.142	PC 42:1	0.058534305
CountryMexico	-0.513	0.168	-3.056	0.133	CE 20:4	0.059419095
CountryMexico	-0.283	0.093	-3.052	0.166	PC 36:1	0.059883523
CountryMexico	0.472	0.156	3.034	0.274	HCit	0.062928557

CountryMexico	-0.335	0.111	-3.021	0.133	PC O-36:5	0.065230085
CountryMexico	-0.396	0.132	-3.010	0.177	PE P-16:0/18:1	0.066739606
CountryMexico	-0.523	0.175	-2.992	0.178	Cer d16:1/24:0	0.070108224
CountryMexico	-0.506	0.169	-2.987	0.119	Hex2Cer d18:1/18:0	0.071010092
CountryMexico	-0.402	0.136	-2.954	0.189	LPE 20:5	0.076739809
CountryMexico	-0.347	0.117	-2.956	0.141	LPE 22:5	0.076739809
CountryMexico	0.609	0.207	2.939	0.200	FA 5:0-4M	0.078865473
CountryMexico	0.734	0.250	2.936	0.163	C14:2	0.079103526
CountryMexico	-0.324	0.110	-2.937	0.132	LPC 24:0	0.079103526
CountryMexico	-0.623	0.213	-2.929	0.112	TG 20:4_36:4	0.08037427
CountryMexico	-0.734	0.251	-2.925	0.111	TG 18:0_38:6	0.081022178
CountryMexico	-0.346	0.118	-2.920	0.175	LPC 26:1	0.081999863
CountryMexico	-0.692	0.238	-2.905	0.143	PE P-18:0/20:5	0.085100007
CountryMexico	-0.303	0.105	-2.894	0.133	SM 41:2	0.086696016
CountryMexico	-0.263	0.091	-2.889	0.201	Hex2Cer d18:1/24:1	0.087792435
CountryMexico	-0.230	0.080	-2.879	0.171	SM 42:2	0.08964354
CountryMexico	-0.568	0.198	-2.875	0.142	2-OH-Isobutyric acid	0.090374294
CountryMexico	-0.357	0.125	-2.865	0.112	PC 42:0	0.092207633
CountryMexico	-0.238	0.084	-2.838	0.110	PC O-40:2	0.098299909
CountryMexico	-0.466	0.164	-2.837	0.174	PA 18:0_18:3	0.098319904
CountryMexico	-1.090	0.385	-2.828	0.126	Cinnamoyl-Gly	0.099806131
CountryMexico	-0.381	0.135	-2.827	0.126	PG 16:1_18:0	0.099806131
CountryMexico	-0.209	0.074	-2.826	0.116	SM 34:1	0.099806131
CountryMexico	-0.440	0.157	-2.810	0.142	PE P-18:1/20:4	0.103202262
CountryMexico	-0.274	0.098	-2.803	0.108	LPI 22:1	0.104836644
CountryMexico	-0.207	0.074	-2.788	0.155	Thr	0.108016596
CountryMexico	-0.106	0.038	-2.788	0.142	Sum of Non-Essential AAs	0.108016596
CountryMexico	-0.159	0.057	-2.780	0.134	GSH Constituents	0.109152854
CountryMexico	-0.561	0.202	-2.775	0.126	FA 20:4n-6 (AA)	0.11044385
CountryMexico	-0.197	0.071	-2.772	0.117	PG 18:1_20:0	0.111018107
CountryMexico	-0.264	0.096	-2.764	0.106	Sum of VLCFA-LPIs	0.112706714
CountryMexico	0.651	0.237	2.752	0.183	C14:1	0.115662643
CountryMexico	-0.250	0.091	-2.749	0.102	N-Ac-Val	0.115662643
CountryMexico	-0.370	0.134	-2.750	0.142	LPE 18:1	0.115662643
CountryMexico	-0.434	0.158	-2.750	0.109	PC O-38:2	0.115662643
CountryMexico	-0.102	0.037	-2.748	0.143	Sum of Solely Glucogenic AAs	0.115862544
CountryMexico	-0.517	0.188	-2.746	0.118	N2-Ac-Lys	0.115895196
CountryMexico	-0.238	0.087	-2.744	0.108	PC O-42:2	0.11631983
CountryMexico	-0.405	0.148	-2.740	0.118	Putrescine Synthesis	0.116945075
CountryMexico	0.468	0.171	2.735	0.215	Lys Carbamylation	0.118113224
CountryMexico	-0.776	0.284	-2.729	0.129	Cyclic Nucleotides Ratio	0.119093074

CountryMexico	-0.255	0.094	-2.723	0.156	Oxalic acid	0.120401096
CountryMexico	-0.285	0.105	-2.713	0.145	PG 16:0_16:1	0.122658953
CountryMexico	-0.244	0.090	-2.709	0.124	PC O-32:2	0.123291026
CountryMexico	-0.162	0.060	-2.705	0.152	N-Ac-Ala	0.124121311
CountryMexico	-0.554	0.206	-2.698	0.132	Cer d16:1/20:0	0.125785351
CountryMexico	-0.282	0.105	-2.697	0.130	PE 44:7	0.125785351
CountryMexico	-0.307	0.114	-2.683	0.113	SM 43:1	0.12976206
CountryMexico	-0.626	0.234	-2.678	0.149	LPE 17:1	0.130720386
CountryMexico	-0.221	0.082	-2.678	0.158	PA 18:0_18:1	0.130720386
CountryMexico	-0.815	0.305	-2.676	0.120	LPE 14:0	0.130874318
CountryMexico	-0.306	0.114	-2.675	0.110	PC O-44:3	0.130874318
CountryMexico	0.493	0.185	2.672	0.138	Benzoic acid	0.131537778
CountryMexico	-0.286	0.107	-2.666	0.107	SM 41:1	0.133288247
CountryMexico	-0.356	0.134	-2.657	0.122	LPC 16:1	0.13432796
CountryMexico	-0.456	0.172	-2.658	0.106	PE 33:0	0.13432796
CountryMexico	0.582	0.220	2.652	0.177	SPB d17:0	0.135656842
CountryMexico	-0.738	0.279	-2.646	0.162	TG 20:5_36:2	0.137344146
CountryMexico	-0.376	0.142	-2.642	0.092	FA 24:0	0.138407451
CountryMexico	-0.502	0.191	-2.630	0.173	Creatinine to Bilirubin Ratio	0.141525292
CountryMexico	-0.412	0.157	-2.621	0.125	SM 44:1	0.144074775
CountryMexico	-0.371	0.142	-2.619	0.163	LPC 14:0	0.14436372
CountryMexico	-0.248	0.095	-2.615	0.130	PC O-42:3	0.145506878
CountryMexico	-0.348	0.134	-2.605	0.135	N-Ac-His	0.148680365
CountryMexico	-0.232	0.089	-2.602	0.109	Creatinine	0.148992586
CountryMexico	-0.327	0.126	-2.600	0.110	PI 16:0_17:1	0.14904426
CountryMexico	-0.224	0.086	-2.594	0.102	Pyruvic acid	0.151070225
CountryMexico	-0.285	0.110	-2.592	0.107	PC 40:4	0.151299009
CountryMexico	-0.382	0.148	-2.584	0.112	PE 34:4	0.153497544
CountryMexico	-0.216	0.084	-2.585	0.179	PC 42:2	0.153497544
CountryMexico	-0.422	0.164	-2.573	0.099	PE 34:1	0.156763324
CountryMexico	-0.230	0.090	-2.571	0.109	Pyruvate to Glucose Ratio	0.15679762
CountryMexico	0.684	0.267	2.564	0.132	ACOD1 Activity	0.158021405
CountryMexico	-0.445	0.175	-2.546	0.111	PC 36:5	0.163375019
CountryMexico	-0.784	0.309	-2.536	0.087	PC O-38:1	0.165000513
CountryMexico	-0.391	0.154	-2.537	0.116	Serotonin Pathway Activity	0.165000513
CountryMexico	0.515	0.204	2.526	0.160	C12	0.167665947
CountryMexico	-0.327	0.130	-2.516	0.141	PE 38:0	0.171588018
CountryMexico	-0.257	0.103	-2.502	0.097	Sum of VLCFA-LPCs	0.176191849
CountryMexico	-0.250	0.101	-2.466	0.144	Choline	0.188570304
CountryMexico	-0.117	0.048	-2.456	0.094	Gln	0.191261662
CountryMexico	-1.323	0.540	-2.450	0.119	SPBP d18:1	0.19356034

CountryMexico	-0.237	0.097	-2.443	0.116	PC O-36:4	0.195753829
CountryMexico	-0.445	0.183	-2.434	0.178	KYNA DH	0.197931998
CountryMexico	-0.270	0.111	-2.431	0.147	PG 16:1_18:1	0.198520495
CountryMexico	-1.387	0.573	-2.422	0.124	SPBP d18:1 to Cer d18:1/24:1 Ratio	0.201927762
CountryMexico	-0.252	0.104	-2.420	0.123	beta-Ala	0.202438567
CountryMexico	-0.403	0.167	-2.417	0.115	CE 18:3	0.203470175
CountryMexico	-1.193	0.495	-2.409	0.080	TLCA Synthesis from CDCA	0.205890574
CountryMexico	-0.566	0.235	-2.408	0.088	PG 16:1_22:1	0.206161957
CountryMexico	-0.530	0.221	-2.402	0.137	LPS 18:1	0.208533965
CountryMexico	-0.266	0.111	-2.401	0.077	PG 20:4_22:1	0.208533965
CountryMexico	-0.171	0.072	-2.388	0.120	N6-Ac-Lys	0.212468615
CountryMexico	-0.218	0.092	-2.380	0.147	PC O-38:3	0.215710513
CountryMexico	-0.633	0.266	-2.379	0.089	TG 20:5_34:2	0.215934328
CountryMexico	-0.563	0.237	-2.375	0.132	THDCA	0.217260355
CountryMexico	0.279	0.118	2.373	0.126	PI 18:0_20:3	0.21795447
CountryMexico	-0.565	0.239	-2.364	0.151	Cer d18:0/22:0	0.221000389
CountryMexico	-0.350	0.148	-2.362	0.080	PE 36:5	0.221984548
CountryMexico	-0.388	0.164	-2.359	0.075	FA 14:0	0.22294524
CountryMexico	0.280	0.119	2.358	0.134	C18:1	0.2230741
CountryMexico	-0.262	0.112	-2.349	0.115	PE 44:6	0.226053848
CountryMexico	-0.253	0.108	-2.350	0.116	PC 40:2	0.226053848
CountryMexico	-0.494	0.211	-2.343	0.084	EMA (NBS)	0.227740132
CountryMexico	-0.674	0.288	-2.340	0.077	TG 20:5_36:3	0.228479796
CountryMexico	0.305	0.131	2.337	0.086	Phenylpyruvate to Citrate Ratio	0.229759165
CountryMexico	-0.251	0.108	-2.333	0.080	Arg	0.23136634
CountryMexico	-0.845	0.363	-2.330	0.180	Hex3Cer d18:1/22:0	0.232187416
CountryMexico	0.719	0.309	2.326	0.112	N1,N12-Di-Ac-Spermine	0.233732297
CountryMexico	-0.185	0.080	-2.312	0.108	PC O-34:1	0.239942948
CountryMexico	-0.513	0.223	-2.300	0.091	FA 4:0-3M	0.245022732
CountryMexico	0.478	0.208	2.299	0.198	TG 18:1_36:2	0.245022732
CountryMexico	-1.165	0.508	-2.294	0.102	SPBP d16:1	0.247792943
CountryMexico	-0.729	0.319	-2.288	0.147	Hex-Cer d16:1/20:0	0.250962813
CountryMexico	-0.363	0.159	-2.286	0.090	PE 38:3	0.251478949
CountryMexico	-0.343	0.150	-2.281	0.144	Cystine	0.253616715
CountryMexico	-0.371	0.163	-2.280	0.153	Cystine Synthesis	0.253845884
CountryMexico	-0.166	0.073	-2.278	0.093	Sum of PUFA-PC (O)s	0.254602188
CountryMexico	-1.106	0.487	-2.268	0.092	p-Cresol glucuronide	0.256300691
CountryMexico	-0.182	0.080	-2.268	0.098	Gly Synthesis	0.256300691
CountryMexico	-0.256	0.113	-2.260	0.094	PC 30:0	0.2600802
CountryMexico	-0.226	0.100	-2.256	0.106	PC 42:6	0.260901977
CountryMexico	0.296	0.131	2.252	0.105	Carnitine to Creatinine Ratio	0.261861322

CountryMexico	-0.207	0.092	-2.248	0.113	GABA	0.263160934
CountryMexico	-0.148	0.066	-2.246	0.101	TAT Activity	0.263387906
CountryMexico	-0.350	0.157	-2.234	0.192	3-Met-glutaric acid	0.26906513
CountryMexico	-0.421	0.190	-2.222	0.127	N-Met-Asp	0.271123817
CountryMexico	0.236	0.106	2.221	0.084	PI 18:2_22:1	0.271123817
CountryMexico	-0.457	0.206	-2.225	0.102	PC 24:0	0.271123817
CountryMexico	-0.219	0.098	-2.226	0.090	PC 42:4	0.271123817
CountryMexico	-0.468	0.211	-2.218	0.131	Imidazolepropionic acid	0.271822195
CountryMexico	-0.447	0.202	-2.217	0.142	Cer d18:2/18:0	0.272122345
CountryMexico	-0.515	0.234	-2.206	0.158	Cer d16:1/23:0	0.276925993
CountryMexico	-0.230	0.104	-2.199	0.073	Hex-Cer d18:1/24:1	0.27973733
CountryMexico	-0.264	0.120	-2.196	0.074	PI 18:1_20:4	0.280207784
CountryMexico	0.130	0.059	2.193	0.121	LPI 16:0	0.280936507
CountryMexico	-0.199	0.091	-2.189	0.071	PS 36:4	0.282418394
CountryMexico	-0.589	0.269	-2.188	0.136	TG 22:3_30:2	0.282418394
CountryMexico	-0.392	0.181	-2.169	0.093	LPE 18:3	0.292068466
CountryMexico	-0.725	0.336	-2.156	0.079	Met-IAA	0.29794482
CountryMexico	-0.234	0.109	-2.146	0.073	SM 36:2	0.303004764
CountryMexico	0.422	0.197	2.141	0.154	Argininic acid	0.305513472
CountryMexico	0.411	0.192	2.138	0.065	Protection against MDD	0.306211215
CountryMexico	-0.233	0.109	-2.128	0.068	Hex-Cer d18:1/16:0	0.31234382
CountryMexico	-0.246	0.116	-2.118	0.120	Guanidinoacetic acid	0.317484346
CountryMexico	0.229	0.108	2.116	0.136	Betaine	0.317903377
CountryMexico	0.358	0.170	2.106	0.111	PA 17:1_18:2	0.322153345
CountryMexico	0.350	0.167	2.100	0.165	C16:1	0.32254172
CountryMexico	-0.588	0.280	-2.098	0.143	Adipic acid	0.32254172
CountryMexico	-0.180	0.086	-2.102	0.060	PC O-38:5	0.32254172
CountryMexico	-0.135	0.064	-2.099	0.146	PG 16:0_19:1	0.32254172
CountryMexico	0.155	0.074	2.093	0.102	MRC Disorders	0.324581265
CountryMexico	0.408	0.195	2.089	0.203	DG 18:1_18:1	0.324701793
CountryMexico	0.374	0.180	2.086	0.139	PA 17:1_18:1	0.325067814
CountryMexico	1.051	0.504	2.086	0.090	TG 20:0_34:1	0.325067814
CountryMexico	0.799	0.384	2.081	0.118	DG 16:1_18:1	0.327158618
CountryMexico	-0.237	0.114	-2.081	0.059	Met Oxidation	0.327158618
CountryMexico	-0.336	0.162	-2.077	0.083	N-Ac-Leu	0.328295425
CountryMexico	-0.174	0.084	-2.067	0.059	Butyric Acid to Isobutyric Acid Ratio	0.333773018
CountryMexico	0.285	0.138	2.066	0.070	Cortisone	0.333898916
CountryMexico	-0.822	0.399	-2.063	0.064	SPB d16:0	0.334809156
CountryMexico	0.217	0.105	2.061	0.076	Asymmetrical Arg Methylation	0.334809156
CountryMexico	-0.132	0.064	-2.060	0.246	Ratio of PUFA-LPCs to MUFA-LPCs	0.334809156
CountryMexico	-0.185	0.090	-2.057	0.111	PC 40:3	0.335712429

CountryMexico	-0.495	0.241	-2.052	0.082	TG 20:4_34:3	0.338515365
CountryMexico	-0.266	0.130	-2.044	0.103	LPE 22:6	0.341999224
CountryMexico	0.166	0.082	2.040	0.135	PG 18:1_22:5	0.344228053
CountryMexico	0.681	0.337	2.023	0.155	C14:1-OH	0.354990132
CountryMexico	-0.077	0.038	-2.022	0.099	Sum of AAs	0.355253984
CountryMexico	-0.227	0.113	-2.013	0.063	Cer d18:1/18:0	0.359857465
CountryMexico	-0.134	0.067	-1.998	0.070	PG 16:0_20:3	0.368588728
CountryMexico	-0.420	0.210	-1.996	0.070	TG 20:4_34:2	0.369504941
CountryMexico	-0.171	0.086	-1.994	0.118	LPI 18:0	0.370785157
CountryMexico	-0.686	0.345	-1.992	0.126	TG 20:4_33:2	0.371789294
CountryMexico	-0.152	0.076	-1.991	0.090	Sum of PUFA-PCs	0.371789294
CountryMexico	-0.340	0.171	-1.982	0.066	Methylmalonic acid	0.37535574
CountryMexico	-0.424	0.215	-1.975	0.092	FA 10:0	0.37945946
CountryMexico	0.260	0.132	1.969	0.067	PI 18:1_18:2	0.380924964
CountryMexico	0.209	0.107	1.955	0.166	SCD-1 index	0.387974646
CountryMexico	0.417	0.214	1.947	0.131	PA 17:2_18:1	0.391191077
CountryMexico	-0.197	0.101	-1.946	0.164	NOS activity	0.391383284
CountryMexico	-0.591	0.304	-1.944	0.055	TLCA	0.39248105
CountryMexico	-0.091	0.047	-1.943	0.054	Ratio of Non-Essential to Essential AAs	0.39248105
CountryMexico	-0.234	0.121	-1.932	0.091	PA 18:2_20:2	0.398795828
CountryMexico	-0.210	0.109	-1.928	0.067	PC O-40:6	0.40123095
CountryMexico	-0.361	0.187	-1.927	0.053	TG 20:4_36:3	0.40139247
CountryMexico	0.221	0.115	1.926	0.115	PA 18:2_22:1	0.401649432
CountryMexico	-0.193	0.101	-1.913	0.066	Hex3Cer d18:1/16:0	0.408929815
CountryMexico	-0.395	0.207	-1.903	0.083	BABA	0.414574407
CountryMexico	-0.142	0.075	-1.900	0.120	SDMA	0.416511348
CountryMexico	-0.607	0.319	-1.900	0.088	Phenylacetic acid	0.416511348
CountryMexico	-0.511	0.270	-1.894	0.097	LPS 20:5	0.420127059
CountryMexico	-0.124	0.066	-1.892	0.191	N-Ac-Asp	0.421358496
CountryMexico	-0.440	0.234	-1.883	0.091	PE P-18:1/20:5	0.425307374
CountryMexico	-0.394	0.209	-1.882	0.143	Imidazolepropionic Acid Synthesis	0.425307374
CountryMexico	-0.446	0.238	-1.877	0.054	TG 18:1_38:7	0.427248556
CountryMexico	0.143	0.077	1.867	0.078	BCAT Leu Catabolism	0.432428856
CountryMexico	-0.142	0.076	-1.867	0.047	Orn	0.432429327
CountryMexico	-0.225	0.120	-1.866	0.149	5-HIAA	0.432429327
CountryMexico	-0.165	0.088	-1.859	0.049	Gly	0.43513897
CountryMexico	-0.275	0.148	-1.857	0.067	DG 17:0_18:1	0.435283945
CountryMexico	-0.225	0.121	-1.856	0.066	Hex-Cer d18:1/24:0	0.435298039
CountryMexico	0.243	0.131	1.855	0.174	LPI 14:1	0.435326371
CountryMexico	-0.259	0.140	-1.854	0.078	PI 18:0_18:3	0.435330836
CountryMexico	0.286	0.154	1.854	0.173	PG 15:0_18:1	0.435330836

CountryMexico	-0.175	0.095	-1.850	0.065	FA 7:0	0.437091041
CountryMexico	-0.334	0.181	-1.844	0.056	FA 15:0	0.438595627
CountryMexico	-0.118	0.064	-1.837	0.100	PE 40:3	0.442316543
CountryMexico	0.304	0.166	1.836	0.122	AABA Synthesis	0.442500939
CountryMexico	0.135	0.073	1.835	0.165	a-Ketoglutaric acid	0.442616694
CountryMexico	-0.156	0.085	-1.830	0.094	Citric acid	0.444369342
CountryMexico	-0.309	0.169	-1.830	0.102	TG 20:4_36:2	0.444369342
CountryMexico	-0.524	0.287	-1.830	0.118	TG 20:5_34:1	0.444369342
CountryMexico	-0.724	0.397	-1.823	0.125	TG 20:4_30:0	0.448196234
CountryMexico	0.260	0.143	1.819	0.123	PA 18:0_18:2	0.449621157
CountryMexico	-0.321	0.176	-1.818	0.103	Sum MCFA	0.449621157
CountryMexico	-0.185	0.103	-1.804	0.095	PG 18:1_22:3	0.458659157
CountryMexico	-0.143	0.080	-1.793	0.089	FA 9:0	0.46546037
CountryMexico	-0.388	0.217	-1.789	0.060	Mevalonic acid	0.467581809
CountryMexico	-0.232	0.130	-1.787	0.063	PE 44:12	0.469332345
CountryMexico	-0.346	0.195	-1.777	0.056	FA 22:5n-3 (DPA)	0.471177455
CountryMexico	-0.307	0.173	-1.778	0.089	PE 40:6	0.471177455
CountryMexico	-0.130	0.073	-1.775	0.057	Cer d18:1/16:0	0.47138219
CountryMexico	-0.182	0.103	-1.762	0.052	Cer d18:1/22:0	0.472675477
CountryMexico	-0.256	0.145	-1.766	0.099	PE 33:1	0.472675477
CountryMexico	-0.117	0.066	-1.762	0.092	Ratio of SGA to Glucose	0.472675477
CountryMexico	0.088	0.050	1.754	0.063	MTHFR Deficiency (NBS)	0.476925238
CountryMexico	-0.183	0.105	-1.750	0.064	Hex-Cer d18:1/18:0	0.477860014
CountryMexico	-0.183	0.105	-1.745	0.059	C4:1	0.478424234
CountryMexico	-0.189	0.109	-1.745	0.084	1-Met-His	0.478424234
CountryMexico	-0.144	0.082	-1.744	0.060	SM 34:2	0.478424234
CountryMexico	-0.248	0.143	-1.734	0.058	PE 36:6	0.485320961
CountryMexico	0.390	0.225	1.734	0.189	TG 18:1_34:1	0.485320961
CountryMexico	-0.387	0.223	-1.733	0.055	TG 16:0_38:6	0.485434657
CountryMexico	-0.214	0.124	-1.730	0.068	Sum of PUFA-CEs	0.485991877
CountryMexico	0.510	0.295	1.727	0.137	TG 16:0_38:2	0.487451843
CountryMexico	-0.133	0.077	-1.724	0.063	HSer	0.48834065
CountryMexico	0.149	0.087	1.723	0.110	Phenylpyruvic acid	0.489060918
CountryMexico	-0.405	0.235	-1.721	0.102	PE P-16:0/20:5	0.489060918
CountryMexico	0.740	0.430	1.718	0.070	Gly Conjugation of CA	0.491360269
CountryMexico	-0.279	0.163	-1.714	0.057	Indole-Lac	0.493997658
CountryMexico	-0.933	0.545	-1.712	0.051	p-Cresol-SO4 Synthesis	0.495691031
CountryMexico	-0.158	0.093	-1.704	0.081	PC 42:5	0.498645241
CountryMexico	-0.472	0.278	-1.698	0.043	SM 44:2	0.50291887
CountryMexico	-0.236	0.139	-1.695	0.096	PE P-18:0/22:6	0.505494449
CountryMexico	-0.174	0.103	-1.693	0.087	PC 38:5	0.506331118

CountryMexico	-0.404	0.239	-1.691	0.109	Suberic acid	0.506819519
CountryMexico	-0.541	0.321	-1.688	0.068	Hex3Cer d18:1/18:0	0.507524668
CountryMexico	0.228	0.136	1.679	0.098	DMG	0.509884198
CountryMexico	-0.098	0.058	-1.677	0.069	Asn	0.509884198
CountryMexico	-0.152	0.091	-1.673	0.096	Urea	0.509884198
CountryMexico	0.178	0.106	1.674	0.100	T4	0.509884198
CountryMexico	0.183	0.109	1.678	0.142	PG 16:3_18:1	0.509884198
CountryMexico	-0.385	0.230	-1.677	0.040	TG 18:2_38:6	0.509884198
CountryMexico	-0.476	0.283	-1.681	0.061	TG 20:3_34:3	0.509884198
CountryMexico	-0.271	0.163	-1.666	0.064	cGMP	0.512020455
CountryMexico	-0.201	0.121	-1.663	0.040	PI 16:1_18:0	0.513220124
CountryMexico	-0.144	0.087	-1.661	0.112	Sum of MUFA-PGs	0.513889221
CountryMexico	0.543	0.328	1.656	0.093	C16:2	0.514137204
CountryMexico	0.254	0.154	1.653	0.155	PA 16:1_18:1	0.514876218
CountryMexico	-0.433	0.262	-1.654	0.077	TG 18:2_36:1	0.514876218
CountryMexico	-0.557	0.337	-1.652	0.106	TG 20:3_32:2	0.514876218
CountryMexico	-0.448	0.271	-1.651	0.099	TG 20:4_32:2	0.514876218
CountryMexico	-0.208	0.126	-1.648	0.049	PC O-38:0	0.516178366
CountryMexico	-0.094	0.057	-1.648	0.101	Sum of Dimethylated Arg	0.516207195
CountryMexico	-0.322	0.196	-1.647	0.062	LPE 15:0	0.516794868
CountryMexico	0.572	0.349	1.639	0.151	TG 20:1_34:3	0.520958247
CountryMexico	0.300	0.184	1.628	0.096	PE P-16:0/18:2	0.526181895
CountryMexico	0.485	0.298	1.629	0.144	TG 16:1_38:4	0.526181895
CountryMexico	0.100	0.062	1.620	0.072	PKU (NBS)	0.530377577
CountryMexico	0.188	0.116	1.619	0.073	PI 18:0_18:2	0.531605649
CountryMexico	-0.573	0.355	-1.616	0.061	IsoUDCA	0.531808872
CountryMexico	-0.329	0.204	-1.617	0.087	LPE P-22:0	0.531808872
CountryMexico	-0.477	0.295	-1.614	0.090	TG 20:4_32:1	0.532789484
CountryMexico	0.101	0.063	1.609	0.086	PC 34:2	0.535583388
CountryMexico	-0.819	0.514	-1.595	0.045	Total p-Cresol Derivatives	0.544772388
CountryMexico	-0.823	0.516	-1.593	0.045	p-Cresol-SO4	0.544806474
CountryMexico	-0.319	0.201	-1.590	0.054	FA 12:0	0.54503181
CountryMexico	-0.295	0.185	-1.592	0.062	FA 22:4n-6	0.54503181
CountryMexico	-0.264	0.166	-1.589	0.090	Hex-Cer d18:2/16:0	0.54503181
CountryMexico	-0.086	0.055	-1.582	0.095	Ratio of OC-FA SMs to EC-FA SMs	0.548018874
CountryMexico	0.240	0.152	1.580	0.064	PA 17:0_18:2	0.548820569
CountryMexico	-0.157	0.100	-1.580	0.117	PI 17:1_18:1	0.548820569
CountryMexico	-0.465	0.295	-1.574	0.041	TG 18:3_38:5	0.552034924
CountryMexico	-0.211	0.134	-1.574	0.176	Pantothenic acid (B5)	0.552034924
CountryMexico	-0.181	0.115	-1.574	0.050	Acetic Acid to Isobutyric Acid Ratio	0.552034924
CountryMexico	-0.877	0.558	-1.571	0.090	DG 18:2_18:4	0.552485032

CountryMexico	-0.074	0.047	-1.568	0.101	His	0.553838794
CountryMexico	-0.621	0.396	-1.569	0.064	TG 20:4_34:0	0.553838794
CountryMexico	0.139	0.089	1.565	0.054	Phenylpyruvate Synthesis	0.556883974
CountryMexico	0.217	0.140	1.557	0.098	PA 18:1_18:1	0.562131883
CountryMexico	-0.160	0.103	-1.558	0.088	LPG 18:0	0.562131883
CountryMexico	0.241	0.155	1.554	0.111	PA 17:0_18:1	0.563478323
CountryMexico	-0.348	0.224	-1.551	0.064	GCDH Deficiency	0.56494543
CountryMexico	-0.384	0.248	-1.548	0.066	Desamino-Tyr	0.566969333
CountryMexico	-0.273	0.178	-1.536	0.117	CE 16:1	0.574518482
CountryMexico	-0.224	0.146	-1.538	0.078	PC 34:4	0.574518482
CountryMexico	0.118	0.077	1.529	0.058	FA 3:0-2M	0.577618839
CountryMexico	-0.866	0.569	-1.523	0.075	3-EpiDCA	0.58088135
CountryMexico	0.311	0.204	1.523	0.075	PE P-16:0/20:3	0.58088135
CountryMexico	0.138	0.091	1.519	0.067	PC 36:3	0.582728943
CountryMexico	-0.174	0.114	-1.520	0.051	PC O-38:6	0.582728943
CountryMexico	-0.250	0.165	-1.516	0.101	PG 16:1_16:1	0.582728943
CountryMexico	0.326	0.215	1.516	0.161	TG 16:0_36:2	0.582728943
CountryMexico	0.272	0.180	1.513	0.117	TG 18:2_36:2	0.58359756
CountryMexico	0.219	0.145	1.508	0.037	PI 18:1_18:1	0.585318043
CountryMexico	0.354	0.236	1.503	0.215	TG 16:1_36:2	0.588193853
CountryMexico	-0.313	0.209	-1.501	0.094	TG 20:4_34:1	0.589780816
CountryMexico	-0.110	0.073	-1.499	0.077	Sum of SphoPs	0.590112266
CountryMexico	-0.401	0.269	-1.494	0.075	TG 20:4_32:0	0.592061225
CountryMexico	0.098	0.066	1.494	0.066	Ratio of MUFA-LPCs to SFA-LPCs	0.592061225
CountryMexico	-0.128	0.086	-1.493	0.049	PC O-36:2	0.592675102
CountryMexico	0.347	0.233	1.491	0.082	c4-OH-Pro	0.59388093
CountryMexico	0.133	0.090	1.489	0.073	Brain Trp Availability	0.595076706
CountryMexico	0.167	0.112	1.487	0.051	Trp	0.596251964
CountryMexico	-0.585	0.394	-1.487	0.046	TG 20:4_36:5	0.596251964
CountryMexico	-0.144	0.097	-1.482	0.086	Hex2Cer d18:1/16:0	0.599049007
CountryMexico	0.112	0.076	1.477	0.060	Uric acid	0.600852354
CountryMexico	0.362	0.245	1.474	0.065	TG 20:0_32:3	0.602663432
CountryMexico	-0.156	0.106	-1.471	0.046	PI 18:2_20:4	0.60424403
CountryMexico	0.236	0.161	1.466	0.074	C4	0.60757359
CountryMexico	-0.237	0.162	-1.466	0.134	5-HIAA / Quinolinic Acid Ratio	0.60757359
CountryMexico	-0.360	0.246	-1.464	0.070	Arginino-Suc	0.608220485
CountryMexico	0.098	0.067	1.455	0.055	Met	0.6086916
CountryMexico	-0.455	0.312	-1.457	0.141	CE 14:0	0.6086916
CountryMexico	-0.103	0.071	-1.456	0.129	PA 18:3_18:3	0.6086916
CountryMexico	-0.140	0.096	-1.457	0.082	PC O-28:0	0.6086916
CountryMexico	-0.187	0.128	-1.456	0.057	Picolinic Acid to Trp Ratio	0.6086916

CountryMexico	-0.227	0.157	-1.448	0.091	LPE 18:2	0.611656345
CountryMexico	-0.190	0.132	-1.447	0.105	Hex-Cer d18:1/18:1	0.611799407
CountryMexico	-0.347	0.240	-1.444	0.077	SPHK activity (8)	0.611832799
CountryMexico	-0.386	0.267	-1.443	0.047	Cer d16:1/18:0	0.612808528
CountryMexico	-0.157	0.109	-1.441	0.103	CE 16:0	0.614126863
CountryMexico	0.110	0.077	1.441	0.033	Tyr	0.614226121
CountryMexico	0.263	0.183	1.439	0.115	TG 18:1_36:3	0.614226121
CountryMexico	-0.164	0.115	-1.423	0.066	LPI 20:1	0.619637508
CountryMexico	-0.147	0.103	-1.424	0.035	PG 18:1_20:1	0.619637508
CountryMexico	0.092	0.065	1.419	0.047	Sum of Aromatic AAs	0.621264515
CountryMexico	-0.069	0.049	-1.414	0.080	Fischer Ratio	0.62415714
CountryMexico	0.192	0.136	1.413	0.036	C18:2	0.62426111
CountryMexico	-0.202	0.143	-1.409	0.098	Z-3-Met-glutaconic acid	0.625881202
CountryMexico	0.270	0.191	1.408	0.121	Bilirubin	0.625881202
CountryMexico	-0.189	0.135	-1.408	0.080	PE 36:0	0.625881202
CountryMexico	-0.612	0.437	-1.402	0.156	CA	0.627793048
CountryMexico	-0.141	0.101	-1.396	0.178	N8-Ac-Spermidine	0.631895514
CountryMexico	-0.146	0.104	-1.397	0.064	2MBG (NBS)	0.631895514
CountryMexico	0.302	0.217	1.393	0.092	C10:2	0.633207468
CountryMexico	-0.182	0.131	-1.391	0.033	HCys Synthesis	0.633878322
CountryMexico	-0.175	0.126	-1.386	0.092	PE P-16:0/22:6	0.636062823
CountryMexico	-0.177	0.129	-1.376	0.050	Hex-Cer d18:1/23:0	0.641957342
CountryMexico	-0.657	0.477	-1.376	0.026	GLCA Synthesis from CDCA	0.641998079
CountryMexico	-0.216	0.157	-1.373	0.081	LPE 20:3	0.644250121
CountryMexico	-0.149	0.108	-1.372	0.043	Secondary 3MG Aciduria	0.644250121
CountryMexico	-0.514	0.375	-1.370	0.068	TG 18:3_38:6	0.644819909
CountryMexico	-0.255	0.187	-1.369	0.082	MCFA PPAR Modulators	0.644819909
CountryMexico	0.181	0.133	1.359	0.056	alpha-AAA	0.650670635
CountryMexico	-0.153	0.112	-1.358	0.056	FA 18:0	0.650670635
CountryMexico	-0.515	0.379	-1.358	0.037	PC 36:0	0.650670635
CountryMexico	-0.278	0.204	-1.362	0.029	TG 18:0_36:4	0.650670635
CountryMexico	0.296	0.218	1.358	0.094	TG 20:1_34:2	0.650670635
CountryMexico	0.145	0.107	1.357	0.035	4-Met-2-oxovaleric acid	0.651770479
CountryMexico	-0.158	0.117	-1.354	0.064	Hex-Cer d18:1/22:0	0.651802516
CountryMexico	0.319	0.235	1.355	0.029	3,5-DiOH-Benzoic acid	0.651802516
CountryMexico	0.282	0.208	1.355	0.082	TG 16:0_38:3	0.651802516
CountryMexico	-0.153	0.113	-1.352	0.045	Ratio of MUFA-PGs to SFA-PGs	0.652798468
CountryMexico	-0.149	0.111	-1.344	0.038	LPG 18:2	0.656075836
CountryMexico	0.157	0.117	1.344	0.046	Sum of Asym. and Sym. Arg Methylation	0.656075836
CountryMexico	-0.148	0.111	-1.336	0.080	IDO Activity	0.66232806
CountryMexico	-0.121	0.091	-1.334	0.072	Ratio of SG to Glucose	0.662629197

CountryMexico	-0.391	0.294	-1.330	0.038	Ind-SO4	0.665831465
CountryMexico	0.092	0.069	1.324	0.141	Glyoxylic acid	0.670191792
CountryMexico	-0.167	0.126	-1.320	0.071	CE 18:1	0.67030417
CountryMexico	0.183	0.138	1.322	0.076	PI 16:0_18:2	0.67030417
CountryMexico	-0.528	0.399	-1.321	0.047	TG 18:0_32:0	0.67030417
CountryMexico	-0.239	0.181	-1.322	0.032	KAT A Activity	0.67030417
CountryMexico	-0.284	0.216	-1.316	0.038	PG 18:1_20:2	0.672065727
CountryMexico	0.251	0.192	1.308	0.119	DG 18:1_18:2	0.675787164
CountryMexico	-0.302	0.231	-1.307	0.069	TG 16:0_40:6	0.675787164
CountryMexico	-0.430	0.330	-1.303	0.061	TG 17:0_32:1	0.675819157
CountryMexico	-0.589	0.452	-1.304	0.027	Gly Conjugation of CDCA	0.675819157
CountryMexico	-0.218	0.168	-1.300	0.104	PG 17:1_18:1	0.676619533
CountryMexico	-0.493	0.380	-1.297	0.041	UDCA	0.676872741
CountryMexico	-0.167	0.129	-1.295	0.084	Hex-Cer d18:2/22:0	0.677094997
CountryMexico	0.602	0.469	1.285	0.035	CDCA	0.681552609
CountryMexico	0.217	0.169	1.284	0.170	N-Ac-Putrescine to N1- and N8-Ac-Spermidine Ratio	0.681552609
CountryMexico	0.139	0.108	1.278	0.088	PI 18:1_22:1	0.683205039
CountryMexico	-0.236	0.185	-1.274	0.073	Glutaric acid	0.685703126
CountryMexico	-0.100	0.079	-1.271	0.139	PI 16:1_18:1	0.686727662
CountryMexico	-0.102	0.081	-1.268	0.172	Ala	0.688271753
CountryMexico	0.185	0.146	1.266	0.026	Cortisol	0.688688089
CountryMexico	0.346	0.273	1.267	0.097	BABA Synthesis	0.688688089
CountryMexico	-0.419	0.332	-1.264	0.045	TG 16:0_38:7	0.689378866
CountryMexico	-0.573	0.453	-1.264	0.037	Taurine Conjugation of CDCA	0.689378866
CountryMexico	0.098	0.078	1.261	0.111	PG 18:2_22:0	0.690878506
CountryMexico	-0.885	0.704	-1.257	0.077	2,5-Furandicarboxylic acid	0.690901009
CountryMexico	-0.528	0.421	-1.255	0.032	TDCA	0.691612288
CountryMexico	-0.138	0.110	-1.254	0.039	PI 18:1_22:6	0.691807634
CountryMexico	-0.210	0.168	-1.254	0.072	PC 36:6	0.691807634
CountryMexico	-0.123	0.098	-1.246	0.070	PG 16:0_20:5	0.696243556
CountryMexico	-0.355	0.286	-1.242	0.113	LPE P-22:1	0.698071933
CountryMexico	-0.289	0.234	-1.236	0.087	PE P-18:0/18:3	0.700948011
CountryMexico	-0.156	0.127	-1.232	0.061	PI 16:0_20:4	0.701559351
CountryMexico	-0.131	0.106	-1.232	0.027	PS 38:6	0.701559351
CountryMexico	-0.325	0.264	-1.231	0.082	TG 20:3_32:0	0.701559351
CountryMexico	-0.186	0.151	-1.230	0.056	PE 35:2	0.702233989
CountryMexico	0.103	0.084	1.224	0.072	PG 18:2_20:3	0.706559354
CountryMexico	-0.233	0.191	-1.221	0.044	TG 17:0_36:4	0.70792728
CountryMexico	-0.182	0.150	-1.216	0.102	CE 20:3	0.709804529
CountryMexico	0.227	0.188	1.207	0.116	TG 18:1_34:2	0.717056776
CountryMexico	0.077	0.064	1.200	0.092	PG 16:0_18:3	0.71897343

CountryMexico	-0.222	0.186	-1.194	0.028	N-Ac-Tyr	0.723648112
CountryMexico	-0.149	0.126	-1.189	0.040	LPC 26:0	0.725275887
CountryMexico	0.137	0.115	1.188	0.032	PC 34:3	0.725275887
CountryMexico	-0.265	0.223	-1.189	0.063	TG 18:2_31:0	0.725275887
CountryMexico	-0.245	0.206	-1.187	0.110	TG 20:3_34:2	0.726702784
CountryMexico	0.277	0.234	1.186	0.093	Ethylmalonic acid	0.726901406
CountryMexico	0.233	0.198	1.182	0.026	Ratio Quinolinic Acid to KYNA	0.730673005
CountryMexico	-0.231	0.196	-1.180	0.049	LPE 20:2	0.732068194
CountryMexico	-0.503	0.426	-1.179	0.047	TG 14:0_35:2	0.732068194
CountryMexico	0.238	0.203	1.171	0.075	TG 18:3_36:2	0.734659259
CountryMexico	-0.245	0.210	-1.170	0.125	Hex-Cer d18:1/26:1	0.735495638
CountryMexico	-0.181	0.156	-1.165	0.124	GAA to HArg Ratio	0.737696495
CountryMexico	-0.139	0.119	-1.161	0.028	Met-SO	0.738515723
CountryMexico	0.265	0.228	1.161	0.084	PI 17:0_18:1	0.738515723
CountryMexico	-0.253	0.219	-1.158	0.041	Malonic acid	0.739863384
CountryMexico	0.231	0.202	1.144	0.095	DG 16:0_18:1	0.747947347
CountryMexico	-0.426	0.374	-1.141	0.083	TG 17:1_32:1	0.749595167
CountryMexico	-0.129	0.114	-1.137	0.033	C18	0.751252254
CountryMexico	-0.163	0.144	-1.134	0.054	PE P-18:0/18:1	0.751252254
CountryMexico	-0.300	0.264	-1.136	0.071	TG 22:4_34:2	0.751252254
CountryMexico	-0.141	0.124	-1.133	0.088	Hex-Cer d18:2/24:0	0.75148364
CountryMexico	-0.138	0.122	-1.129	0.048	CE 18:2	0.752806895
CountryMexico	0.266	0.236	1.128	0.123	Anthranilic acid	0.752847723
CountryMexico	0.177	0.157	1.127	0.068	PA 16:1_18:2	0.753042455
CountryMexico	-0.221	0.197	-1.121	0.046	Kynurenic acid	0.755264704
CountryMexico	-0.203	0.181	-1.119	0.153	E-3-Met-glutaconic acid	0.756364037
CountryMexico	0.089	0.079	1.116	0.137	PA 16:0_19:2	0.758060999
CountryMexico	0.287	0.257	1.116	0.053	C18:1-OH	0.758569803
CountryMexico	0.104	0.094	1.109	0.058	PG 18:1_18:3	0.761693161
CountryMexico	0.104	0.094	1.108	0.025	Sum of BC-a-ketoacids	0.762246381
CountryMexico	-0.504	0.456	-1.107	0.025	TG 16:1_28:0	0.762811269
CountryMexico	0.277	0.251	1.103	0.023	HMGCR Activity	0.762811269
CountryMexico	-0.249	0.226	-1.100	0.027	N-Ac-Gly	0.763652624
CountryMexico	-0.276	0.253	-1.093	0.019	N-Ac-Ile	0.763652624
CountryMexico	-0.579	0.530	-1.094	0.022	GUDCA	0.763652624
CountryMexico	-0.390	0.356	-1.094	0.044	NorDCA	0.763652624
CountryMexico	0.079	0.072	1.094	0.045	FA 6:0	0.763652624
CountryMexico	-0.359	0.328	-1.094	0.062	LPE 19:0	0.763652624
CountryMexico	-0.318	0.291	-1.093	0.061	Xanthine	0.763652624
CountryMexico	-0.130	0.119	-1.091	0.019	PC 38:0	0.764021009
CountryMexico	-0.115	0.106	-1.091	0.098	1-Met-His Synthesis	0.764021009

CountryMexico	-0.194	0.179	-1.082	0.030	Citramalic acid	0.769696796
CountryMexico	-0.281	0.260	-1.081	0.019	TG 20:3_36:4	0.770515651
CountryMexico	0.547	0.506	1.080	0.049	Taurine Conjugation of CA	0.770857275
CountryMexico	-0.332	0.308	-1.075	0.089	TG 20:2_34:3	0.772981038
CountryMexico	0.136	0.127	1.066	0.108	PA 16:0_18:1	0.7734465
CountryMexico	0.208	0.195	1.068	0.059	PA 18:1_18:3	0.7734465
CountryMexico	0.223	0.208	1.070	0.157	TG 16:1_36:3	0.7734465
CountryMexico	0.079	0.074	1.066	0.054	PLA activity (2)	0.7734465
CountryMexico	0.171	0.161	1.061	0.056	PI 18:1_20:2	0.776133059
CountryMexico	0.073	0.069	1.062	0.021	BCAT Ile Catabolism	0.776133059
CountryMexico	-0.368	0.348	-1.058	0.042	TUDCA	0.778530373
CountryMexico	0.136	0.129	1.053	0.103	PI 16:0_20:3	0.7803233
CountryMexico	-0.120	0.115	-1.049	0.023	PE 32:2	0.78317616
CountryMexico	0.241	0.230	1.046	0.154	TG 20:1_34:1	0.784490321
CountryMexico	0.146	0.140	1.043	0.177	N1,N8-Di-Ac-Spermidine	0.784816986
CountryMexico	-0.439	0.420	-1.044	0.071	TG 14:0_35:1	0.784816986
CountryMexico	-0.465	0.446	-1.044	0.103	TG 22:4_32:0	0.784816986
CountryMexico	-0.761	0.731	-1.041	0.075	MG 22:4	0.786541942
CountryMexico	-0.432	0.418	-1.033	0.093	Trigonelline	0.789010564
CountryMexico	-0.351	0.340	-1.033	0.100	7-KetoDCA	0.789010564
CountryMexico	0.113	0.109	1.033	0.068	PG 18:2_18:4	0.789010564
CountryMexico	0.088	0.085	1.035	0.066	PG 18:2_20:4	0.789010564
CountryMexico	-0.195	0.189	-1.030	0.076	Hex-Cer d18:2/18:0	0.789447768
CountryMexico	0.224	0.217	1.030	0.059	LPI 18:3	0.789447768
CountryMexico	0.170	0.166	1.027	0.085	DG 18:1_20:1	0.789904956
CountryMexico	-0.516	0.507	-1.019	0.034	GDCA	0.792854484
CountryMexico	0.248	0.243	1.020	0.142	Kynureninase A Activity	0.792854484
CountryMexico	0.169	0.166	1.017	0.053	PE P-18:1/18:2	0.793046292
CountryMexico	-0.256	0.252	-1.017	0.068	TG 16:0_33:2	0.793046292
CountryMexico	0.144	0.142	1.014	0.044	PI 16:0_18:1	0.793175569
CountryMexico	0.084	0.083	1.015	0.148	PG 16:0_22:2	0.793175569
CountryMexico	-0.509	0.502	-1.013	0.021	PC O-30:1	0.793525266
CountryMexico	-0.104	0.103	-1.012	0.079	PC 40:5	0.793644287
CountryMexico	-0.419	0.417	-1.006	0.080	TG 18:0_32:1	0.796176473
CountryMexico	0.185	0.184	1.004	0.093	TG 16:0_36:3	0.796433474
CountryMexico	0.124	0.125	1.000	0.069	C3	0.799544403
CountryMexico	-0.342	0.342	-1.000	0.065	TG 18:1_33:0	0.799544403
CountryMexico	0.082	0.082	0.998	0.048	LPI 18:1	0.800131331
CountryMexico	0.113	0.114	0.995	0.097	PG 18:1_20:5	0.800342785
CountryMexico	0.080	0.080	0.996	0.066	Mannose	0.800342785
CountryMexico	0.202	0.203	0.993	0.091	LPG 14:0	0.800847391

CountryMexico	-0.534	0.540	-0.988	0.018	TG 18:1_26:0	0.805160419
CountryMexico	-0.182	0.185	-0.987	0.017	PE 36:2	0.805861192
CountryMexico	-0.290	0.294	-0.986	0.052	Xanthurenic acid	0.806686742
CountryMexico	-0.248	0.253	-0.979	0.018	TG 18:0_36:5	0.811336065
CountryMexico	0.178	0.183	0.973	0.091	TG 18:2_34:1	0.815633025
CountryMexico	-0.207	0.214	-0.968	0.091	TG 16:0_37:3	0.81685067
CountryMexico	-0.211	0.219	-0.965	0.096	Cer d18:0/24:1	0.819143037
CountryMexico	-0.308	0.321	-0.960	0.060	DiCA 12:0	0.821138352
CountryMexico	-0.160	0.168	-0.957	0.058	Cer d18:2/24:0	0.82201371
CountryMexico	-0.109	0.115	-0.953	0.055	PG 22:4_22:6	0.822351061
CountryMexico	-0.183	0.193	-0.950	0.019	TG 18:2_38:5	0.823101139
CountryMexico	-0.202	0.213	-0.949	0.029	GAMT Deficiency	0.824005995
CountryMexico	-0.432	0.458	-0.944	0.075	4-EPS	0.826362516
CountryMexico	-0.173	0.185	-0.939	0.051	CE 22:6	0.826794632
CountryMexico	0.109	0.116	0.942	0.021	Orn Synthesis	0.826794632
CountryMexico	-0.059	0.063	-0.941	0.061	Ratio of PUFA-PIs to SFA-PIs	0.826794632
CountryMexico	-0.057	0.061	-0.938	0.055	Ratio of UFA-PIs to SFA-PIs	0.82759926
CountryMexico	-0.104	0.111	-0.937	0.033	Sum of UFA-LPGs	0.828250934
CountryMexico	0.853	0.919	0.928	0.068	SPHK activity (4)	0.832348024
CountryMexico	-0.853	0.919	-0.928	0.068	LPP3 activity (4)	0.832348024
CountryMexico	0.201	0.218	0.926	0.040	4-Guanidinobutanoic acid	0.833477325
CountryMexico	-0.260	0.281	-0.925	0.076	TG 22:5_32:1	0.833893495
CountryMexico	0.201	0.218	0.920	0.098	t4-OH-Pro	0.835282342
CountryMexico	-0.104	0.113	-0.921	0.019	Cer d18:1/24:0	0.835282342
CountryMexico	-0.137	0.149	-0.921	0.210	Vanillylmandelic acid	0.835282342
CountryMexico	-0.281	0.308	-0.913	0.035	TG 16:0_32:3	0.836014266
CountryMexico	-0.126	0.138	-0.911	0.050	Hex-Cer d18:2/23:0	0.836178985
CountryMexico	-0.201	0.220	-0.910	0.062	LPS 20:1	0.836178985
CountryMexico	0.182	0.200	0.910	0.062	Biliverdin	0.836178985
CountryMexico	0.068	0.075	0.908	0.040	SPBP d14:1	0.83670792
CountryMexico	-0.515	0.569	-0.905	0.022	TG 18:0_30:1	0.839492953
CountryMexico	0.195	0.216	0.904	0.033	TG 18:1_36:5	0.839568031
CountryMexico	-0.136	0.153	-0.891	0.032	PI 18:1_20:5	0.842057457
CountryMexico	0.081	0.091	0.895	0.035	PG 18:2_20:5	0.842057457
CountryMexico	0.254	0.285	0.892	0.050	TMAO Synthesis (direct)	0.842057457
CountryMexico	0.148	0.166	0.890	0.033	IBD Deficiency (NBS)	0.842057457
CountryMexico	0.182	0.205	0.886	0.104	TG 18:1_34:3	0.84391978
CountryMexico	-0.319	0.361	-0.884	0.029	ProBetaine	0.8449897
CountryMexico	0.326	0.370	0.880	0.084	DG 14:1_18:1	0.846270213
CountryMexico	-0.104	0.118	-0.882	0.076	FA 24:4n-6	0.846270213
CountryMexico	-0.360	0.412	-0.874	0.031	TG 18:0_32:2	0.847307863

CountryMexico	-0.098	0.112	-0.873	0.031	Sum of AAA-derived Lactic Acids	0.847802961
CountryMexico	0.105	0.122	0.865	0.083	PA 18:1_22:1	0.851141565
CountryMexico	0.140	0.162	0.861	0.041	PA 18:1_18:4	0.852316061
CountryMexico	-0.221	0.257	-0.860	0.062	TG 16:1_38:5	0.853538645
CountryMexico	0.158	0.185	0.853	0.047	FA 4:0-2M	0.854441147
CountryMexico	-0.056	0.066	-0.845	0.042	FA 4:0	0.856858178
CountryMexico	-0.181	0.215	-0.842	0.034	TG 18:0_36:3	0.856998891
CountryMexico	0.080	0.095	0.837	0.023	3-Met-2-oxovaleric acid	0.858411328
CountryMexico	0.148	0.177	0.837	0.052	LPI 22:0	0.858534902
CountryMexico	0.099	0.119	0.836	0.029	HSer to Creatinine Ratio	0.858981624
CountryMexico	-0.474	0.569	-0.834	0.030	DG 16:1_18:0	0.859320896
CountryMexico	-0.177	0.213	-0.834	0.091	LPS 18:2	0.859320896
CountryMexico	0.373	0.448	0.834	0.038	Sum of Unconjugated Primary BAs	0.859320896
CountryMexico	-0.288	0.347	-0.830	0.139	IAG	0.861344099
CountryMexico	-0.166	0.200	-0.829	0.068	TG 16:0_40:7	0.861604978
CountryMexico	-0.083	0.100	-0.827	0.050	PA 18:1_20:0	0.861979889
CountryMexico	-0.347	0.420	-0.826	0.015	Primary BA Conjugation	0.862018337
CountryMexico	0.060	0.073	0.826	0.024	PC 36:2	0.862141302
CountryMexico	-0.097	0.117	-0.825	0.077	Cer d18:1/23:0	0.862975487
CountryMexico	-0.296	0.360	-0.822	0.023	TG 17:1_36:5	0.864654653
CountryMexico	-0.242	0.297	-0.816	0.154	DHEAS	0.867480579
CountryMexico	-0.190	0.233	-0.816	0.075	PE 32:1	0.867480579
CountryMexico	0.125	0.153	0.816	0.252	N-Ac-Putrescine	0.867480579
CountryMexico	-0.335	0.411	-0.815	0.048	TG 16:1_30:1	0.867896696
CountryMexico	0.109	0.135	0.813	0.036	Symmetrical Arg Methylation	0.869277197
CountryMexico	0.062	0.077	0.809	0.014	PC 32:3	0.869565295
CountryMexico	-0.169	0.209	-0.810	0.045	TG 18:2_33:2	0.869565295
CountryMexico	-0.051	0.063	-0.809	0.031	Ratio of MUFA-PIs to SFA-PIs	0.869565295
CountryMexico	0.188	0.234	0.806	0.104	DG 16:1_18:2	0.870246055
CountryMexico	0.067	0.083	0.801	0.122	PA 14:0_14:1	0.871772124
CountryMexico	0.093	0.116	0.801	0.042	PA 16:0_18:2	0.871772124
CountryMexico	0.157	0.195	0.804	0.061	PA 18:1_20:3	0.871772124
CountryMexico	0.079	0.098	0.800	0.012	PG 18:0_22:1	0.871772124
CountryMexico	-0.383	0.482	-0.793	0.080	TG 16:0_38:1	0.872661621
CountryMexico	-0.201	0.252	-0.797	0.041	TG 18:0_34:3	0.872661621
CountryMexico	0.175	0.221	0.794	0.028	TG 18:3_36:3	0.872661621
CountryMexico	0.087	0.110	0.792	0.042	BVRA Activity	0.872661621
CountryMexico	-0.111	0.141	-0.788	0.023	PE P-18:1/18:1	0.873211377
CountryMexico	0.259	0.330	0.787	0.070	TG 17:2_36:4	0.873211377
CountryMexico	0.189	0.240	0.786	0.032	Creatine to Creatinine Ratio	0.873516102
CountryMexico	0.104	0.133	0.784	0.017	PC 32:2	0.874202189

CountryMexico	-0.249	0.318	-0.783	0.023	TG 14:0_38:4	0.874395202
CountryMexico	0.139	0.179	0.780	0.078	PE P-18:0/18:2	0.875580329
CountryMexico	-0.066	0.084	-0.779	0.099	PG 18:1_22:4	0.875580329
CountryMexico	0.093	0.119	0.777	0.017	Uridine	0.875702921
CountryMexico	-0.151	0.195	-0.777	0.018	PI 18:1_22:4	0.875702921
CountryMexico	0.242	0.311	0.778	0.068	3-Met-His to 1-Met-His Ratio	0.875702921
CountryMexico	-0.123	0.158	-0.774	0.163	Cer d18:2/23:0	0.877461625
CountryMexico	-0.166	0.215	-0.772	0.041	5-Amino-4-oxovaleric acid	0.877871344
CountryMexico	0.073	0.094	0.773	0.026	PI 17:1_18:2	0.877871344
CountryMexico	-0.443	0.578	-0.767	0.047	Cer d18:1/26:1	0.878863772
CountryMexico	-0.124	0.161	-0.768	0.098	PE 38:6	0.878863772
CountryMexico	-0.365	0.475	-0.768	0.011	TG 18:2_28:0	0.878863772
CountryMexico	-0.330	0.428	-0.770	0.014	Gly Conjugation of Primary BAs	0.878863772
CountryMexico	-0.324	0.424	-0.764	0.037	TG 18:3_30:0	0.880010242
CountryMexico	-0.090	0.118	-0.762	0.037	N-Ac-Glu	0.88159885
CountryMexico	0.105	0.137	0.762	0.076	PA 18:1_22:2	0.88159885
CountryMexico	-0.089	0.118	-0.759	0.021	LPG 16:0	0.88159885
CountryMexico	-0.105	0.138	-0.760	0.050	LPG 18:1	0.88159885
CountryMexico	0.074	0.098	0.759	0.075	CPT-2 Deficiency (NBS)	0.88159885
CountryMexico	-0.150	0.199	-0.753	0.054	TG 16:0_38:5	0.885950818
CountryMexico	0.164	0.218	0.750	0.088	TG 20:2_34:1	0.886103407
CountryMexico	0.112	0.150	0.748	0.030	LPI 19:0	0.886235944
CountryMexico	-0.158	0.212	-0.746	0.138	CE 18:0	0.886402257
CountryMexico	-0.088	0.117	-0.747	0.045	CACT Deficiency (NBS)	0.886402257
CountryMexico	0.088	0.117	0.747	0.045	CPT-1 Deficiency (NBS)	0.886402257
CountryMexico	-0.337	0.454	-0.743	0.026	Taurine Conjugation of Primary BAs	0.887834689
CountryMexico	0.150	0.202	0.742	0.015	BAIBA	0.888469436
CountryMexico	-0.119	0.161	-0.741	0.056	N-Ac-Pro	0.888469436
CountryMexico	0.197	0.266	0.741	0.035	PA 18:2_22:4	0.888469436
CountryMexico	-0.063	0.089	-0.710	0.050	FA 2:0	0.89033088
CountryMexico	-0.293	0.407	-0.719	0.045	2-OH-Benzoic acid	0.89033088
CountryMexico	0.111	0.155	0.716	0.080	PA 18:2_20:0	0.89033088
CountryMexico	0.126	0.177	0.711	0.058	LPG 16:1	0.89033088
CountryMexico	-0.074	0.104	-0.711	0.090	PC O-34:3	0.89033088
CountryMexico	0.053	0.075	0.716	0.050	PG 18:2_22:1	0.89033088
CountryMexico	-0.302	0.421	-0.716	0.050	TG 14:0_32:2	0.89033088
CountryMexico	0.304	0.414	0.734	0.057	TG 20:1_32:0	0.89033088
CountryMexico	-0.076	0.106	-0.715	0.039	Sum of Carboxylic Acids	0.89033088
CountryMexico	-0.071	0.100	-0.707	0.041	Sum of VLCFA-Cer	0.89033088
CountryMexico	0.056	0.078	0.722	0.057	Sum of LCFA-LPIs	0.89033088
CountryMexico	0.315	0.442	0.713	0.047	7a-Dehydroxylation of CA	0.89033088

CountryMexico	0.080	0.113	0.708	0.033	Urea to Creatinine Ratio	0.89033088
CountryMexico	-0.055	0.075	-0.730	0.060	FA 16:0 to FA 18:1 Ratio	0.89033088
CountryMexico	0.100	0.139	0.720	0.011	Cortisone Synthesis	0.89033088
CountryMexico	-0.112	0.155	-0.722	0.066	MC Deficiency (NBS)	0.89033088
CountryMexico	0.112	0.155	0.722	0.066	PA (NBS)	0.89033088
CountryMexico	0.156	0.222	0.703	0.055	C14:2-OH	0.891499549
CountryMexico	-0.147	0.212	-0.695	0.033	C5-OH (C3-DC-M)	0.897008532
CountryMexico	0.128	0.185	0.694	0.066	PA 16:2_18:1	0.89707141
CountryMexico	-0.051	0.074	-0.693	0.045	LPE 22:0	0.897545831
CountryMexico	0.090	0.130	0.692	0.163	LPI 14:0	0.897545831
CountryMexico	0.283	0.408	0.693	0.012	p-Cresol-SO4 to p-Cresol Glucuronide	0.897545831
CountryMexico	-0.073	0.106	-0.691	0.024	PG 22:5_22:6	0.89771212
CountryMexico	0.135	0.197	0.686	0.024	C16:1-OH	0.898103103
CountryMexico	0.183	0.267	0.684	0.026	DiCA 14:0	0.898103103
CountryMexico	0.074	0.108	0.684	0.155	PI 18:2_22:0	0.898103103
CountryMexico	-0.158	0.232	-0.679	0.011	C5-DC (C6-OH)	0.898286684
CountryMexico	-0.113	0.166	-0.677	0.069	LPA 14:1	0.898286684
CountryMexico	-0.234	0.344	-0.679	0.021	TG 18:2_30:1	0.898286684
CountryMexico	0.127	0.188	0.674	0.051	TG 18:1_36:4	0.898694799
CountryMexico	0.184	0.273	0.673	0.131	TG 16:1_34:1	0.898824989
CountryMexico	0.112	0.166	0.673	0.053	SCAD Deficiency (NBS)	0.898824989
CountryMexico	-0.156	0.234	-0.665	0.019	Cholesterol Synthesis	0.902980367
CountryMexico	-0.164	0.249	-0.659	0.162	Sum of Steroid Hormones	0.904084918
CountryMexico	-0.069	0.105	-0.662	0.072	FA 2:0 to Glucose Ratio	0.904084918
CountryMexico	-0.170	0.259	-0.656	0.012	C9	0.904940218
CountryMexico	-0.160	0.244	-0.656	0.061	DG 18:2_20:4	0.904940218
CountryMexico	0.049	0.075	0.655	0.062	PG 16:0_22:1	0.905123395
CountryMexico	-0.057	0.087	-0.648	0.076	FA 24:1n-9	0.905904979
CountryMexico	-0.261	0.406	-0.642	0.033	IsoLCA	0.907380555
CountryMexico	0.140	0.217	0.644	0.082	TG 18:1_34:4	0.907380555
CountryMexico	-0.249	0.390	-0.639	0.042	LPE P-14:0	0.908928691
CountryMexico	0.146	0.228	0.639	0.077	Pro Hydroxylation	0.90900853
CountryMexico	0.316	0.495	0.638	0.016	TG 18:3_34:0	0.909091752
CountryMexico	-0.077	0.121	-0.636	0.038	Sum of MUFA-LPGs	0.909778426
CountryMexico	-0.181	0.286	-0.634	0.118	TG 20:3_32:1	0.909786066
CountryMexico	-0.062	0.099	-0.632	0.060	PG 18:1_18:1	0.910503371
CountryMexico	0.083	0.131	0.632	0.046	FA16:0 to FA 16:1 Ratio	0.910503371
CountryMexico	-0.084	0.134	-0.628	0.013	HCys	0.911021528
CountryMexico	-0.109	0.173	-0.628	0.045	IVA (NBS)	0.911021528
CountryMexico	-0.084	0.135	-0.626	0.019	SBCAD Deficiency (NBS)	0.911388227
CountryMexico	-0.145	0.232	-0.625	0.085	TG 17:0_34:1	0.911954172

CountryMexico	-0.043	0.068	-0.624	0.175	Glx to N-Ac-Asp Ratio	0.911954172
CountryMexico	-0.066	0.106	-0.621	0.052	Hex-Cer d18:1/20:0	0.912485066
CountryMexico	0.093	0.150	0.620	0.022	PE P-18:0/16:0	0.912485066
CountryMexico	0.043	0.070	0.620	0.084	Ratio of PUFA-PCs O to MUFA-PCs O	0.912485066
CountryMexico	0.098	0.161	0.607	0.054	AABA	0.913408785
CountryMexico	0.042	0.069	0.606	0.023	Val	0.913408785
CountryMexico	0.064	0.104	0.616	0.069	Cer d18:1/24:1	0.913408785
CountryMexico	0.120	0.199	0.604	0.039	Hex-Cer d18:2/20:0	0.913408785
CountryMexico	-0.080	0.131	-0.610	0.020	LPI 20:4	0.913408785
CountryMexico	0.096	0.157	0.613	0.023	3-OH-Isobutyric acid	0.913408785
CountryMexico	0.090	0.146	0.614	0.036	PA 18:2_18:2	0.913408785
CountryMexico	-0.058	0.095	-0.608	0.084	PI 16:0_18:3	0.913408785
CountryMexico	-0.073	0.118	-0.616	0.047	PI 18:2_20:0	0.913408785
CountryMexico	-0.114	0.189	-0.604	0.042	TG 18:1_38:6	0.913408785
CountryMexico	-0.152	0.247	-0.615	0.031	TG 18:2_32:2	0.913408785
CountryMexico	-0.107	0.175	-0.611	0.084	TG 20:3_36:3	0.913408785
CountryMexico	0.046	0.076	0.603	0.055	Sum of LPIs	0.913408785
CountryMexico	-0.118	0.194	-0.606	0.011	ACY1 Deficiency	0.913408785
CountryMexico	-0.075	0.123	-0.608	0.060	LDH Activity	0.913408785
CountryMexico	-0.315	0.527	-0.597	0.042	CMPF	0.91401703
CountryMexico	-0.083	0.138	-0.598	0.023	Phenyl-Lac	0.91401703
CountryMexico	-0.064	0.107	-0.598	0.102	PC 38:3	0.91401703
CountryMexico	-0.250	0.416	-0.600	0.076	TG 20:3_36:5	0.91401703
CountryMexico	-0.263	0.439	-0.599	0.060	Sum of 12a-OH BAs	0.91401703
CountryMexico	-0.057	0.097	-0.593	0.061	PI 18:2_22:6	0.914423252
CountryMexico	-0.042	0.071	-0.592	0.011	Sum of PUFA-PIs	0.914423252
CountryMexico	-0.041	0.068	-0.593	0.014	Sum of UFA-PIs	0.914423252
CountryMexico	0.251	0.425	0.590	0.019	12-KetoDCA to DCA Ratio	0.914423252
CountryMexico	0.063	0.109	0.581	0.035	C0	0.914922917
CountryMexico	0.088	0.152	0.579	0.048	C2	0.914922917
CountryMexico	-0.035	0.060	-0.581	0.042	ADMA	0.914922917
CountryMexico	-0.069	0.119	-0.579	0.032	Lac	0.914922917
CountryMexico	-0.064	0.110	-0.584	0.101	CerP d18:1/16:0	0.914922917
CountryMexico	0.219	0.376	0.583	0.072	3-OH-Sebacic acid	0.914922917
CountryMexico	0.099	0.172	0.577	0.076	LPI 18:2	0.914922917
CountryMexico	0.054	0.094	0.573	0.030	PI 18:0_18:1	0.914922917
CountryMexico	-0.055	0.094	-0.584	0.032	PI 18:2_20:5	0.914922917
CountryMexico	-0.050	0.087	-0.573	0.087	PG 18:2_20:0	0.914922917
CountryMexico	-0.115	0.199	-0.577	0.066	TG 17:0_34:2	0.914922917
CountryMexico	0.113	0.196	0.578	0.100	TG 17:1_36:3	0.914922917
CountryMexico	-0.178	0.311	-0.572	0.020	TG 18:2_30:0	0.914922917

CountryMexico	-0.105	0.184	-0.571	0.076	TG 18:2_35:2	0.914922917
CountryMexico	0.092	0.159	0.578	0.054	Sum of Aminobutyric Acids	0.914922917
CountryMexico	0.168	0.288	0.581	0.078	Xanthine Synthesis	0.914922917
CountryMexico	0.061	0.105	0.583	0.038	MMA (NBS)	0.914922917
CountryMexico	0.055	0.097	0.567	0.064	Pro	0.916510777
CountryMexico	-0.038	0.066	-0.565	0.049	4-OH-Phenylpyruvic acid	0.916729216
CountryMexico	-0.234	0.414	-0.566	0.007	TG 16:0_30:2	0.916729216
CountryMexico	-0.297	0.532	-0.559	0.057	DCA	0.917790309
CountryMexico	0.094	0.168	0.558	0.041	PA 18:1_22:3	0.917790309
CountryMexico	0.083	0.150	0.558	0.029	PG 18:0_18:2	0.917790309
CountryMexico	-0.122	0.218	-0.558	0.091	TG 16:0_35:3	0.917790309
CountryMexico	0.143	0.257	0.557	0.067	TG 18:3_35:2	0.917790309
CountryMexico	0.111	0.200	0.554	0.013	FA 18:3	0.918913652
CountryMexico	0.053	0.097	0.549	0.063	PA 18:2_20:1	0.91933565
CountryMexico	0.033	0.061	0.552	0.030	Sum of SFA-LPIs	0.91933565
CountryMexico	-0.085	0.156	-0.542	0.051	Cer d18:2/22:0	0.9204228
CountryMexico	0.054	0.100	0.545	0.017	PG 18:2_22:4	0.9204228
CountryMexico	0.076	0.141	0.543	0.027	PG 22:6_22:6	0.9204228
CountryMexico	0.133	0.244	0.545	0.054	TG 18:1_32:0	0.9204228
CountryMexico	0.043	0.080	0.536	0.043	Muscle Protein Degradation	0.921613943
CountryMexico	-0.071	0.133	-0.532	0.022	N1-Ac-Spermidine	0.922372951
CountryMexico	0.082	0.154	0.531	0.063	Sum of PUFA-LPIs	0.922782695
CountryMexico	0.031	0.059	0.530	0.054	Valinemia (NBS)	0.922999129
CountryMexico	0.157	0.297	0.529	0.101	TG 14:0_36:2	0.92332277
CountryMexico	0.044	0.084	0.523	0.049	PI 18:0_18:0	0.92450393
CountryMexico	-0.149	0.285	-0.521	0.062	TG 16:0_33:1	0.92450393
CountryMexico	-0.151	0.290	-0.521	0.045	TG 18:2_33:0	0.92450393
CountryMexico	-0.104	0.200	-0.520	0.088	TG 18:2_33:1	0.92450393
CountryMexico	-0.156	0.302	-0.518	0.019	TG 16:0_40:8	0.924595921
CountryMexico	0.108	0.210	0.518	0.007	BAIBA Synthesis	0.924595921
CountryMexico	0.164	0.319	0.513	0.058	DG 18:1_18:3	0.925185866
CountryMexico	-0.184	0.359	-0.512	0.074	TG 14:0_38:5	0.925185866
CountryMexico	0.130	0.254	0.513	0.065	TG 16:0_34:1	0.925185866
CountryMexico	-0.106	0.209	-0.508	0.043	DG 18:1_20:4	0.926862898
CountryMexico	-0.034	0.067	-0.509	0.024	Sum of MUFA-PIs	0.926862898
CountryMexico	0.170	0.335	0.507	0.063	TG 17:2_34:2	0.928019016
CountryMexico	0.250	0.499	0.501	0.043	3-IPA	0.929951194
CountryMexico	-0.112	0.224	-0.499	0.033	PE P-18:0/14:0	0.929951194
CountryMexico	-0.117	0.233	-0.502	0.021	PE P-18:0/20:2	0.929951194
CountryMexico	-0.275	0.548	-0.501	0.004	TG 16:0_28:1	0.929951194
CountryMexico	-0.062	0.125	-0.499	0.084	Gly to Ala Ratio	0.929951194

CountryMexico	0.064	0.128	0.498	0.051	PA 18:1_20:2	0.930429319
CountryMexico	-0.033	0.066	-0.497	0.016	Sum of PIs	0.930575858
CountryMexico	-0.205	0.415	-0.494	0.072	GLCAS	0.931229276
CountryMexico	-0.161	0.326	-0.494	0.036	TG 16:0_32:2	0.931229276
CountryMexico	0.121	0.247	0.492	0.038	TG 17:1_36:4	0.931229276
CountryMexico	-0.128	0.260	-0.494	0.029	TG 20:1_32:3	0.931229276
CountryMexico	0.104	0.212	0.492	0.091	TG 20:2_34:2	0.931229276
CountryMexico	-0.219	0.442	-0.495	0.019	Gly Conjugation of DCA	0.931229276
CountryMexico	-0.231	0.472	-0.489	0.052	Taurine Conjugation of DCA	0.932254227
CountryMexico	0.105	0.215	0.488	0.053	DG 18:2_18:2	0.933412361
CountryMexico	-0.200	0.411	-0.486	0.080	LPG 14:1	0.933935607
CountryMexico	-0.152	0.315	-0.483	0.044	CE 22:2	0.934202878
CountryMexico	-0.157	0.324	-0.483	0.057	TG 14:0_34:3	0.934202878
CountryMexico	-0.180	0.378	-0.476	0.004	TG 18:0_38:7	0.934202878
CountryMexico	-0.100	0.207	-0.484	0.042	Hex3Cer d18:1/24:1	0.934202878
CountryMexico	-0.031	0.065	-0.477	0.016	Sum of (L)PIs	0.934202878
CountryMexico	0.045	0.094	0.483	0.049	Sum of UFA-LPIs	0.934202878
CountryMexico	-0.034	0.071	-0.480	0.111	Ratio of PUFA-LPCs to SFA-LPCs	0.934202878
CountryMexico	-0.127	0.270	-0.471	0.031	TMAO Synthesis	0.936181229
CountryMexico	0.043	0.091	0.471	0.099	Sum of MUFA-PAs	0.93628788
CountryMexico	0.133	0.283	0.470	0.038	Cer d18:1/26:0	0.936700062
CountryMexico	-0.035	0.075	-0.468	0.020	PI 16:1_18:2	0.936700062
CountryMexico	0.126	0.270	0.467	0.113	TG 18:1_32:1	0.936700062
CountryMexico	0.152	0.325	0.467	0.064	TG 20:2_32:0	0.936700062
CountryMexico	0.053	0.113	0.464	0.057	PA 18:1_18:2	0.937503096
CountryMexico	0.053	0.115	0.464	0.102	PI 18:1_20:0	0.937543055
CountryMexico	-0.281	0.607	-0.464	0.053	SPBP d14:0	0.937543055
CountryMexico	-0.118	0.257	-0.460	0.015	TG 14:0_36:4	0.939125854
CountryMexico	-0.223	0.488	-0.456	0.030	TG 16:0_28:2	0.940600234
CountryMexico	-0.077	0.170	-0.453	0.071	PE 33:2	0.941316941
CountryMexico	0.189	0.420	0.450	0.059	TrpBetaine	0.942567747
CountryMexico	-0.227	0.507	-0.447	0.042	TG 18:0_30:0	0.943367741
CountryMexico	-0.083	0.185	-0.446	0.034	SM 38:3	0.943409458
CountryMexico	-0.118	0.266	-0.444	0.119	MG 20:1	0.944129441
CountryMexico	0.095	0.218	0.437	0.045	5-AVA	0.944324442
CountryMexico	-0.102	0.233	-0.437	0.055	PE 28:0	0.944324442
CountryMexico	0.059	0.133	0.441	0.055	PI 18:1_20:3	0.944324442
CountryMexico	0.070	0.160	0.438	0.026	PG 16:0_16:0	0.944324442
CountryMexico	-0.032	0.074	-0.440	0.029	PG 18:1_18:2	0.944324442
CountryMexico	-0.132	0.301	-0.440	0.007	TG 14:0_40:5	0.944324442
CountryMexico	0.093	0.209	0.443	0.080	TG 18:1_35:3	0.944324442

CountryMexico	0.078	0.176	0.440	0.098	Ratio of HArg to SDMA	0.944324442
CountryMexico	0.062	0.142	0.440	0.067	Ratio of PUFA-LPIs to MUFA-LPIs	0.944324442
CountryMexico	0.056	0.130	0.433	0.042	PG 16:2_18:2	0.94592227
CountryMexico	0.108	0.248	0.434	0.036	TG 22:5_32:0	0.94592227
CountryMexico	-0.110	0.257	-0.430	0.053	TG 16:1_36:5	0.946050549
CountryMexico	-0.111	0.259	-0.429	0.033	TG 18:0_36:2	0.946050549
CountryMexico	0.088	0.205	0.429	0.039	TG 18:3_34:1	0.946050549
CountryMexico	0.086	0.202	0.425	0.037	DG 16:0_18:2	0.946902937
CountryMexico	-0.064	0.152	-0.422	0.065	HArg	0.947227209
CountryMexico	-0.072	0.171	-0.423	0.005	FA 20:3n-6	0.947227209
CountryMexico	-0.032	0.076	-0.421	0.049	PI 15:1_16:0	0.947555697
CountryMexico	-0.046	0.111	-0.418	0.007	Cer d18:2/16:0	0.948379179
CountryMexico	0.045	0.108	0.416	0.031	DLD (NBS)	0.948604678
CountryMexico	-0.275	0.663	-0.415	0.049	SPHK activity (6)	0.948660588
CountryMexico	-0.106	0.257	-0.414	0.063	TG 18:1_31:0	0.948738381
CountryMexico	0.106	0.256	0.413	0.128	Phenylglucuronide	0.949979614
CountryMexico	0.033	0.079	0.411	0.064	PI 15:0_16:0	0.950306327
CountryMexico	-0.152	0.370	-0.410	0.008	TG 20:1_32:2	0.950306327
CountryMexico	-0.077	0.190	-0.405	0.012	N-Ac-Trp	0.950395586
CountryMexico	-0.030	0.073	-0.409	0.077	PG 16:0_18:2	0.950395586
CountryMexico	0.077	0.196	0.396	0.090	TG 22:5_34:1	0.951141183
CountryMexico	-0.134	0.338	-0.396	0.056	TG 20:3_34:0	0.951151408
CountryMexico	0.076	0.194	0.393	0.043	TG 18:2_36:3	0.951771697
CountryMexico	0.045	0.114	0.391	0.027	PG 16:1_18:2	0.952790918
CountryMexico	0.035	0.090	0.391	0.026	Sum of Sulfur-Containing AAs	0.952790918
CountryMexico	-0.037	0.096	-0.389	0.051	PS 36:5	0.952862176
CountryMexico	-0.094	0.244	-0.386	0.095	TG 16:0_35:2	0.952862176
CountryMexico	0.125	0.324	0.386	0.038	Taurine Conjugation of UDCA	0.952862176
CountryMexico	-0.082	0.213	-0.384	0.046	FA 16:1	0.953194155
CountryMexico	-0.137	0.358	-0.382	0.012	TG 18:1_30:2	0.953506789
CountryMexico	0.084	0.223	0.378	0.149	TG 18:1_33:1	0.954167936
CountryMexico	-0.065	0.173	-0.373	0.018	FA 20:2	0.954169483
CountryMexico	-0.042	0.112	-0.376	0.060	PG 18:1_22:1	0.954169483
CountryMexico	0.039	0.105	0.368	0.013	PG 18:2_22:3	0.954169483
CountryMexico	-0.079	0.212	-0.373	0.111	TG 17:1_34:2	0.954169483
CountryMexico	-0.095	0.254	-0.372	0.021	TG 18:3_32:0	0.954169483
CountryMexico	0.115	0.309	0.372	0.140	TG 20:1_32:1	0.954169483
CountryMexico	0.063	0.175	0.360	0.022	PE 34:3	0.956073178
CountryMexico	-0.065	0.179	-0.361	0.091	TG 17:0_36:3	0.956073178
CountryMexico	0.093	0.262	0.355	0.095	TG 16:1_34:3	0.956770865
CountryMexico	0.149	0.424	0.352	0.020	TG 17:2_36:2	0.956860885

CountryMexico	-0.075	0.214	-0.352	0.133	TG 20:3_34:1	0.956860885
CountryMexico	0.086	0.245	0.353	0.006	CMAMMA	0.956860885
CountryMexico	0.027	0.080	0.338	0.006	a-Ketoisovaleric acid	0.959319434
CountryMexico	0.054	0.162	0.333	0.018	LPI 17:0	0.959319434
CountryMexico	-0.064	0.191	-0.337	0.006	PE 36:3	0.959319434
CountryMexico	0.029	0.084	0.341	0.063	PI 14:0_18:2	0.959319434
CountryMexico	0.058	0.170	0.339	0.066	PC 32:1	0.959319434
CountryMexico	-0.109	0.326	-0.336	0.085	TG 16:1_32:2	0.959319434
CountryMexico	0.076	0.225	0.339	0.065	TG 16:1_36:4	0.959319434
CountryMexico	-0.093	0.277	-0.334	0.064	TG 18:3_32:1	0.959319434
CountryMexico	0.082	0.245	0.332	0.073	TG 22:6_34:1	0.959319434
CountryMexico	-0.086	0.254	-0.338	0.045	TG 22:6_34:2	0.959319434
CountryMexico	0.023	0.068	0.335	0.018	Sum of BCAAs	0.959319434
CountryMexico	-0.065	0.196	-0.330	0.004	Cer d18:2/20:0	0.959403841
CountryMexico	0.056	0.172	0.324	0.049	FA 18:1	0.960574279
CountryMexico	0.067	0.208	0.321	0.117	PE P-16:0/18:3	0.960877313
CountryMexico	0.049	0.152	0.319	0.038	Ratio of PUFA-LPIs to SFA-LPIs	0.960877313
CountryMexico	0.050	0.167	0.303	0.088	PE P-18:0/20:3	0.965204506
CountryMexico	0.064	0.215	0.296	0.052	MG 22:2	0.967943463
CountryMexico	-0.077	0.258	-0.297	0.030	TG 22:5_34:3	0.967943463
CountryMexico	-0.080	0.271	-0.297	0.072	IsoUDCA to UDCA Ratio	0.967943463
CountryMexico	0.018	0.062	0.289	0.077	Ser	0.968500369
CountryMexico	0.057	0.194	0.293	0.126	TG 18:1_33:2	0.968500369
CountryMexico	0.019	0.068	0.287	0.053	Sum of MUFA-LPIs	0.968500369
CountryMexico	0.069	0.240	0.288	0.075	Kynurenic Acid to Xanthurenic Acid Ratio	0.968500369
CountryMexico	0.028	0.101	0.274	0.027	Cys	0.969297514
CountryMexico	0.046	0.165	0.280	0.117	LPA 15:0	0.969297514
CountryMexico	0.027	0.097	0.276	0.094	PA 18:1_20:1	0.969297514
CountryMexico	-0.047	0.172	-0.274	0.010	PA 18:2_22:3	0.969297514
CountryMexico	0.038	0.138	0.277	0.011	PI 18:2_20:1	0.969297514
CountryMexico	0.044	0.161	0.276	0.021	Isonicotinic acid	0.969297514
CountryMexico	0.102	0.362	0.282	0.106	TG 16:1_38:3	0.969297514
CountryMexico	0.081	0.286	0.284	0.002	TG 17:2_36:3	0.969297514
CountryMexico	0.066	0.239	0.275	0.014	TG 18:3_34:3	0.969297514
CountryMexico	-0.015	0.055	-0.276	0.020	Sum of Essential AAs	0.969297514
CountryMexico	0.101	0.369	0.274	0.077	Ketone Body Synthesis	0.969297514
CountryMexico	0.057	0.208	0.273	0.027	NAT10 Activity	0.969297514
CountryMexico	-0.028	0.102	-0.273	0.073	PI 18:1_22:5	0.969350525
CountryMexico	-0.032	0.117	-0.271	0.031	Sum of FA 12:0 - FA 16:0	0.969533946
CountryMexico	0.022	0.081	0.270	0.022	SPBP d17:1	0.969780456
CountryMexico	0.063	0.234	0.268	0.034	PA 16:0_18:3	0.969791157

CountryMexico	-0.084	0.315	-0.266	0.047	TG 22:6_32:0	0.969791157
CountryMexico	-0.096	0.362	-0.265	0.044	DG 16:0_16:1	0.970486873
CountryMexico	0.048	0.180	0.265	0.029	FA 18:2	0.970486873
CountryMexico	0.024	0.092	0.265	0.058	PG 18:2_18:2	0.970486873
CountryMexico	0.032	0.123	0.263	0.014	LPI 16:1	0.970542986
CountryMexico	0.038	0.145	0.263	0.040	N-Ac-Cytidine	0.970542986
CountryMexico	-0.026	0.100	-0.262	0.014	PI 18:1_18:3	0.970542986
CountryMexico	0.025	0.095	0.262	0.042	PG 18:2_18:3	0.970542986
CountryMexico	0.060	0.226	0.264	0.031	TG 18:2_34:3	0.970542986
CountryMexico	0.058	0.223	0.261	0.052	TG 18:2_35:3	0.970640252
CountryMexico	0.030	0.115	0.261	0.065	PA 18:2_22:0	0.970822832
CountryMexico	0.020	0.077	0.256	0.020	PG 18:0_18:3	0.971019688
CountryMexico	0.041	0.160	0.255	0.027	PG 17:0_18:2	0.971049176
CountryMexico	-0.014	0.055	-0.254	0.058	Ratio of MUFA-LPIs to SFA-LPIs	0.971120038
CountryMexico	-0.058	0.229	-0.254	0.005	TG 16:0_36:5	0.97117192
CountryMexico	-0.066	0.264	-0.250	0.026	C5:1	0.972286142
CountryMexico	0.127	0.540	0.236	0.056	GCA	0.972286142
CountryMexico	-0.058	0.231	-0.250	0.010	LPI 17:1	0.972286142
CountryMexico	0.045	0.181	0.249	0.026	PE 34:2	0.972286142
CountryMexico	-0.089	0.367	-0.244	0.009	PS 32:0	0.972286142
CountryMexico	-0.023	0.097	-0.237	0.053	PC O-34:2	0.972286142
CountryMexico	-0.045	0.195	-0.231	0.038	PG 14:0_16:0	0.972286142
CountryMexico	-0.028	0.114	-0.241	0.095	PG 18:1_22:0	0.972286142
CountryMexico	0.057	0.232	0.245	0.064	TG 16:0_34:2	0.972286142
CountryMexico	-0.062	0.251	-0.246	0.067	TG 17:0_34:3	0.972286142
CountryMexico	-0.060	0.255	-0.237	0.105	TG 17:1_34:1	0.972286142
CountryMexico	-0.022	0.095	-0.231	0.057	Sum BCFA	0.972286142
CountryMexico	0.040	0.173	0.232	0.048	Sum of MUFAs	0.972286142
CountryMexico	0.017	0.073	0.235	0.056	Sum of SFA-PIs	0.972286142
CountryMexico	-0.035	0.141	-0.250	0.030	Sum of Spha	0.972286142
CountryMexico	0.041	0.181	0.228	0.032	LPG 20:1	0.97264658
CountryMexico	0.059	0.260	0.226	0.042	TG 16:0_34:4	0.97264658
CountryMexico	-0.008	0.036	-0.228	0.021	Ratio of PUFA-PIs to MUFA-PIs	0.97264658
CountryMexico	-0.078	0.350	-0.221	0.099	DG 18:1_20:2	0.973245899
CountryMexico	0.007	0.030	0.222	0.036	Indoleacetaldehyde	0.973245899
CountryMexico	0.041	0.185	0.221	0.026	PS 34:2	0.973245899
CountryMexico	-0.086	0.395	-0.219	0.008	Gly Conjugation of UDCA	0.974082151
CountryMexico	-0.033	0.151	-0.218	0.060	AMSDH Saturation	0.974199342
CountryMexico	0.082	0.379	0.215	0.021	C3-DC (C4-OH)	0.974241494
CountryMexico	-0.044	0.202	-0.216	0.020	Creatine	0.974241494
CountryMexico	-0.027	0.126	-0.217	0.098	PI 18:0_20:0	0.974241494

CountryMexico	0.021	0.097	0.215	0.084	PG 18:1_20:4	0.974241494
CountryMexico	-0.015	0.068	-0.216	0.032	BCAT Val Catabolism	0.974241494
CountryMexico	-0.036	0.167	-0.214	0.046	CE 22:5	0.974380169
CountryMexico	-0.024	0.115	-0.210	0.029	PI 18:1_22:0	0.975050491
CountryMexico	-0.020	0.096	-0.209	0.004	Picolinic acid	0.975050491
CountryMexico	0.054	0.256	0.211	0.047	TG 14:0_36:3	0.975050491
CountryMexico	0.037	0.174	0.210	0.016	MA (NBS)	0.975050491
CountryMexico	0.016	0.075	0.206	0.083	PC 34:1	0.975414812
CountryMexico	0.024	0.115	0.205	0.028	PI 16:0_16:0	0.975417945
CountryMexico	0.096	0.469	0.205	0.052	GDCA Synthesis from CA	0.975417945
CountryMexico	-0.043	0.218	-0.198	0.023	TG 18:2_36:4	0.977246508
CountryMexico	-0.078	0.394	-0.198	0.056	TG 22:6_34:3	0.977246508
CountryMexico	-0.055	0.280	-0.195	0.033	GLCA	0.977553454
CountryMexico	-0.082	0.421	-0.196	0.016	TG 18:1_28:1	0.977553454
CountryMexico	0.049	0.249	0.196	0.051	TG 18:1_33:3	0.977553454
CountryMexico	0.023	0.121	0.192	0.013	Glutaminolysis Rate	0.978070109
CountryMexico	-0.076	0.417	-0.181	0.015	LPG 17:1	0.978081461
CountryMexico	-0.027	0.141	-0.192	0.035	PE P-18:1/22:6	0.978081461
CountryMexico	0.022	0.121	0.179	0.073	PI 18:1_22:2	0.978081461
CountryMexico	-0.025	0.133	-0.186	0.016	PC 38:6	0.978081461
CountryMexico	-0.025	0.136	-0.185	0.074	PC 40:6	0.978081461
CountryMexico	-0.025	0.135	-0.185	0.070	PG 18:0_18:1	0.978081461
CountryMexico	-0.055	0.299	-0.184	0.112	TG 16:1_33:1	0.978081461
CountryMexico	-0.067	0.350	-0.191	0.043	TG 18:1_30:0	0.978081461
CountryMexico	-0.046	0.247	-0.185	0.034	TG 18:1_32:3	0.978081461
CountryMexico	-0.030	0.162	-0.183	0.091	Ratio of HArg to ADMA	0.978081461
CountryMexico	0.084	0.457	0.184	0.094	TDCA Synthesis from CA	0.978081461
CountryMexico	0.025	0.137	0.180	0.041	Ratio of Acetylcarnitine to Carnitine	0.978081461
CountryMexico	0.019	0.106	0.177	0.121	Kynurenine	0.978946317
CountryMexico	0.046	0.258	0.177	0.072	TG 18:1_32:2	0.978946317
CountryMexico	0.043	0.244	0.175	0.010	TG 18:2_36:5	0.979042918
CountryMexico	0.045	0.261	0.173	0.014	TG 18:3_36:1	0.979042918
CountryMexico	0.023	0.131	0.176	0.042	b-Oxidation	0.979042918
CountryMexico	0.011	0.062	0.176	0.054	Ratio of UFA-LPCs to SFA-LPCs	0.979042918
CountryMexico	0.010	0.059	0.172	0.060	Phe	0.979544567
CountryMexico	-0.017	0.104	-0.168	0.065	Choline to Creatinine Ratio	0.981505603
CountryMexico	0.034	0.203	0.167	0.034	TG 18:2_34:2	0.981630677
CountryMexico	0.042	0.256	0.165	0.095	TG 16:1_34:2	0.982740199
CountryMexico	-0.034	0.207	-0.164	0.033	TG 18:2_38:4	0.982881418
CountryMexico	0.032	0.199	0.163	0.038	2-OH-Butyric acid	0.982939275
CountryMexico	-0.035	0.220	-0.161	0.017	TG 18:3_34:2	0.983666899

CountryMexico	0.083	0.521	0.160	0.029	Indole Pathway Activity	0.983666899
CountryMexico	-0.021	0.143	-0.147	0.017	C5	0.984282992
CountryMexico	0.052	0.357	0.147	0.065	3-Met-His	0.984282992
CountryMexico	0.021	0.137	0.151	0.090	PA 18:1_22:0	0.984282992
CountryMexico	0.027	0.177	0.151	0.047	PG 16:0_18:1	0.984282992
CountryMexico	0.040	0.255	0.157	0.040	TG 20:2_32:1	0.984282992
CountryMexico	-0.074	0.495	-0.149	0.013	Sum of Gly-Conjugated BAs	0.984282992
CountryMexico	0.013	0.087	0.144	0.051	PI 16:0_22:1	0.985310026
CountryMexico	-0.046	0.334	-0.139	0.081	12-KetoDCA	0.985627802
CountryMexico	0.026	0.187	0.137	0.043	PI 18:1_22:3	0.985627802
CountryMexico	-0.044	0.322	-0.137	0.035	TG 14:0_34:2	0.985627802
CountryMexico	-0.041	0.303	-0.137	0.016	TG 16:0_32:0	0.985627802
CountryMexico	-0.054	0.381	-0.141	0.038	TG 18:1_30:1	0.985627802
CountryMexico	0.025	0.186	0.137	0.148	TG 18:1_35:2	0.985627802
CountryMexico	0.034	0.244	0.140	0.029	TG 18:2_34:4	0.985627802
CountryMexico	0.066	0.483	0.136	0.017	Sum of Primary BAs	0.985627802
CountryMexico	0.012	0.089	0.136	0.016	Ratio of UFA-LPIs to SFA-LPIs	0.985627802
CountryMexico	0.045	0.356	0.127	0.083	3-OH-Butyric acid	0.986052128
CountryMexico	-0.064	0.513	-0.125	0.022	DG 18:2_20:0	0.986052128
CountryMexico	0.013	0.097	0.132	0.099	PI 16:0_17:0	0.986052128
CountryMexico	0.020	0.152	0.129	0.022	PI 18:1_20:1	0.986052128
CountryMexico	0.006	0.047	0.128	0.075	Glucose	0.986052128
CountryMexico	-0.033	0.260	-0.127	0.001	TG 16:0_36:6	0.986052128
CountryMexico	-0.032	0.240	-0.132	0.069	TG 16:0_38:4	0.986052128
CountryMexico	-0.028	0.215	-0.131	0.020	TG 18:2_32:0	0.986052128
CountryMexico	-0.008	0.059	-0.129	0.078	MCKAT Deficiency (NBS)	0.986052128
CountryMexico	0.042	0.343	0.124	0.051	TG 16:0_32:1	0.986376292
CountryMexico	0.012	0.105	0.118	0.023	C16	0.986778539
CountryMexico	-0.066	0.537	-0.122	0.024	TCA	0.986778539
CountryMexico	0.023	0.190	0.121	0.014	LPA 14:0	0.986778539
CountryMexico	0.026	0.219	0.118	0.056	MG 22:1	0.986778539
CountryMexico	-0.013	0.110	-0.116	0.093	PI 18:2_18:3	0.986778539
CountryMexico	-0.038	0.317	-0.121	0.042	TG 22:6_32:1	0.986778539
CountryMexico	0.014	0.128	0.112	0.095	HArg Synthesis	0.988376477
CountryMexico	0.012	0.110	0.109	0.038	PI 18:0_22:0	0.988734432
CountryMexico	0.019	0.198	0.097	0.031	FA 22:6n-3 (DHA)	0.991000108
CountryMexico	0.019	0.191	0.101	0.019	PG 17:0_18:1	0.991000108
CountryMexico	-0.009	0.095	-0.099	0.100	PG 18:1_20:3	0.991000108
CountryMexico	0.013	0.132	0.097	0.070	Quinolinic acid	0.991000108
CountryMexico	-0.025	0.253	-0.099	0.021	TG 20:0_32:4	0.991000108
CountryMexico	0.007	0.074	0.091	0.023	Ile	0.991121731

CountryMexico	-0.020	0.245	-0.082	0.067	Itaconic acid	0.991121731
CountryMexico	-0.017	0.201	-0.083	0.065	FA 8:0	0.991121731
CountryMexico	-0.019	0.235	-0.080	0.040	Cytidine	0.991121731
CountryMexico	0.017	0.178	0.094	0.050	PE P-16:0/16:0	0.991121731
CountryMexico	0.022	0.245	0.088	0.047	PE P-18:0/20:1	0.991121731
CountryMexico	-0.011	0.113	-0.095	0.054	PI 14:0_18:1	0.991121731
CountryMexico	0.015	0.193	0.076	0.060	TG 18:2_35:1	0.991121731
CountryMexico	-0.026	0.274	-0.095	0.013	TG 18:3_36:4	0.991121731
CountryMexico	-0.018	0.199	-0.089	0.042	TG 22:5_34:2	0.991121731
CountryMexico	0.014	0.189	0.075	0.054	Cer d18:1/25:0	0.991386868
CountryMexico	0.008	0.109	0.074	0.017	OH-Phenyl-Lac	0.991392622
CountryMexico	-0.013	0.199	-0.067	0.086	C14	0.991588357
CountryMexico	0.004	0.068	0.058	0.041	Lys	0.991588357
CountryMexico	0.029	0.506	0.058	0.002	TCDCA	0.991588357
CountryMexico	0.008	0.157	0.049	0.041	Cer d18:2/24:1	0.991588357
CountryMexico	0.004	0.078	0.056	0.068	Fumaric acid	0.991588357
CountryMexico	0.013	0.258	0.050	0.069	LPE P-22:6	0.991588357
CountryMexico	-0.005	0.082	-0.063	0.070	PI 16:0_17:2	0.991588357
CountryMexico	0.008	0.140	0.055	0.016	PI 16:0_20:0	0.991588357
CountryMexico	-0.006	0.096	-0.062	0.089	PG 18:1_22:2	0.991588357
CountryMexico	-0.020	0.359	-0.055	0.047	TG 14:0_34:1	0.991588357
CountryMexico	-0.017	0.329	-0.053	0.079	TG 16:0_35:1	0.991588357
CountryMexico	-0.019	0.330	-0.057	0.055	TG 16:1_32:0	0.991588357
CountryMexico	0.020	0.287	0.068	0.138	TG 17:1_34:3	0.991588357
CountryMexico	0.011	0.234	0.049	0.067	TG 18:2_32:1	0.991588357
CountryMexico	0.026	0.504	0.052	0.012	Sum of Conjugated Primary BAs	0.991588357
CountryMexico	0.008	0.133	0.063	0.031	Sum of SFA-PGs	0.991588357
CountryMexico	-0.006	0.100	-0.056	0.109	AGAT Deficiency	0.991588357
CountryMexico	-0.007	0.108	-0.069	0.016	Acetic Acid to Butyric Acid Ratio	0.991588357
CountryMexico	0.008	0.138	0.055	0.087	5-HIAA to Creatinine Ratio	0.991588357
CountryMexico	0.008	0.170	0.047	0.067	PE 35:3	0.991779428
CountryMexico	-0.005	0.110	-0.047	0.002	Cys Synthesis	0.991779428
CountryMexico	-0.005	0.103	-0.047	0.016	GABR	0.991785958
CountryMexico	-0.008	0.181	-0.044	0.038	FA 20:1n-9	0.991846161
CountryMexico	0.016	0.355	0.044	0.095	TG 16:1_32:1	0.991846161
CountryMexico	-0.017	0.432	-0.040	0.003	TG 18:1_36:0	0.991915638
CountryMexico	-0.005	0.147	-0.037	0.000	Lac to FA 2:0 Ratio	0.991915638
CountryMexico	-0.007	0.199	-0.033	0.003	SPB d14:0	0.992832957
CountryMexico	0.005	0.162	0.030	0.051	PE P-20:0/18:2	0.992951143
CountryMexico	0.002	0.073	0.025	0.019	Leu	0.993726388
CountryMexico	0.013	0.520	0.026	0.009	GCDCA	0.993726388

CountryMexico	0.003	0.095	0.028	0.061	PC O-36:3	0.993726388
CountryMexico	0.005	0.233	0.021	0.058	TG 16:0_34:3	0.995394412
CountryMexico	0.005	0.271	0.017	0.031	TMAO	0.996431003
CountryMexico	0.001	0.095	0.014	0.133	3-MAG-uria Type 1	0.997553117
CountryMexico	0.002	0.165	0.012	0.102	TG 18:1_38:5	0.997872437
CountryMexico	-0.001	0.061	-0.011	0.030	Sum of Solely Ketogenic AAs	0.998516427
CountryMexico	0.001	0.119	0.008	0.033	FA 16:0	0.99908292
CountryMexico	0.001	0.207	0.004	0.023	PA 16:1_22:0	0.999254492
CountryMexico	0.000	0.200	0.002	0.026	TG 16:0_36:4	0.999254492
CountryMexico	0.001	0.312	0.004	0.002	TG 18:1_36:6	0.999254492
CountryMexico	-0.001	0.151	-0.006	0.056	BHMT Activity	0.999254492

SUPPLEMENTAL DATA 2

Short hand name	Metabolite name	Analyte class	HMDB ID
C0	Carnitine	Acylcarnitines	HMDB00062
C2	Acetylcarnitine	Acylcarnitines	HMDB00201
C3	Propionylcarnitine	Acylcarnitines	HMDB00824
C3-DC (C4-OH)	Malonylcarnitine (Hydroxybutyrylcarnitine)	Acylcarnitines	HMDB02095
C4	Butyrylcarnitine	Acylcarnitines	HMDB02013
C4:1	Butenylcarnitine	Acylcarnitines	HMDB0013126
C5	Valerylcarnitine	Acylcarnitines	HMDB13128
C5-DC (C6-OH)	Glutarylcarnitine (Hydroxyhexanoylcarnitine)	Acylcarnitines	HMDB0013130
C5-OH (C3-DC-M)	Hydroxyvalerylcarnitine (Methylmalonylcarnitine)	Acylcarnitines	HMDB0013132
C5:1	Tiglylcarnitine	Acylcarnitines	HMDB0002366
C8	Octanoylcarnitine	Acylcarnitines	HMDB0000791
C9	Nonanoylcarnitine	Acylcarnitines	HMDB06320
C10	Decanoylcarnitine	Acylcarnitines	HMDB00651
C10:1	Decenoylcarnitine	Acylcarnitines	HMDB13205
C10:2	Decadienoylcarnitine	Acylcarnitines	HMDB0013325
C12	Dodecanoylcarnitine	Acylcarnitines	HMDB02250
C12:1	Dodecenoylcarnitine	Acylcarnitines	HMDB0013326
C14	Tetradecanoylcarnitine	Acylcarnitines	HMDB05066
C14:1	Tetradecenoylcarnitine	Acylcarnitines	HMDB0002014
C14:1-OH	Hydroxytetradecenoylcarnitine	Acylcarnitines	HMDB0013330
C14:2	Tetradecadienoylcarnitine	Acylcarnitines	HMDB13331
C14:2-OH	Hydroxytetradecadienoylcarnitine	Acylcarnitines	NA
C16	Hexadecanoylcarnitine	Acylcarnitines	HMDB00222
C16:1	Hexadecenoylcarnitine	Acylcarnitines	HMDB06317
C16:1-OH	Hydroxyhexadecenoylcarnitine	Acylcarnitines	HMDB13333
C16:2	Hexadecadienoylcarnitine	Acylcarnitines	HMDB13334
C18	Octadecanoylcarnitine	Acylcarnitines	HMDB00848
C18:1	Octadecenoylcarnitine	Acylcarnitines	HMDB0006351
C18:1-OH	Hydroxyoctadecenoylcarnitine	Acylcarnitines	HMDB13339
C18:2	Octadecadienoylcarnitine	Acylcarnitines	HMDB0006469
Trigonelline	Trigonelline	Alkaloids	HMDB0000875
TMAO	Trimethylamine N-oxide	Amine oxides	HMDB0000925
1-Met-His	1-Methylhistidine	Amino acid-related	HMDB0000001
3-Met-2-oxovaleric acid	3-Methyl-2-oxovaleric acid	Amino acid-related	HMDB0000491
3-Met-His	3-Methylhistidine	Amino acid-related	HMDB0000479
4-Guanidinobutanoic acid	4-Guanidinobutanoic acid	Amino acid-related	HMDB0003464
4-Met-2-oxovaleric acid	2-Oxoisocaproic acid	Amino acid-related	HMDB0000695
5-Amino-4-oxovaleric acid	5-Amino-4-oxovaleric acid	Amino acid-related	HMDB0001149
5-AVA	5-Aminovaleric acid	Amino acid-related	HMDB0003355

5-Oxo-Pro	5-Oxoproline	Amino acid-related	HMDB0000267
a-Ketoisovaleric acid	alpha-Ketoisovaleric acid	Amino acid-related	HMDB0000019
AABA	alpha-Aminobutyric acid	Amino acid-related	HMDB0000452
ADMA	Asymmetric dimethylarginine	Amino acid-related	HMDB0001539
alpha-AAA	alpha-Aminoadipic acid	Amino acid-related	HMDB0000510
Anthranilic acid	Anthranilic acid	Amino acid-related	HMDB0001123
Arginino-Suc	Argininosuccinic acid	Amino acid-related	HMDB0000052
BABA	beta-Aminobutyric acid	Amino acid-related	HMDB0031654
BAIBA	3-Aminoisobutyric acid	Amino acid-related	HMDB0003911
Betaine	Betaine	Amino acid-related	HMDB0000043
c4-OH-Pro	cis-4-Hydroxyproline	Amino acid-related	HMDB0240251
Carnosine	Carnosine	Amino acid-related	HMDB0000033
Cinnamoyl-Gly	Cinnamoylglycine	Amino acid-related	HMDB0011621
Cit	Citrulline	Amino acid-related	HMDB0000904
Creatine	Creatine	Amino acid-related	HMDB0000064
Creatinine	Creatinine	Amino acid-related	HMDB0000562
Cystine	Cystine	Amino acid-related	HMDB0000192
DMG	Dimethylglycine (DMG)	Amino acid-related	HMDB0000092
Guanidinoacetic acid	Guanidinoacetic acid	Amino acid-related	HMDB0000128
HArg	Homoarginine	Amino acid-related	HMDB0000670
HCit	Homocitrulline	Amino acid-related	HMDB0000679
HCys	Homocysteine	Amino acid-related	HMDB0000742
HSer	Homoserine	Amino acid-related	HMDB0000719
Imidazolepropionic acid	Imidazolepropionic acid	Amino acid-related	HMDB0002271
Kynurenine	Kynurenine	Amino acid-related	HMDB0000684
Met-SO	Methionine sulfoxide	Amino acid-related	HMDB0002005
N-Ac-Ala	N-Acetylalanine	Amino acid-related	HMDB0255053
N-Ac-Arg	N-Acetylarginine	Amino acid-related	HMDB0004620
N-Ac-Asn	N-Acetylasparagine	Amino acid-related	HMDB0006028
N-Ac-Asp	N-Acetylaspartic acid	Amino acid-related	HMDB0000812
N-Ac-Gln	N-Acetylglutamine	Amino acid-related	HMDB0006029
N-Ac-Glu	N-Acetylglutamic acid	Amino acid-related	HMDB0001138
N-Ac-Gly	N-Acetylglycine	Amino acid-related	HMDB0000532
N-Ac-His	N-Acetylhistidine	Amino acid-related	HMDB0032055
N-Ac-Ile	N-Acetylisoleucine	Amino acid-related	HMDB0061684
N-Ac-Leu	N-Acetylleucine	Amino acid-related	HMDB0341345
N-Ac-Met	N-Acetylmethionine	Amino acid-related	HMDB0011745
N-Ac-Pro	N-Acetylproline	Amino acid-related	HMDB0094701
N-Ac-Ser	N-Acetylserine	Amino acid-related	HMDB0002931
N-Ac-Trp	N-Acetyltryptophan	Amino acid-related	HMDB0255052
N-Ac-Tyr	N-Acetyltyrosine	Amino acid-related	HMDB0244966

N-Ac-Val	N-Acetylvaline	Amino acid-related	HMDB0011757
N-Met-Asp	N-Methylaspartic acid	Amino acid-related	HMDB0002393
N2-Ac-Lys	N2-Acetyllysine	Amino acid-related	HMDB0000446
N6-Ac-Lys	N6-Acetyllysine	Amino acid-related	HMDB0000206
Orn	Ornithine	Amino acid-related	HMDB0000214
Phenylacetylglutamine	Phenylacetylglutamine	Amino acid-related	HMDB0006344
ProBetaine	Proline betaine	Amino acid-related	HMDB0004827
Sarcosine	Sarcosine	Amino acid-related	HMDB0000271
SDMA	Symmetric dimethylarginine	Amino acid-related	HMDB0003334
t4-OH-Pro	trans-4-Hydroxyproline	Amino acid-related	HMDB0000725
Taurine	Taurine	Amino acid-related	HMDB0000251
TrpBetaine	Tryptophan betaine	Amino acid-related	HMDB0061115
Ala	Alanine	Amino acids	HMDB0000161
Arg	Arginine	Amino acids	HMDB0000517
Asn	Asparagine	Amino acids	HMDB0000168
Asp	Aspartic acid	Amino acids	HMDB0000191
Cys	Cysteine	Amino acids	HMDB0000574
Gln	Glutamine	Amino acids	HMDB0000641
Glu	Glutamic acid	Amino acids	HMDB0000148
Gly	Glycine	Amino acids	HMDB0000123
His	Histidine	Amino acids	HMDB0000177
Ile	Isoleucine	Amino acids	HMDB0000172
Leu	Leucine	Amino acids	HMDB0000687
Lys	Lysine	Amino acids	HMDB0000182
Met	Methionine	Amino acids	HMDB0000696
Phe	Phenylalanine	Amino acids	HMDB0000159
Pro	Proline	Amino acids	HMDB0000162
Ser	Serine	Amino acids	HMDB0000187
Thr	Threonine	Amino acids	HMDB0000167
Trp	Tryptophan	Amino acids	HMDB0000929
Tyr	Tyrosine	Amino acids	HMDB0000158
Val	Valine	Amino acids	HMDB0000883
3-EpiDCA	3-Epideoxycholic acid	Bile acids	HMDB0000438
7-KetoDCA	7-Ketodeoxycholic acid	Bile acids	HMDB0000391
12-KetoDCA	12-Ketodeoxycholic acid	Bile acids	HMDB0000328
CA	Cholic acid	Bile acids	HMDB0000619
CDCA	Chenodeoxycholic acid	Bile acids	HMDB0000518
DCA	Deoxycholic acid	Bile acids	HMDB0000626
GCA	Glycocholic acid	Bile acids	HMDB0000138
GCDCA	Glycochenodeoxycholic acid	Bile acids	HMDB0000637
GDCA	Glycodeoxycholic acid	Bile acids	HMDB0000631

GLCA	Glycolithocholic acid	Bile acids	HMDB0000698
GLCAS	Glycolithocholic acid sulfate	Bile acids	HMDB0002639
GUDCA	Glycoursodeoxycholic acid	Bile acids	HMDB0000708
IsoLCA	Isolithocholic acid	Bile acids	HMDB0000717
IsoUDCA	Isoursodeoxycholic acid	Bile acids	HMDB0000686
NorDCA	Nordeoxycholic acid	Bile acids	HMDB0304947
TCA	Taurocholic acid	Bile acids	HMDB0000036
TCDCA	Taurochenodeoxycholic acid	Bile acids	HMDB0000951
TDCA	Taurodeoxycholic acid	Bile acids	HMDB0000896
THDCA	Taurohyodeoxycholic acid	Bile acids	NA
TLCA	Taurolithocholic acid	Bile acids	HMDB0000722
TUDCA	Tauroursodeoxycholic acid	Bile acids	HMDB0000874
UDCA	Ursodeoxycholic acid	Bile acids	HMDB0000946
beta-Ala	beta-Alanine	Biogenic amines	HMDB0000056
GABA	gamma-Aminobutyric acid	Biogenic amines	HMDB0000112
Histamine	Histamine	Biogenic amines	HMDB0000870
Putrescine	Putrescine	Biogenic amines	HMDB0001414
Serotonin	Serotonin	Biogenic amines	HMDB0000259
Spermidine	Spermidine	Biogenic amines	HMDB0001257
Urea	Urea	Biogenic amines	HMDB0000294
2-OH-Butyric acid	2-Hydroxybutyric acid	Carboxylic acids	HMDB0000008
3-OH-Butyric acid	3-Hydroxybutyric acid	Carboxylic acids	HMDB0000442
Glycolic acid	Glycolic acid	Carboxylic acids	HMDB0000115
Glyoxylic acid	Glyoxylic acid	Carboxylic acids	HMDB0000119
HipAcid	Hippuric acid	Carboxylic acids	HMDB0000714
Lac	Lactic acid	Carboxylic acids	HMDB0000190
Mevalonic acid	Mevalonic acid	Carboxylic acids	HMDB0000227
Pyruvic acid	Pyruvic acid	Carboxylic acids	HMDB0000243
Cer d16:1/18:0	Ceramide d16:1/18:0	Ceramides	NA
Cer d16:1/20:0	Ceramide d16:1/20:0	Ceramides	NA
Cer d16:1/22:0	Ceramide d16:1/22:0	Ceramides	NA
Cer d16:1/23:0	Ceramide d16:1/23:0	Ceramides	NA
Cer d16:1/24:0	Ceramide d16:1/24:0	Ceramides	NA
Cer d18:1/16:0	Ceramide d18:1/16:0	Ceramides	HMDB0004949
Cer d18:1/18:0	Ceramide d18:1/18:0	Ceramides	HMDB0004950
Cer d18:1/20:0	Ceramide d18:1/20:0	Ceramides	HMDB0004951
Cer d18:1/22:0	Ceramide d18:1/22:0	Ceramides	HMDB0004952
Cer d18:1/23:0	Ceramide d18:1/23:0	Ceramides	HMDB0000950
Cer d18:1/24:0	Ceramide d18:1/24:0	Ceramides	HMDB0004956
Cer d18:1/24:1	Ceramide d18:1/24:1	Ceramides	HMDB0004953
Cer d18:1/25:0	Ceramide d18:1/25:0	Ceramides	HMDB0004957

Cer d18:1/26:0	Ceramide d18:1/26:0	Ceramides	HMDB0004955
Cer d18:1/26:1	Ceramide d18:1/26:1	Ceramides	HMDB0004954
Cer d18:2/16:0	Ceramide d18:2/16:0	Ceramides	NA
Cer d18:2/18:0	Ceramide d18:2/18:0	Ceramides	NA
Cer d18:2/20:0	Ceramide d18:2/20:0	Ceramides	NA
Cer d18:2/22:0	Ceramide d18:2/22:0	Ceramides	NA
Cer d18:2/23:0	Ceramide d18:2/23:0	Ceramides	NA
Cer d18:2/24:0	Ceramide d18:2/24:0	Ceramides	NA
Cer d18:2/24:1	Ceramide d18:2/24:1	Ceramides	NA
CerP d18:1/16:0	Ceramide phosphate d18:1/16:0	Ceramides	HMDB0010700
CE 14:0	Cholesteryl ester 14:0	Cholesteryl esters	HMDB0006725
CE 16:0	Cholesteryl ester 16:0	Cholesteryl esters	HMDB00885
CE 16:1	Cholesteryl ester 16:1	Cholesteryl esters	HMDB00658
CE 17:1	Cholesteryl ester 17:1	Cholesteryl esters	HMDB0060060
CE 18:0	Cholesteryl ester 18:0	Cholesteryl esters	HMDB0062461
CE 18:1	Cholesteryl ester 18:1	Cholesteryl esters	HMDB00918
CE 18:2	Cholesteryl ester 18:2	Cholesteryl esters	HMDB05192
CE 18:3	Cholesteryl ester 18:3	Cholesteryl esters	HMDB0010370
CE 20:3	Cholesteryl ester 20:3	Cholesteryl esters	HMDB06736
CE 20:4	Cholesteryl ester 20:4	Cholesteryl esters	HMDB06726
CE 20:5	Cholesteryl ester 20:5	Cholesteryl esters	HMDB06731
CE 22:2	Cholesteryl ester 22:2	Cholesteryl esters	HMDB06737
CE 22:5	Cholesteryl ester 22:5	Cholesteryl esters	HMDB10374
CE 22:6	Cholesteryl ester 22:6	Cholesteryl esters	HMDB06733
p-Cresol glucuronide	p-Cresol glucuronide	Cresols	HMDB0011686
p-Cresol-SO4	p-Cresol sulfate	Cresols	HMDB0011635
2-OH-Glutaric acid	2-Hydroxyglutaric acid	Dicarboxylic acids	HMDB0000694
3-HMGA	3-Hydroxymethylglutaric acid	Dicarboxylic acids	HMDB0000355
3-Met-adipic acid	3-Methyladipic acid	Dicarboxylic acids	HMDB0000555
3-Met-glutaric acid	3-Methylglutaric acid	Dicarboxylic acids	HMDB0000752
3-OH-Glutaric acid	3-Hydroxyglutaric acid	Dicarboxylic acids	HMDB0000428
3-OH-Sebacic acid	3-Hydroxysebacic acid	Dicarboxylic acids	HMDB0000350
a-Ketoglutaric acid	alpha-Ketoglutaric acid	Dicarboxylic acids	HMDB0000208
Adipic acid	Adipic acid	Dicarboxylic acids	HMDB0000448
Citramalic acid	Citramalic acid	Dicarboxylic acids	HMDB0000426
DiCA 12:0	Dodecanedioic acid	Dicarboxylic acids	HMDB0000623
DiCA 14:0	Tetradecanedioic acid	Dicarboxylic acids	HMDB0000872
E-3-Met-glutaconic acid	E-3-methylglutaconic acid	Dicarboxylic acids	HMDB0000522
Ethylmalonic acid	Ethylmalonic acid	Dicarboxylic acids	HMDB0000622
Fumaric acid	Fumaric acid	Dicarboxylic acids	HMDB0000134
Glutaric acid	Glutaric acid	Dicarboxylic acids	HMDB0000661

Itaconic acid	Itaconic acid	Dicarboxylic acids	HMDB0002092
Maleic acid	Maleic acid	Dicarboxylic acids	HMDB0000176
Malic acid	Malic acid	Dicarboxylic acids	HMDB0000156
Malonic acid	Malonic acid	Dicarboxylic acids	HMDB0000691
Methylmalonic acid	Methylmalonic acid	Dicarboxylic acids	HMDB0000202
Oxalic acid	Oxalic acid	Dicarboxylic acids	HMDB0002329
Oxaloacetic acid	Oxaloacetic acid	Dicarboxylic acids	HMDB0000223
Suberic acid	Suberic acid	Dicarboxylic acids	HMDB0000893
Suc	Succinic acid	Dicarboxylic acids	HMDB0000254
Z-3-Met-glutaconic acid	Z-3-Methylglutaconic acid	Dicarboxylic acids	NA
DG 14:1_18:1	Diacylglyceride 14:1_18:1	Diglycerides	HMDB0007044
DG 16:0_16:1	Diacylglyceride 16:0_16:1	Diglycerides	HMDB0007099
DG 16:0_18:1	Diacylglyceride 16:0_18:1	Diglycerides	HMDB0007214
DG 16:0_18:2	Diacylglyceride 16:0_18:2	Diglycerides	HMDB0007103
DG 16:1_18:0	Diacylglyceride 16:1_18:0	Diglycerides	HMDB0007129
DG 16:1_18:1	Diacylglyceride 16:1_18:1	Diglycerides	HMDB0007131
DG 16:1_18:2	Diacylglyceride 16:1_18:2	Diglycerides	HMDB0007132
DG 17:0_18:1	Diacylglyceride 17:0_18:1	Diglycerides	NA
DG 18:1_18:1	Diacylglyceride 18:1_18:1	Diglycerides	HMDB0007218
DG 18:1_18:2	Diacylglyceride 18:1_18:2	Diglycerides	HMDB0007219
DG 18:1_18:3	Diacylglyceride 18:1_18:3	Diglycerides	HMDB0007221
DG 18:1_20:1	Diacylglyceride 18:1_20:1	Diglycerides	HMDB0007224
DG 18:1_20:2	Diacylglyceride 18:1_20:2	Diglycerides	HMDB0007225
DG 18:1_20:4	Diacylglyceride 18:1_20:4	Diglycerides	HMDB0007228
DG 18:2_18:2	Diacylglyceride 18:2_18:2	Diglycerides	HMDB0007248
DG 18:2_18:4	Diacylglyceride 18:2_18:4	Diglycerides	HMDB0007335
DG 18:2_20:0	Diacylglyceride 18:2_20:0	Diglycerides	HMDB0007252
DG 18:2_20:4	Diacylglyceride 18:2_20:4	Diglycerides	HMDB0007257
Hex2Cer d18:1/14:0	Dihexosylceramide d18:1/14:0	Dihexosylceramides	NA
Hex2Cer d18:1/16:0	Dihexosylceramide d18:1/16:0	Dihexosylceramides	HMDB0004833
Hex2Cer d18:1/18:0	Dihexosylceramide d18:1/18:0	Dihexosylceramides	HMDB0004834
Hex2Cer d18:1/22:0	Dihexosylceramide d18:1/22:0	Dihexosylceramides	HMDB0004836
Hex2Cer d18:1/24:0	Dihexosylceramide d18:1/24:0	Dihexosylceramides	HMDB0004840
Hex2Cer d18:1/24:1	Dihexosylceramide d18:1/24:1	Dihexosylceramides	HMDB0004837
Cer d18:0/22:0	Ceramide d18:0/22:0	Dihydroceramides	HMDB0011765
Cer d18:0/24:1	Ceramide d18:0/24:1	Dihydroceramides	HMDB0011769
FA 2:0	Acetic acid	Fatty acids	HMDB0000042
FA 3:0-2M	Isobutyric acid	Fatty acids	HMDB0001873
FA 4:0	Butyric acid	Fatty acids	HMDB0000039
FA 4:0-2M	2-Methylbutyric acid	Fatty acids	HMDB0002176
FA 4:0-3M	Isovaleric acid	Fatty acids	HMDB0000718

FA 5:0-3M	3-Methylvaleric acid	Fatty acids	HMDB0033774
FA 5:0-4M	Isocaproic acid	Fatty acids	HMDB0000689
FA 6:0	Caproic acid	Fatty acids	HMDB0000535
FA 7:0	Heptanoic acid	Fatty acids	HMDB0000666
FA 8:0	Caprylic acid	Fatty acids	HMDB0000482
FA 9:0	Nonanoic acid	Fatty acids	HMDB0000847
FA 10:0	Capric acid	Fatty acids	HMDB0000511
FA 11:0	Undecanoic acid	Fatty acids	HMDB0000947
FA 12:0	Lauric acid	Fatty acids	HMDB0000638
FA 14:0	Myristic acid	Fatty acids	HMDB0000806
FA 14:1n-5	Myristoleic acid	Fatty acids	HMDB0002000
FA 15:0	Pentadecanoic acid	Fatty acids	HMDB0000826
FA 16:0	Palmitic acid	Fatty acids	HMDB0000220
FA 16:1	Palmitoleic acid	Fatty acids	HMDB0012328
FA 18:0	Stearic acid	Fatty acids	HMDB0000827
FA 18:1	Oleic acid	Fatty acids	HMDB0000573
FA 18:2	Linoleic acid	Fatty acids	HMDB0000673
FA 18:3	Octadecatrienoic acid	Fatty acids	HMDB0001388
FA 20:0	Arachidic acid	Fatty acids	HMDB0002212
FA 20:1n-9	Gondoic acid	Fatty acids	HMDB0002231
FA 20:2	Eicosadienoic acid	Fatty acids	HMDB0005060
FA 20:3n-6	Dihomo-gamma-linolenic acid (omega6)	Fatty acids	HMDB0002925
FA 20:3n-9	Mead acid	Fatty acids	HMDB0010378
FA 20:4n-6 (AA)	Arachidonic acid (AA, omega6)	Fatty acids	HMDB0001043
FA 20:5n-3 (EPA)	Eicosapentaenoic acid (EPA, omega3)	Fatty acids	HMDB0001999
FA 22:4n-6	Docosatetraenoic acid (omega6)	Fatty acids	HMDB0002226
FA 22:5n-3 (DPA)	Docosapentaenoic acid (DPA, omega3)	Fatty acids	HMDB0001976
FA 22:6n-3 (DHA)	Docosahexaenoic acid (DHA, omega3)	Fatty acids	HMDB0002183
FA 24:0	Lignoceric acid	Fatty acids	HMDB0002003
FA 24:1n-9	Nervonic acid	Fatty acids	HMDB0002368
FA 24:4n-6	Tetracosatetraenoic acid (omega6)	Fatty acids	HMDB0006246
Hex-Cer d16:1/20:0	Hexosylceramide d16:1/20:0	Hexosylceramides	NA
Hex-Cer d18:1/14:0	Hexosylceramide d18:1/14:0	Hexosylceramides	HMDB0012321
Hex-Cer d18:1/16:0	Hexosylceramide d18:1/16:0	Hexosylceramides	HMDB0004971
Hex-Cer d18:1/18:0	Hexosylceramide d18:1/18:0	Hexosylceramides	HMDB0010709
Hex-Cer d18:1/18:1	Hexosylceramide d18:1/18:1	Hexosylceramides	HMDB0010714
Hex-Cer d18:1/20:0	Hexosylceramide d18:1/20:0	Hexosylceramides	HMDB0010710
Hex-Cer d18:1/22:0	Hexosylceramide d18:1/22:0	Hexosylceramides	HMDB0010711
Hex-Cer d18:1/23:0	Hexosylceramide d18:1/23:0	Hexosylceramides	NA
Hex-Cer d18:1/24:0	Hexosylceramide d18:1/24:0	Hexosylceramides	HMDB0004978
Hex-Cer d18:1/24:1	Hexosylceramide d18:1/24:1	Hexosylceramides	HMDB0010712

Hex-Cer d18:1/26:1	Hexosylceramide d18:1/26:1	Hexosylceramides	HMDB0010713
Hex-Cer d18:2/16:0	Hexosylceramide d18:2/16:0	Hexosylceramides	NA
Hex-Cer d18:2/18:0	Hexosylceramide d18:2/18:0	Hexosylceramides	NA
Hex-Cer d18:2/20:0	Hexosylceramide d18:2/20:0	Hexosylceramides	NA
Hex-Cer d18:2/22:0	Hexosylceramide d18:2/22:0	Hexosylceramides	NA
Hex-Cer d18:2/23:0	Hexosylceramide d18:2/23:0	Hexosylceramides	NA
Hex-Cer d18:2/24:0	Hexosylceramide d18:2/24:0	Hexosylceramides	NA
Cortisol	Cortisol	Hormones	HMDB0000063
Cortisone	Cortisone	Hormones	HMDB0002802
DHEAS	Dehydroepiandrosterone sulfate	Hormones	HMDB0001032
T4	Thyroxine (T4)	Hormones	HMDB0000248
3-IAA	3-Indoleacetic acid	Indoles and derivatives	HMDB0000197
3-IPA	3-Indolepropionic acid	Indoles and derivatives	HMDB0002302
5-HIAA	5-Hydroxyindoleacetic acid	Indoles and derivatives	HMDB0000763
IAG	Indolylacryloylglycine	Indoles and derivatives	HMDB0006005
Ind-SO4	Indoxyl sulfate	Indoles and derivatives	HMDB0000682
Indole-Lac	Indolelactic acid	Indoles and derivatives	HMDB0000671
Indoleacetaldehyde	Indoleacetaldehyde	Indoles and derivatives	HMDB0001190
Met-IAA	Methylindole-3-acetic acid	Indoles and derivatives	HMDB0029738
LPA 14:0	Lysophosphatidic acid 14:0	Lysophosphatidic acids	HMDB0062321
LPA 14:1	Lysophosphatidic acid 14:1	Lysophosphatidic acids	HMDB0062311
LPA 15:0	Lysophosphatidic acid 15:0	Lysophosphatidic acids	HMDB0062324
LPE 14:0	Lysophosphatidylethanolamine 14:0	Lysophosphatidylethanolamines	HMDB0011500
LPE 15:0	Lysophosphatidylethanolamine 15:0	Lysophosphatidylethanolamines	HMDB0011502
LPE 16:0	Lysophosphatidylethanolamine 16:0	Lysophosphatidylethanolamines	HMDB0011503
LPE 17:0	Lysophosphatidylethanolamine 17:0	Lysophosphatidylethanolamines	HMDB0061691
LPE 17:1	Lysophosphatidylethanolamine 17:1	Lysophosphatidylethanolamines	NA
LPE 18:0	Lysophosphatidylethanolamine 18:0	Lysophosphatidylethanolamines	HMDB0011130
LPE 18:1	Lysophosphatidylethanolamine 18:1	Lysophosphatidylethanolamines	HMDB0011506
LPE 18:2	Lysophosphatidylethanolamine 18:2	Lysophosphatidylethanolamines	HMDB0011507
LPE 18:3	Lysophosphatidylethanolamine 18:3	Lysophosphatidylethanolamines	HMDB0011508
LPE 19:0	Lysophosphatidylethanolamine 19:0	Lysophosphatidylethanolamines	HMDB0062322
LPE 20:2	Lysophosphatidylethanolamine 20:2	Lysophosphatidylethanolamines	HMDB0011513
LPE 20:3	Lysophosphatidylethanolamine 20:3	Lysophosphatidylethanolamines	HMDB0011516
LPE 20:4	Lysophosphatidylethanolamine 20:4	Lysophosphatidylethanolamines	HMDB0011517
LPE 20:5	Lysophosphatidylethanolamine 20:5	Lysophosphatidylethanolamines	HMDB0011519
LPE 22:0	Lysophosphatidylethanolamine 22:0	Lysophosphatidylethanolamines	HMDB0011520
LPE 22:4	Lysophosphatidylethanolamine 22:4	Lysophosphatidylethanolamines	HMDB0011523
LPE 22:5	Lysophosphatidylethanolamine 22:5	Lysophosphatidylethanolamines	HMDB0011494
LPE 22:6	Lysophosphatidylethanolamine 22:6	Lysophosphatidylethanolamines	HMDB0011526
LPE P-14:0	Lysophosphatidylethanolamine P-14:0	Lysophosphatidylethanolamines	NA

LPE P-16:0	Lysophosphatidylethanolamine P-16:0	Lysophosphatidylethanolamines	HMDB0011152
LPE P-17:0	Lysophosphatidylethanolamine P-17:0	Lysophosphatidylethanolamines	NA
LPE P-18:0	Lysophosphatidylethanolamine P-18:0	Lysophosphatidylethanolamines	HMDB0240598
LPE P-18:1	Lysophosphatidylethanolamine P-18:1	Lysophosphatidylethanolamines	HMDB0240599
LPE P-20:0	Lysophosphatidylethanolamine P-20:0	Lysophosphatidylethanolamines	NA
LPE P-22:0	Lysophosphatidylethanolamine P-22:0	Lysophosphatidylethanolamines	NA
LPE P-22:1	Lysophosphatidylethanolamine P-22:1	Lysophosphatidylethanolamines	NA
LPE P-22:6	Lysophosphatidylethanolamine P-22:6	Lysophosphatidylethanolamines	NA
LPS 18:0	Lysophosphatidylserine 18:0	Lysophosphatidylserines	HMDB0240606
LPS 18:1	Lysophosphatidylserine 18:1	Lysophosphatidylserines	HMDB0240603
LPS 18:2	Lysophosphatidylserine 18:2	Lysophosphatidylserines	HMDB0240604
LPS 20:1	Lysophosphatidylserine 20:1	Lysophosphatidylserines	NA
LPS 20:5	Lysophosphatidylserine 20:5	Lysophosphatidylserines	NA
LPC 14:0	Lysophosphatidylcholine 14:0	Lysophosphatidylcholines	HMDB10379
LPC 16:0	Lysophosphatidylcholine 16:0	Lysophosphatidylcholines	HMDB10382
LPC 16:1	Lysophosphatidylcholine 16:1	Lysophosphatidylcholines	HMDB0010383
LPC 17:0	Lysophosphatidylcholine 17:0	Lysophosphatidylcholines	HMDB12108
LPC 18:0	Lysophosphatidylcholine 18:0	Lysophosphatidylcholines	HMDB10384
LPC 18:1	Lysophosphatidylcholine 18:1	Lysophosphatidylcholines	HMDB02815
LPC 18:2	Lysophosphatidylcholine 18:2	Lysophosphatidylcholines	HMDB10386
LPC 20:3	Lysophosphatidylcholine 20:3	Lysophosphatidylcholines	HMDB10394
LPC 20:4	Lysophosphatidylcholine 20:4	Lysophosphatidylcholines	HMDB10395
LPC 24:0	Lysophosphatidylcholine 24:0	Lysophosphatidylcholines	HMDB10405
LPC 26:0	Lysophosphatidylcholine 26:0	Lysophosphatidylcholines	HMDB0029205
LPC 26:1	Lysophosphatidylcholine 26:1	Lysophosphatidylcholines	HMDB0029220
LPI 14:0	Lysophosphatidylinositol 14:0	Lysophosphatidylinositols	NA
LPI 14:1	Lysophosphatidylinositol 14:1	Lysophosphatidylinositols	NA
LPI 16:0	Lysophosphatidylinositol 16:0	Lysophosphatidylinositols	HMDB0061695
LPI 16:1	Lysophosphatidylinositol 16:1	Lysophosphatidylinositols	NA
LPI 17:0	Lysophosphatidylinositol 17:0	Lysophosphatidylinositols	NA
LPI 17:1	Lysophosphatidylinositol 17:1	Lysophosphatidylinositols	NA
LPI 18:0	Lysophosphatidylinositol 18:0	Lysophosphatidylinositols	HMDB0240261
LPI 18:1	Lysophosphatidylinositol 18:1	Lysophosphatidylinositols	HMDB0061693
LPI 18:2	Lysophosphatidylinositol 18:2	Lysophosphatidylinositols	HMDB0240597
LPI 18:3	Lysophosphatidylinositol 18:3	Lysophosphatidylinositols	NA
LPI 19:0	Lysophosphatidylinositol 19:0	Lysophosphatidylinositols	NA
LPI 20:1	Lysophosphatidylinositol 20:1	Lysophosphatidylinositols	NA
LPI 20:4	Lysophosphatidylinositol 20:4	Lysophosphatidylinositols	HMDB0061690
LPI 22:0	Lysophosphatidylinositol 22:0	Lysophosphatidylinositols	NA
LPI 22:1	Lysophosphatidylinositol 22:1	Lysophosphatidylinositols	NA
MG 20:1	Monoglyceride 20:1	Monoglycerides	HMDB0011543

MG 22:1	Monoglyceride 22:1	Monoglycerides	HMDB0011552
MG 22:2	Monoglyceride 22:2	Monoglycerides	HMDB0011553
MG 22:4	Monoglyceride 22:4	Monoglycerides	HMDB0011554
Adenosine	Adenosine	Nucleobase-related	HMDB0000050
AHCys	S-Adenosylhomocysteine	Nucleobase-related	HMDB0000939
Cytidine	Cytidine	Nucleobase-related	HMDB0000089
Hypoxanthine	Hypoxanthine	Nucleobase-related	HMDB0000157
N-Ac-Cytidine	N4-Acetylcytidine	Nucleobase-related	HMDB0005923
Orotic acid	Orotic acid	Nucleobase-related	HMDB0000226
Uric acid	Uric acid	Nucleobase-related	HMDB0000289
Uridine	Uridine	Nucleobase-related	HMDB0000296
Xanthine	Xanthine	Nucleobase-related	HMDB0000292
cAMP	Adenosine monophosphate, cyclic (cAMP)	Nucleotides	HMDB0000058
cGMP	Guanosine monophosphate, cyclic (cGMP)	Nucleotides	HMDB0001314
2-OH-2-Met-butyric acid	2-Hydroxy-2-methylbutyric acid	Organic acids	HMDB0001987
2-OH-Isobutyric acid	2-Hydroxyisobutyric acid	Organic acids	HMDB0000729
2,5-Furandicarboxylic acid	Furan-2,5-dicarboxylic acid	Organic acids	HMDB0004812
3-OH-Isobutyric acid	3-Hydroxyisobutyric acid	Organic acids	HMDB0000023
4-OH-HipAcid	4-Hydroxyhippuric acid	Organic acids	HMDB0013678
Argininic acid	Argininic acid	Organic acids	HMDB0003148
Bilirubin	Bilirubin	Organic acids	HMDB0000054
Biliverdin	Biliverdin	Organic acids	HMDB0001008
CMPF	3-Carboxy-4-methyl-5-propyl-2-furanpropionic acid	Organic acids	HMDB0061112
Glyceric acid	Glyceric acid	Organic acids	HMDB0006372
2-OH-Benzoic acid	2-Hydroxybenzoic acid	Phenolic acids	HMDB0001895
2-OH-Phenylacetic acid	2-Hydroxyphenylacetic acid	Phenolic acids	HMDB0000669
3,5-DiOH-Benzoic acid	3,5-Dihydroxybenzoic acid	Phenolic acids	HMDB0013677
4-OH-Phenylacetic acid	4-Hydroxyphenylacetic acid	Phenolic acids	HMDB0000020
4-OH-Phenylpyruvic acid	4-Hydroxyphenylpyruvic acid	Phenolic acids	HMDB0000707
Benzoic acid	Benzoic acid	Phenolic acids	HMDB0001870
Desamino-Tyr	Desaminotyrosine	Phenolic acids	HMDB0002199
Homovanillic acid	Homovanillic acid	Phenolic acids	HMDB0000118
HPHPA	3-(3-Hydroxyphenyl)-3-hydroxypropanoic acid	Phenolic acids	HMDB0002643
OH-Phenyl-Lac	Hydroxyphenyllactic acid	Phenolic acids	HMDB0000755
Phenyl-Lac	Phenyllactic acid	Phenolic acids	HMDB0000779
Phenylacetic acid	Phenylacetic acid	Phenolic acids	HMDB0000209
Phenylpyruvic acid	Phenylpyruvic acid	Phenolic acids	HMDB0000205
Vanillylmandelic acid	Vanillylmandelic acid	Phenolic acids	HMDB0000291
4-EPS	4-Ethylphenyl sulfate	Phenoxy compounds	HMDB0062551
Phenylglucuronide	Phenylglucuronide	Phenoxy compounds	HMDB0059806
PA 14:0_14:1	Phosphatidic acid 14:0_14:1	Phosphatidic acids	HMDB0114795

PA 16:0_18:1	Phosphatidic acid 16:0_18:1	Phosphatidic acids	HMDB0114877
PA 16:0_18:2	Phosphatidic acid 16:0_18:2	Phosphatidic acids	HMDB0114949
PA 16:0_18:3	Phosphatidic acid 16:0_18:3	Phosphatidic acids	HMDB0114836
PA 16:0_19:2	Phosphatidic acid 16:0_19:2	Phosphatidic acids	NA
PA 16:1_18:1	Phosphatidic acid 16:1_18:1	Phosphatidic acids	HMDB0114900
PA 16:1_18:2	Phosphatidic acid 16:1_18:2	Phosphatidic acids	HMDB0114856
PA 16:1_22:0	Phosphatidic acid 16:1_22:0	Phosphatidic acids	HMDB0114864
PA 16:2_18:1	Phosphatidic acid 16:2_18:1	Phosphatidic acids	NA
PA 17:0_18:1	Phosphatidic acid 17:0_18:1	Phosphatidic acids	NA
PA 17:0_18:2	Phosphatidic acid 17:0_18:2	Phosphatidic acids	NA
PA 17:1_18:1	Phosphatidic acid 17:1_18:1	Phosphatidic acids	NA
PA 17:1_18:2	Phosphatidic acid 17:1_18:2	Phosphatidic acids	NA
PA 17:2_18:1	Phosphatidic acid 17:2_18:1	Phosphatidic acids	NA
PA 18:0_18:1	Phosphatidic acid 18:0_18:1	Phosphatidic acids	HMDB0114901
PA 18:0_18:2	Phosphatidic acid 18:0_18:2	Phosphatidic acids	HMDB0114951
PA 18:0_18:3	Phosphatidic acid 18:0_18:3	Phosphatidic acids	HMDB0114878
PA 18:1_18:1	Phosphatidic acid 18:1_18:1	Phosphatidic acids	HMDB0007862
PA 18:1_18:2	Phosphatidic acid 18:1_18:2	Phosphatidic acids	HMDB0114902
PA 18:1_18:3	Phosphatidic acid 18:1_18:3	Phosphatidic acids	HMDB0114903
PA 18:1_18:4	Phosphatidic acid 18:1_18:4	Phosphatidic acids	HMDB0114905
PA 18:1_20:0	Phosphatidic acid 18:1_20:0	Phosphatidic acids	HMDB0114906
PA 18:1_20:1	Phosphatidic acid 18:1_20:1	Phosphatidic acids	HMDB0114907
PA 18:1_20:2	Phosphatidic acid 18:1_20:2	Phosphatidic acids	HMDB0115516
PA 18:1_20:3	Phosphatidic acid 18:1_20:3	Phosphatidic acids	HMDB0114908
PA 18:1_22:0	Phosphatidic acid 18:1_22:0	Phosphatidic acids	HMDB0114912
PA 18:1_22:1	Phosphatidic acid 18:1_22:1	Phosphatidic acids	HMDB0114913
PA 18:1_22:2	Phosphatidic acid 18:1_22:2	Phosphatidic acids	HMDB0114914
PA 18:1_22:3	Phosphatidic acid 18:1_22:3	Phosphatidic acids	NA
PA 18:2_18:2	Phosphatidic acid 18:2_18:2	Phosphatidic acids	HMDB0114954
PA 18:2_18:3	Phosphatidic acid 18:2_18:3	Phosphatidic acids	HMDB0114955
PA 18:2_20:0	Phosphatidic acid 18:2_20:0	Phosphatidic acids	HMDB0114958
PA 18:2_20:1	Phosphatidic acid 18:2_20:1	Phosphatidic acids	HMDB0114959
PA 18:2_20:2	Phosphatidic acid 18:2_20:2	Phosphatidic acids	HMDB0115493
PA 18:2_22:0	Phosphatidic acid 18:2_22:0	Phosphatidic acids	HMDB0114964
PA 18:2_22:1	Phosphatidic acid 18:2_22:1	Phosphatidic acids	HMDB0114965
PA 18:2_22:3	Phosphatidic acid 18:2_22:3	Phosphatidic acids	NA
PA 18:2_22:4	Phosphatidic acid 18:2_22:4	Phosphatidic acids	HMDB0114967
PA 18:3_18:3	Phosphatidic acid 18:3_18:3	Phosphatidic acids	HMDB0114982
PA 20:0_20:4	Phosphatidic acid 20:0_20:4	Phosphatidic acids	HMDB0115079
LPG 14:0	Lysophosphatidylglycerol 14:0	Lysophosphatidylglycerols	NA
LPG 14:1	Lysophosphatidylglycerol 14:1	Lysophosphatidylglycerols	NA

LPG 16:0	Lysophosphatidylglycerol 16:0	Lysophosphatidylglycerols	HMDB0240601
LPG 16:1	Lysophosphatidylglycerol 16:1	Lysophosphatidylglycerols	NA
LPG 17:1	Lysophosphatidylglycerol 17:1	Lysophosphatidylglycerols	NA
LPG 18:0	Lysophosphatidylglycerol 18:0	Lysophosphatidylglycerols	NA
LPG 18:1	Lysophosphatidylglycerol 18:1	Lysophosphatidylglycerols	HMDB0240602
LPG 18:2	Lysophosphatidylglycerol 18:2	Lysophosphatidylglycerols	HMDB0240600
LPG 20:1	Lysophosphatidylglycerol 20:1	Lysophosphatidylglycerols	NA
PE 20:0	Phosphatidylethanolamine 20:0	Phosphatidylethanolamines	NA
PE 28:0	Phosphatidylethanolamine 28:0	Phosphatidylethanolamines	HMDB0008821
PE 30:0	Phosphatidylethanolamine 30:0	Phosphatidylethanolamines	HMDB0008889
PE 30:1	Phosphatidylethanolamine 30:1	Phosphatidylethanolamines	HMDB0008857
PE 32:0	Phosphatidylethanolamine 32:0	Phosphatidylethanolamines	HMDB0008923
PE 32:1	Phosphatidylethanolamine 32:1	Phosphatidylethanolamines	HMDB0008859
PE 32:2	Phosphatidylethanolamine 32:2	Phosphatidylethanolamines	HMDB0008957
PE 33:0	Phosphatidylethanolamine 33:0	Phosphatidylethanolamines	HMDB0008988
PE 33:1	Phosphatidylethanolamine 33:1	Phosphatidylethanolamines	HMDB0008894
PE 33:2	Phosphatidylethanolamine 33:2	Phosphatidylethanolamines	HMDB0009087
PE 34:0	Phosphatidylethanolamine 34:0	Phosphatidylethanolamines	HMDB0009217
PE 34:1	Phosphatidylethanolamine 34:1	Phosphatidylethanolamines	HMDB0008927
PE 34:2	Phosphatidylethanolamine 34:2	Phosphatidylethanolamines	HMDB0008928
PE 34:3	Phosphatidylethanolamine 34:3	Phosphatidylethanolamines	HMDB0008930
PE 34:4	Phosphatidylethanolamine 34:4	Phosphatidylethanolamines	HMDB0008870
PE 35:1	Phosphatidylethanolamine 35:1	Phosphatidylethanolamines	HMDB0008900
PE 35:2	Phosphatidylethanolamine 35:2	Phosphatidylethanolamines	HMDB0008901
PE 35:3	Phosphatidylethanolamine 35:3	Phosphatidylethanolamines	HMDB0008903
PE 36:0	Phosphatidylethanolamine 36:0	Phosphatidylethanolamines	HMDB0008991
PE 36:1	Phosphatidylethanolamine 36:1	Phosphatidylethanolamines	HMDB0008993
PE 36:2	Phosphatidylethanolamine 36:2	Phosphatidylethanolamines	HMDB0008994
PE 36:3	Phosphatidylethanolamine 36:3	Phosphatidylethanolamines	HMDB0009060
PE 36:4	Phosphatidylethanolamine 36:4	Phosphatidylethanolamines	HMDB0008937
PE 36:5	Phosphatidylethanolamine 36:5	Phosphatidylethanolamines	HMDB0008877
PE 36:6	Phosphatidylethanolamine 36:6	Phosphatidylethanolamines	HMDB0008972
PE 38:0	Phosphatidylethanolamine 38:0	Phosphatidylethanolamines	HMDB0008998
PE 38:1	Phosphatidylethanolamine 38:1	Phosphatidylethanolamines	HMDB0009224
PE 38:2	Phosphatidylethanolamine 38:2	Phosphatidylethanolamines	HMDB0009000
PE 38:3	Phosphatidylethanolamine 38:3	Phosphatidylethanolamines	HMDB0008975
PE 38:4	Phosphatidylethanolamine 38:4	Phosphatidylethanolamines	HMDB0009228
PE 38:5	Phosphatidylethanolamine 38:5	Phosphatidylethanolamines	HMDB0008976
PE 38:6	Phosphatidylethanolamine 38:6	Phosphatidylethanolamines	HMDB0008946
PE 38:7	Phosphatidylethanolamine 38:7	Phosphatidylethanolamines	HMDB0009104
PE 40:1	Phosphatidylethanolamine 40:1	Phosphatidylethanolamines	HMDB0009007

PE 40:3	Phosphatidylethanolamine 40:3	Phosphatidylethanolamines	HMDB0009074
PE 40:4	Phosphatidylethanolamine 40:4	Phosphatidylethanolamines	HMDB0009107
PE 40:5	Phosphatidylethanolamine 40:5	Phosphatidylethanolamines	HMDB0009075
PE 40:6	Phosphatidylethanolamine 40:6	Phosphatidylethanolamines	HMDB0009012
PE 40:7	Phosphatidylethanolamine 40:7	Phosphatidylethanolamines	HMDB0009141
PE 40:8	Phosphatidylethanolamine 40:8	Phosphatidylethanolamines	HMDB0009207
PE 42:7	Phosphatidylethanolamine 42:7	Phosphatidylethanolamines	HMDB0009276
PE 42:8	Phosphatidylethanolamine 42:8	Phosphatidylethanolamines	HMDB0009309
PE 44:6	Phosphatidylethanolamine 44:6	Phosphatidylethanolamines	HMDB0009507
PE 44:7	Phosphatidylethanolamine 44:7	Phosphatidylethanolamines	HMDB0009700
PE 44:11	Phosphatidylethanolamine 44:11	Phosphatidylethanolamines	HMDB0009639
PE 44:12	Phosphatidylethanolamine 44:12	Phosphatidylethanolamines	HMDB0009705
PE P-16:0/16:0	Phosphatidylethanolamine P-16:0/16:0	Phosphatidylethanolamines	HMDB0011158
PE P-16:0/18:1	Phosphatidylethanolamine P-16:0/18:1	Phosphatidylethanolamines	HMDB0011342
PE P-16:0/18:2	Phosphatidylethanolamine P-16:0/18:2	Phosphatidylethanolamines	HMDB0011343
PE P-16:0/18:3	Phosphatidylethanolamine P-16:0/18:3	Phosphatidylethanolamines	HMDB0009180
PE P-16:0/20:3	Phosphatidylethanolamine P-16:0/20:3	Phosphatidylethanolamines	HMDB0011351
PE P-16:0/20:4	Phosphatidylethanolamine P-16:0/20:4	Phosphatidylethanolamines	HMDB0011352
PE P-16:0/20:5	Phosphatidylethanolamine P-16:0/20:5	Phosphatidylethanolamines	HMDB0011354
PE P-16:0/22:4	Phosphatidylethanolamine P-16:0/22:4	Phosphatidylethanolamines	HMDB0011358
PE P-16:0/22:5	Phosphatidylethanolamine P-16:0/22:5	Phosphatidylethanolamines	HMDB0009675
PE P-16:0/22:6	Phosphatidylethanolamine P-16:0/22:6	Phosphatidylethanolamines	HMDB0009708
PE P-18:0/14:0	Phosphatidylethanolamine P-18:0/14:0	Phosphatidylethanolamines	HMDB0011368
PE P-18:0/16:0	Phosphatidylethanolamine P-18:0/16:0	Phosphatidylethanolamines	HMDB0011371
PE P-18:0/16:1	Phosphatidylethanolamine P-18:0/16:1	Phosphatidylethanolamines	HMDB0011372
PE P-18:0/18:1	Phosphatidylethanolamine P-18:0/18:1	Phosphatidylethanolamines	HMDB0009049
PE P-18:0/18:2	Phosphatidylethanolamine P-18:0/18:2	Phosphatidylethanolamines	HMDB0011376
PE P-18:0/18:3	Phosphatidylethanolamine P-18:0/18:3	Phosphatidylethanolamines	HMDB0011377
PE P-18:0/20:1	Phosphatidylethanolamine P-18:0/20:1	Phosphatidylethanolamines	HMDB0011381
PE P-18:0/20:2	Phosphatidylethanolamine P-18:0/20:2	Phosphatidylethanolamines	HMDB0011382
PE P-18:0/20:3	Phosphatidylethanolamine P-18:0/20:3	Phosphatidylethanolamines	HMDB0011384
PE P-18:0/20:4	Phosphatidylethanolamine P-18:0/20:4	Phosphatidylethanolamines	HMDB0005779
PE P-18:0/20:5	Phosphatidylethanolamine P-18:0/20:5	Phosphatidylethanolamines	HMDB0011387
PE P-18:0/22:3	Phosphatidylethanolamine P-18:0/22:3	Phosphatidylethanolamines	NA
PE P-18:0/22:4	Phosphatidylethanolamine P-18:0/22:4	Phosphatidylethanolamines	HMDB0011391
PE P-18:0/22:5	Phosphatidylethanolamine P-18:0/22:5	Phosphatidylethanolamines	HMDB0011392
PE P-18:0/22:6	Phosphatidylethanolamine P-18:0/22:6	Phosphatidylethanolamines	HMDB0009709
PE P-18:1/18:1	Phosphatidylethanolamine P-18:1/18:1	Phosphatidylethanolamines	HMDB0009084
PE P-18:1/18:2	Phosphatidylethanolamine P-18:1/18:2	Phosphatidylethanolamines	HMDB0011409
PE P-18:1/20:4	Phosphatidylethanolamine P-18:1/20:4	Phosphatidylethanolamines	HMDB0011452
PE P-18:1/20:5	Phosphatidylethanolamine P-18:1/20:5	Phosphatidylethanolamines	HMDB0011453

PE P-18:1/22:6	Phosphatidylethanolamine P-18:1/22:6	Phosphatidylethanolamines	HMDB0011460
PE P-20:0/18:2	Phosphatidylethanolamine P-20:0/18:2	Phosphatidylethanolamines	NA
PE P-20:0/20:4	Phosphatidylethanolamine P-20:0/20:4	Phosphatidylethanolamines	NA
PE P-20:0/20:5	Phosphatidylethanolamine P-20:0/20:5	Phosphatidylethanolamines	NA
PI 14:0_18:1	Phosphatidyl-inositol 14:0_18:1	Phosphatidylinositols	NA
PI 14:0_18:2	Phosphatidyl-inositol 14:0_18:2	Phosphatidylinositols	NA
PI 15:0_16:0	Phosphatidyl-inositol 15:0_16:0	Phosphatidylinositols	NA
PI 15:1_16:0	Phosphatidyl-inositol 15:1_16:0	Phosphatidylinositols	NA
PI 16:0_16:0	Phosphatidyl-inositol 16:0_16:0	Phosphatidylinositols	HMDB0009778
PI 16:0_17:0	Phosphatidyl-inositol 16:0_17:0	Phosphatidylinositols	NA
PI 16:0_17:1	Phosphatidyl-inositol 16:0_17:1	Phosphatidylinositols	NA
PI 16:0_17:2	Phosphatidyl-inositol 16:0_17:2	Phosphatidylinositols	NA
PI 16:0_18:1	Phosphatidyl-inositol 16:0_18:1	Phosphatidylinositols	HMDB0009782
PI 16:0_18:2	Phosphatidyl-inositol 16:0_18:2	Phosphatidylinositols	HMDB0009784
PI 16:0_18:3	Phosphatidyl-inositol 16:0_18:3	Phosphatidylinositols	NA
PI 16:0_20:0	Phosphatidyl-inositol 16:0_20:0	Phosphatidylinositols	HMDB0009785
PI 16:0_20:3	Phosphatidyl-inositol 16:0_20:3	Phosphatidylinositols	HMDB0009886
PI 16:0_20:4	Phosphatidyl-inositol 16:0_20:4	Phosphatidylinositols	HMDB0009893
PI 16:0_22:1	Phosphatidyl-inositol 16:0_22:1	Phosphatidylinositols	NA
PI 16:1_18:0	Phosphatidyl-inositol 16:1_18:0	Phosphatidylinositols	HMDB0009799
PI 16:1_18:1	Phosphatidyl-inositol 16:1_18:1	Phosphatidylinositols	HMDB0009835
PI 16:1_18:2	Phosphatidyl-inositol 16:1_18:2	Phosphatidylinositols	NA
PI 17:0_18:1	Phosphatidyl-inositol 17:0_18:1	Phosphatidylinositols	NA
PI 17:1_18:1	Phosphatidyl-inositol 17:1_18:1	Phosphatidylinositols	NA
PI 17:1_18:2	Phosphatidyl-inositol 17:1_18:2	Phosphatidylinositols	NA
PI 18:0_18:0	Phosphatidyl-inositol 18:0_18:0	Phosphatidylinositols	HMDB0009808
PI 18:0_18:1	Phosphatidyl-inositol 18:0_18:1	Phosphatidylinositols	HMDB0240667
PI 18:0_18:2	Phosphatidyl-inositol 18:0_18:2	Phosphatidylinositols	HMDB0009847
PI 18:0_18:3	Phosphatidyl-inositol 18:0_18:3	Phosphatidylinositols	HMDB0009810
PI 18:0_20:0	Phosphatidyl-inositol 18:0_20:0	Phosphatidylinositols	NA
PI 18:0_20:3	Phosphatidyl-inositol 18:0_20:3	Phosphatidylinositols	HMDB0009887
PI 18:0_20:4	Phosphatidyl-inositol 18:0_20:4	Phosphatidylinositols	HMDB0009815
PI 18:0_22:0	Phosphatidyl-inositol 18:0_22:0	Phosphatidylinositols	NA
PI 18:1_18:1	Phosphatidyl-inositol 18:1_18:1	Phosphatidylinositols	HMDB0009824
PI 18:1_18:2	Phosphatidyl-inositol 18:1_18:2	Phosphatidylinositols	HMDB0009849
PI 18:1_18:3	Phosphatidyl-inositol 18:1_18:3	Phosphatidylinositols	HMDB0009839
PI 18:1_20:0	Phosphatidyl-inositol 18:1_20:0	Phosphatidylinositols	NA
PI 18:1_20:1	Phosphatidyl-inositol 18:1_20:1	Phosphatidylinositols	HMDB0009871
PI 18:1_20:2	Phosphatidyl-inositol 18:1_20:2	Phosphatidylinositols	NA
PI 18:1_20:3	Phosphatidyl-inositol 18:1_20:3	Phosphatidylinositols	HMDB0009843
PI 18:1_20:4	Phosphatidyl-inositol 18:1_20:4	Phosphatidylinositols	HMDB0009896

PI 18:1_20:5	Phosphatidyl-inositol 18:1_20:5	Phosphatidylinositols	NA
PI 18:1_22:0	Phosphatidyl-inositol 18:1_22:0	Phosphatidylinositols	NA
PI 18:1_22:1	Phosphatidyl-inositol 18:1_22:1	Phosphatidylinositols	NA
PI 18:1_22:2	Phosphatidyl-inositol 18:1_22:2	Phosphatidylinositols	NA
PI 18:1_22:3	Phosphatidyl-inositol 18:1_22:3	Phosphatidylinositols	NA
PI 18:1_22:4	Phosphatidyl-inositol 18:1_22:4	Phosphatidylinositols	NA
PI 18:1_22:5	Phosphatidyl-inositol 18:1_22:5	Phosphatidylinositols	NA
PI 18:1_22:6	Phosphatidyl-inositol 18:1_22:6	Phosphatidylinositols	NA
PI 18:2_18:3	Phosphatidyl-inositol 18:2_18:3	Phosphatidylinositols	NA
PI 18:2_20:0	Phosphatidyl-inositol 18:2_20:0	Phosphatidylinositols	HMDB0009851
PI 18:2_20:1	Phosphatidyl-inositol 18:2_20:1	Phosphatidylinositols	HMDB0009852
PI 18:2_20:4	Phosphatidyl-inositol 18:2_20:4	Phosphatidylinositols	NA
PI 18:2_20:5	Phosphatidyl-inositol 18:2_20:5	Phosphatidylinositols	NA
PI 18:2_22:0	Phosphatidyl-inositol 18:2_22:0	Phosphatidylinositols	NA
PI 18:2_22:1	Phosphatidyl-inositol 18:2_22:1	Phosphatidylinositols	NA
PI 18:2_22:6	Phosphatidyl-inositol 18:2_22:6	Phosphatidylinositols	NA
PS 32:0	Phosphatidyl-serine 32:0	Phosphatidylserines	HMDB0000614
PS 34:1	Phosphatidyl-serine 34:1	Phosphatidylserines	HMDB0112267
PS 34:2	Phosphatidyl-serine 34:2	Phosphatidylserines	HMDB0112296
PS 36:1	Phosphatidyl-serine 36:1	Phosphatidylserines	HMDB0010163
PS 36:2	Phosphatidyl-serine 36:2	Phosphatidylserines	HMDB0012390
PS 36:3	Phosphatidyl-serine 36:3	Phosphatidylserines	HMDB0112315
PS 36:4	Phosphatidyl-serine 36:4	Phosphatidylserines	HMDB0012402
PS 36:5	Phosphatidyl-serine 36:5	Phosphatidylserines	HMDB0112316
PS 38:4	Phosphatidyl-serine 38:4	Phosphatidylserines	HMDB0012383
PS 38:5	Phosphatidyl-serine 38:5	Phosphatidylserines	HMDB0112367
PS 38:6	Phosphatidyl-serine 38:6	Phosphatidylserines	HMDB0012362
PS 38:7	Phosphatidyl-serine 38:7	Phosphatidylserines	HMDB0012373
PS 40:4	Phosphatidyl-serine 40:4	Phosphatidylserines	HMDB0112437
PS 40:5	Phosphatidyl-serine 40:5	Phosphatidylserines	HMDB0112420
PS 40:6	Phosphatidyl-serine 40:6	Phosphatidylserines	HMDB0010167
PS 40:7	Phosphatidyl-serine 40:7	Phosphatidylserines	HMDB0012395
PS 40:8	Phosphatidyl-serine 40:8	Phosphatidylserines	HMDB0112506
PC 24:0	Phosphatidylcholine 24:0	Phosphatidylcholines	NA
PC 28:1	Phosphatidylcholine 28:1	Phosphatidylcholines	HMDB0007867
PC 30:0	Phosphatidylcholine 30:0	Phosphatidylcholines	HMDB0007934
PC 32:0	Phosphatidylcholine 32:0	Phosphatidylcholines	HMDB00564
PC 32:1	Phosphatidylcholine 32:1	Phosphatidylcholines	HMDB0007872
PC 32:2	Phosphatidylcholine 32:2	Phosphatidylcholines	HMDB0008002
PC 32:3	Phosphatidylcholine 32:3	Phosphatidylcholines	HMDB0007876
PC 34:1	Phosphatidylcholine 34:1	Phosphatidylcholines	HMDB0007971

PC 34:2	Phosphatidylcholine 34:2	Phosphatidylcholines	HMDB07973
PC 34:3	Phosphatidylcholine 34:3	Phosphatidylcholines	HMDB08006
PC 34:4	Phosphatidylcholine 34:4	Phosphatidylcholines	HMDB0007883
PC 36:0	Phosphatidylcholine 36:0	Phosphatidylcholines	HMDB0008265
PC 36:1	Phosphatidylcholine 36:1	Phosphatidylcholines	HMDB0008037
PC 36:2	Phosphatidylcholine 36:2	Phosphatidylcholines	HMDB0008039
PC 36:3	Phosphatidylcholine 36:3	Phosphatidylcholines	HMDB0007980
PC 36:4	Phosphatidylcholine 36:4	Phosphatidylcholines	HMDB0008042
PC 36:5	Phosphatidylcholine 36:5	Phosphatidylcholines	HMDB0007984
PC 36:6	Phosphatidylcholine 36:6	Phosphatidylcholines	HMDB0007892
PC 38:0	Phosphatidylcholine 38:0	Phosphatidylcholines	HMDB0007893
PC 38:3	Phosphatidylcholine 38:3	Phosphatidylcholines	HMDB0008046
PC 38:4	Phosphatidylcholine 38:4	Phosphatidylcholines	HMDB0007988
PC 38:5	Phosphatidylcholine 38:5	Phosphatidylcholines	HMDB0007989
PC 38:6	Phosphatidylcholine 38:6	Phosphatidylcholines	HMDB0008083
PC 40:2	Phosphatidylcholine 40:2	Phosphatidylcholines	HMDB0008276
PC 40:3	Phosphatidylcholine 40:3	Phosphatidylcholines	HMDB0008119
PC 40:4	Phosphatidylcholine 40:4	Phosphatidylcholines	HMDB0008054
PC 40:5	Phosphatidylcholine 40:5	Phosphatidylcholines	HMDB0008055
PC 40:6	Phosphatidylcholine 40:6	Phosphatidylcholines	HMDB0008057
PC 42:0	Phosphatidylcholine 42:0	Phosphatidylcholines	HMDB0008537
PC 42:1	Phosphatidylcholine 42:1	Phosphatidylcholines	HMDB0008059
PC 42:2	Phosphatidylcholine 42:2	Phosphatidylcholines	HMDB0008157
PC 42:4	Phosphatidylcholine 42:4	Phosphatidylcholines	HMDB0008285
PC 42:5	Phosphatidylcholine 42:5	Phosphatidylcholines	HMDB0008287
PC 42:6	Phosphatidylcholine 42:6	Phosphatidylcholines	HMDB0008288
PC O-28:0	Phosphatidylcholine O-28:0	Phosphatidylcholines	NA
PC O-28:1	Phosphatidylcholine O-28:1	Phosphatidylcholines	NA
PC O-30:0	Phosphatidylcholine O-30:0	Phosphatidylcholines	HMDB0013341
PC O-30:1	Phosphatidylcholine O-30:1	Phosphatidylcholines	HMDB0013402
PC O-30:2	Phosphatidylcholine O-30:2	Phosphatidylcholines	HMDB0013410
PC O-32:1	Phosphatidylcholine O-32:1	Phosphatidylcholines	HMDB0013404
PC O-32:2	Phosphatidylcholine O-32:2	Phosphatidylcholines	HMDB0013411
PC O-34:0	Phosphatidylcholine O-34:0	Phosphatidylcholines	HMDB0013405
PC O-34:1	Phosphatidylcholine O-34:1	Phosphatidylcholines	HMDB0013426
PC O-34:2	Phosphatidylcholine O-34:2	Phosphatidylcholines	HMDB0011151
PC O-34:3	Phosphatidylcholine O-34:3	Phosphatidylcholines	HMDB0013413
PC O-36:0	Phosphatidylcholine O-36:0	Phosphatidylcholines	HMDB13406
PC O-36:1	Phosphatidylcholine O-36:1	Phosphatidylcholines	HMDB0013427
PC O-36:2	Phosphatidylcholine O-36:2	Phosphatidylcholines	HMDB0013418
PC O-36:3	Phosphatidylcholine O-36:3	Phosphatidylcholines	HMDB0013429

PC O-36:4	Phosphatidylcholine O-36:4	Phosphatidylcholines	HMDB0013435
PC O-36:5	Phosphatidylcholine O-36:5	Phosphatidylcholines	HMDB0013415
PC O-38:0	Phosphatidylcholine O-38:0	Phosphatidylcholines	HMDB0013408
PC O-38:1	Phosphatidylcholine O-38:1	Phosphatidylcholines	HMDB0013416
PC O-38:2	Phosphatidylcholine O-38:2	Phosphatidylcholines	HMDB0013431
PC O-38:3	Phosphatidylcholine O-38:3	Phosphatidylcholines	HMDB0013439
PC O-38:4	Phosphatidylcholine O-38:4	Phosphatidylcholines	HMDB0013420
PC O-38:5	Phosphatidylcholine O-38:5	Phosphatidylcholines	HMDB11253
PC O-38:6	Phosphatidylcholine O-38:6	Phosphatidylcholines	HMDB0013409
PC O-40:1	Phosphatidylcholine O-40:1	Phosphatidylcholines	HMDB0013433
PC O-40:2	Phosphatidylcholine O-40:2	Phosphatidylcholines	HMDB0013437
PC O-40:3	Phosphatidylcholine O-40:3	Phosphatidylcholines	HMDB0013445
PC O-40:4	Phosphatidylcholine O-40:4	Phosphatidylcholines	HMDB0013442
PC O-40:5	Phosphatidylcholine O-40:5	Phosphatidylcholines	HMDB0013444
PC O-40:6	Phosphatidylcholine O-40:6	Phosphatidylcholines	HMDB0013422
PC O-42:1	Phosphatidylcholine O-42:1	Phosphatidylcholines	HMDB0013434
PC O-42:2	Phosphatidylcholine O-42:2	Phosphatidylcholines	HMDB0013438
PC O-42:3	Phosphatidylcholine O-42:3	Phosphatidylcholines	HMDB0013458
PC O-42:4	Phosphatidylcholine O-42:4	Phosphatidylcholines	HMDB0013448
PC O-42:5	Phosphatidylcholine O-42:5	Phosphatidylcholines	HMDB0013451
PC O-44:3	Phosphatidylcholine O-44:3	Phosphatidylcholines	HMDB0013449
PC O-44:4	Phosphatidylcholine O-44:4	Phosphatidylcholines	HMDB0013455
PC O-44:5	Phosphatidylcholine O-44:5	Phosphatidylcholines	HMDB0013456
PC O-44:6	Phosphatidylcholine O-44:6	Phosphatidylcholines	HMDB0013450
PG 14:0_16:0	Phosphatidylglycerol 14:0_16:0	Phosphatidylglycerols	NA
PG 15:0_18:1	Phosphatidylglycerol 15:0_18:1	Phosphatidylglycerols	NA
PG 16:0_16:0	Phosphatidylglycerol 16:0_16:0	Phosphatidylglycerols	HMDB0010570
PG 16:0_16:1	Phosphatidylglycerol 16:0_16:1	Phosphatidylglycerols	HMDB0010585
PG 16:0_18:1	Phosphatidylglycerol 16:0_18:1	Phosphatidylglycerols	HMDB10574
PG 16:0_18:2	Phosphatidylglycerol 16:0_18:2	Phosphatidylglycerols	HMDB0010645
PG 16:0_18:3	Phosphatidylglycerol 16:0_18:3	Phosphatidylglycerols	HMDB0010660
PG 16:0_19:1	Phosphatidylglycerol 16:0_19:1	Phosphatidylglycerols	NA
PG 16:0_20:3	Phosphatidylglycerol 16:0_20:3	Phosphatidylglycerols	HMDB0010579
PG 16:0_20:4	Phosphatidylglycerol 16:0_20:4	Phosphatidylglycerols	HMDB0010580
PG 16:0_20:5	Phosphatidylglycerol 16:0_20:5	Phosphatidylglycerols	NA
PG 16:0_22:1	Phosphatidylglycerol 16:0_22:1	Phosphatidylglycerols	NA
PG 16:0_22:2	Phosphatidylglycerol 16:0_22:2	Phosphatidylglycerols	NA
PG 16:1_16:1	Phosphatidylglycerol 16:1_16:1	Phosphatidylglycerols	HMDB0010586
PG 16:1_18:0	Phosphatidylglycerol 16:1_18:0	Phosphatidylglycerols	HMDB0010587
PG 16:1_18:1	Phosphatidylglycerol 16:1_18:1	Phosphatidylglycerols	HMDB0010631
PG 16:1_18:2	Phosphatidylglycerol 16:1_18:2	Phosphatidylglycerols	HMDB0010646

PG 16:1_20:4	Phosphatidylglycerol 16:1_20:4	Phosphatidylglycerols	HMDB0116608
PG 16:1_22:1	Phosphatidylglycerol 16:1_22:1	Phosphatidylglycerols	NA
PG 16:2_18:1	Phosphatidylglycerol 16:2_18:1	Phosphatidylglycerols	NA
PG 16:2_18:2	Phosphatidylglycerol 16:2_18:2	Phosphatidylglycerols	NA
PG 16:3_18:1	Phosphatidylglycerol 16:3_18:1	Phosphatidylglycerols	NA
PG 17:0_18:1	Phosphatidylglycerol 17:0_18:1	Phosphatidylglycerols	NA
PG 17:0_18:2	Phosphatidylglycerol 17:0_18:2	Phosphatidylglycerols	NA
PG 17:1_18:1	Phosphatidylglycerol 17:1_18:1	Phosphatidylglycerols	NA
PG 18:0_18:1	Phosphatidylglycerol 18:0_18:1	Phosphatidylglycerols	HMDB0010617
PG 18:0_18:2	Phosphatidylglycerol 18:0_18:2	Phosphatidylglycerols	HMDB0010647
PG 18:0_18:3	Phosphatidylglycerol 18:0_18:3	Phosphatidylglycerols	HMDB0010606
PG 18:0_22:1	Phosphatidylglycerol 18:0_22:1	Phosphatidylglycerols	NA
PG 18:1_18:1	Phosphatidylglycerol 18:1_18:1	Phosphatidylglycerols	HMDB0010634
PG 18:1_18:2	Phosphatidylglycerol 18:1_18:2	Phosphatidylglycerols	HMDB0010649
PG 18:1_18:3	Phosphatidylglycerol 18:1_18:3	Phosphatidylglycerols	HMDB0010636
PG 18:1_20:0	Phosphatidylglycerol 18:1_20:0	Phosphatidylglycerols	NA
PG 18:1_20:1	Phosphatidylglycerol 18:1_20:1	Phosphatidylglycerols	NA
PG 18:1_20:2	Phosphatidylglycerol 18:1_20:2	Phosphatidylglycerols	NA
PG 18:1_20:3	Phosphatidylglycerol 18:1_20:3	Phosphatidylglycerols	HMDB0010639
PG 18:1_20:4	Phosphatidylglycerol 18:1_20:4	Phosphatidylglycerols	HMDB0116610
PG 18:1_20:5	Phosphatidylglycerol 18:1_20:5	Phosphatidylglycerols	NA
PG 18:1_22:0	Phosphatidylglycerol 18:1_22:0	Phosphatidylglycerols	NA
PG 18:1_22:1	Phosphatidylglycerol 18:1_22:1	Phosphatidylglycerols	NA
PG 18:1_22:2	Phosphatidylglycerol 18:1_22:2	Phosphatidylglycerols	NA
PG 18:1_22:3	Phosphatidylglycerol 18:1_22:3	Phosphatidylglycerols	NA
PG 18:1_22:4	Phosphatidylglycerol 18:1_22:4	Phosphatidylglycerols	HMDB0010626
PG 18:1_22:5	Phosphatidylglycerol 18:1_22:5	Phosphatidylglycerols	HMDB0116616
PG 18:2_18:2	Phosphatidylglycerol 18:2_18:2	Phosphatidylglycerols	HMDB0010650
PG 18:2_18:3	Phosphatidylglycerol 18:2_18:3	Phosphatidylglycerols	HMDB0010651
PG 18:2_18:4	Phosphatidylglycerol 18:2_18:4	Phosphatidylglycerols	NA
PG 18:2_20:0	Phosphatidylglycerol 18:2_20:0	Phosphatidylglycerols	NA
PG 18:2_20:3	Phosphatidylglycerol 18:2_20:3	Phosphatidylglycerols	HMDB0010654
PG 18:2_20:4	Phosphatidylglycerol 18:2_20:4	Phosphatidylglycerols	HMDB0116584
PG 18:2_20:5	Phosphatidylglycerol 18:2_20:5	Phosphatidylglycerols	NA
PG 18:2_22:0	Phosphatidylglycerol 18:2_22:0	Phosphatidylglycerols	NA
PG 18:2_22:1	Phosphatidylglycerol 18:2_22:1	Phosphatidylglycerols	NA
PG 18:2_22:3	Phosphatidylglycerol 18:2_22:3	Phosphatidylglycerols	NA
PG 18:2_22:4	Phosphatidylglycerol 18:2_22:4	Phosphatidylglycerols	HMDB0010656
PG 20:3_20:4	Phosphatidylglycerol 20:3_20:4	Phosphatidylglycerols	HMDB0116588
PG 20:4_20:4	Phosphatidylglycerol 20:4_20:4	Phosphatidylglycerols	HMDB0116596
PG 20:4_22:1	Phosphatidylglycerol 20:4_22:1	Phosphatidylglycerols	NA

PG 20:4_22:3	Phosphatidylglycerol 20:4_22:3	Phosphatidylglycerols	NA
PG 20:4_22:4	Phosphatidylglycerol 20:4_22:4	Phosphatidylglycerols	NA
PG 22:4_22:6	Phosphatidylglycerol 22:4_22:6	Phosphatidylglycerols	NA
PG 22:5_22:6	Phosphatidylglycerol 22:5_22:6	Phosphatidylglycerols	HMDB0116635
PG 22:6_22:6	Phosphatidylglycerol 22:6_22:6	Phosphatidylglycerols	HMDB0116605
N-Ac-Putrescine	N-Acetylputrescine	Polyamines	HMDB0002064
N1-Ac-Spermidine	N1-Acetylspermidine	Polyamines	HMDB0001276
N1,N8-Di-Ac-Spermidine	N1,N8-Diacetylspermidine	Polyamines	HMDB0041947
N1,N12-Di-Ac-Spermine	N1,N12-Diacetylspermine	Polyamines	HMDB0002172
N8-Ac-Spermidine	N8-Acetylspermidine	Polyamines	HMDB0002189
Isonicotinic acid	Isonicotinic acid	Pyridinecarboxylic acids	HMDB0060665
Kynurenic acid	Kynurenic acid	Pyridinecarboxylic acids	HMDB0000715
Picolinic acid	Picolinic acid	Pyridinecarboxylic acids	HMDB0002243
Quinaldic acid	Quinaldic acid	Pyridinecarboxylic acids	HMDB0000842
Quinolinic acid	Quinolinic acid	Pyridinecarboxylic acids	HMDB0000232
Xanthurenic acid	Xanthurenic acid	Pyridinecarboxylic acids	HMDB0000881
SPBP d14:0	C14 Sphinganine-1-phosphate	Sphinganine and sphingosine phosphates	NA
SPBP d14:1	C14 Sphingosine-1-phosphate	Sphinganine and sphingosine phosphates	NA
SPBP d16:1	C16 Sphingosine-1-phosphate	Sphinganine and sphingosine phosphates	HMDB0060061
SPBP d17:0	C17 Sphinganine-1-phosphate	Sphinganine and sphingosine phosphates	NA
SPBP d17:1	C17 Sphingosine-1-phosphate	Sphinganine and sphingosine phosphates	NA
SPBP d18:1	C18 Sphingosine-1-phosphate	Sphinganine and sphingosine phosphates	HMDB0000277
SPB d14:0	C14 Sphinganine	Sphinganines and sphingosines	NA
SPB d16:0	C16 Sphinganine	Sphinganines and sphingosines	NA
SPB d17:0	C17 Sphinganine	Sphinganines and sphingosines	NA
SPB d18:0	C18 Sphinganine	Sphinganines and sphingosines	HMDB0000269
SPB d18:1	C18 Sphingosine	Sphinganines and sphingosines	HMDB0000252
SM 33:1	Sphingomyelin 33:1	Sphingomyelins	HMDB0240608
SM 34:1	Sphingomyelin 34:1	Sphingomyelins	HMDB0061712
SM 34:2	Sphingomyelin 34:2	Sphingomyelins	NA
SM 35:1	Sphingomyelin 35:1	Sphingomyelins	HMDB0240609
SM 36:1	Sphingomyelin 36:1	Sphingomyelins	HMDB01348
SM 36:2	Sphingomyelin 36:2	Sphingomyelins	HMDB0012100
SM 38:3	Sphingomyelin 38:3	Sphingomyelins	NA
SM 41:1	Sphingomyelin 41:1	Sphingomyelins	HMDB0012105
SM 41:2	Sphingomyelin 41:2	Sphingomyelins	HMDB0240614
SM 42:1	Sphingomyelin 42:1	Sphingomyelins	HMDB0011697
SM 42:2	Sphingomyelin 42:2	Sphingomyelins	HMDB0012107
SM 43:1	Sphingomyelin 43:1	Sphingomyelins	HMDB0240671
SM 44:1	Sphingomyelin 44:1	Sphingomyelins	HMDB0011698
SM 44:2	Sphingomyelin 44:2	Sphingomyelins	HMDB0013461

3-Deoxyglucosone	3-Deoxyglucosone	Sugars	HMDB0245856
Fructose	Fructose	Sugars	HMDB0000660
Glucose	Glucose	Sugars	HMDB0000122
Mannose	Mannose	Sugars	HMDB0000169
NeuAc	Acetylneuraminic acid	Sugars	HMDB0000230
Ribose	Ribose	Sugars	HMDB0000283
Threonic acid	Threonic acid	Sugars	HMDB0000943
AconAcid	Aconitic acid	Tricarboxylic acids	HMDB0000958
Citric acid	Citric acid	Tricarboxylic acids	HMDB0000094
Isocitric acid	Isocitric acid	Tricarboxylic acids	HMDB0000193
TG 14:0_32:2	Triacylglyceride 14:0_32:2	Triglycerides	HMDB0042281
TG 14:0_34:1	Triacylglyceride 14:0_34:1	Triglycerides	HMDB0042275
TG 14:0_34:2	Triacylglyceride 14:0_34:2	Triglycerides	HMDB0042282
TG 14:0_34:3	Triacylglyceride 14:0_34:3	Triglycerides	HMDB0042316
TG 14:0_35:1	Triacylglyceride 14:0_35:1	Triglycerides	HMDB0042102
TG 14:0_35:2	Triacylglyceride 14:0_35:2	Triglycerides	HMDB0042108
TG 14:0_36:2	Triacylglyceride 14:0_36:2	Triglycerides	HMDB0062639
TG 14:0_36:3	Triacylglyceride 14:0_36:3	Triglycerides	HMDB0042167
TG 14:0_36:4	Triacylglyceride 14:0_36:4	Triglycerides	HMDB0042384
TG 14:0_38:4	Triacylglyceride 14:0_38:4	Triglycerides	HMDB0042176
TG 14:0_38:5	Triacylglyceride 14:0_38:5	Triglycerides	HMDB0042792
TG 14:0_40:5	Triacylglyceride 14:0_40:5	Triglycerides	NA
TG 16:0_28:1	Triacylglyceride 16:0_28:1	Triglycerides	HMDB0042273
TG 16:0_28:2	Triacylglyceride 16:0_28:2	Triglycerides	HMDB0044030
TG 16:0_30:2	Triacylglyceride 16:0_30:2	Triglycerides	HMDB0047771
TG 16:0_32:0	Triacylglyceride 16:0_32:0	Triglycerides	HMDB0005356
TG 16:0_32:1	Triacylglyceride 16:0_32:1	Triglycerides	HMDB05359
TG 16:0_32:2	Triacylglyceride 16:0_32:2	Triglycerides	HMDB05376
TG 16:0_32:3	Triacylglyceride 16:0_32:3	Triglycerides	HMDB0047778
TG 16:0_33:1	Triacylglyceride 16:0_33:1	Triglycerides	HMDB0043026
TG 16:0_33:2	Triacylglyceride 16:0_33:2	Triglycerides	HMDB0043032
TG 16:0_34:1	Triacylglyceride 16:0_34:1	Triglycerides	HMDB05374
TG 16:0_34:2	Triacylglyceride 16:0_34:2	Triglycerides	HMDB05362
TG 16:0_34:3	Triacylglyceride 16:0_34:3	Triglycerides	HMDB05379
TG 16:0_34:4	Triacylglyceride 16:0_34:4	Triglycerides	HMDB0044074
TG 16:0_35:1	Triacylglyceride 16:0_35:1	Triglycerides	HMDB0043028
TG 16:0_35:2	Triacylglyceride 16:0_35:2	Triglycerides	HMDB0011700
TG 16:0_35:3	Triacylglyceride 16:0_35:3	Triglycerides	HMDB0011701
TG 16:0_36:2	Triacylglyceride 16:0_36:2	Triglycerides	HMDB05382
TG 16:0_36:3	Triacylglyceride 16:0_36:3	Triglycerides	HMDB05384
TG 16:0_36:4	Triacylglyceride 16:0_36:4	Triglycerides	HMDB05390

TG 16:0_36:5	Triacylglyceride 16:0_36:5	Triglycerides	HMDB05380
TG 16:0_36:6	Triacylglyceride 16:0_36:6	Triglycerides	HMDB0044298
TG 16:0_37:3	Triacylglyceride 16:0_37:3	Triglycerides	HMDB0043867
TG 16:0_38:1	Triacylglyceride 16:0_38:1	Triglycerides	HMDB05381
TG 16:0_38:2	Triacylglyceride 16:0_38:2	Triglycerides	HMDB05383
TG 16:0_38:3	Triacylglyceride 16:0_38:3	Triglycerides	HMDB05389
TG 16:0_38:4	Triacylglyceride 16:0_38:4	Triglycerides	HMDB05370
TG 16:0_38:5	Triacylglyceride 16:0_38:5	Triglycerides	HMDB05385
TG 16:0_38:6	Triacylglyceride 16:0_38:6	Triglycerides	HMDB10418
TG 16:0_38:7	Triacylglyceride 16:0_38:7	Triglycerides	HMDB0067816
TG 16:0_40:6	Triacylglyceride 16:0_40:6	Triglycerides	HMDB0044134
TG 16:0_40:7	Triacylglyceride 16:0_40:7	Triglycerides	HMDB0067085
TG 16:0_40:8	Triacylglyceride 16:0_40:8	Triglycerides	HMDB05392
TG 16:1_28:0	Triacylglyceride 16:1_28:0	Triglycerides	HMDB0042069
TG 16:1_30:1	Triacylglyceride 16:1_30:1	Triglycerides	HMDB0042309
TG 16:1_32:0	Triacylglyceride 16:1_32:0	Triglycerides	HMDB05359
TG 16:1_32:1	Triacylglyceride 16:1_32:1	Triglycerides	HMDB0044888
TG 16:1_32:2	Triacylglyceride 16:1_32:2	Triglycerides	HMDB0005432
TG 16:1_33:1	Triacylglyceride 16:1_33:1	Triglycerides	HMDB0043200
TG 16:1_34:1	Triacylglyceride 16:1_34:1	Triglycerides	HMDB0044889
TG 16:1_34:2	Triacylglyceride 16:1_34:2	Triglycerides	HMDB05433
TG 16:1_34:3	Triacylglyceride 16:1_34:3	Triglycerides	HMDB05435
TG 16:1_36:2	Triacylglyceride 16:1_36:2	Triglycerides	HMDB05438
TG 16:1_36:3	Triacylglyceride 16:1_36:3	Triglycerides	HMDB05440
TG 16:1_36:4	Triacylglyceride 16:1_36:4	Triglycerides	HMDB05446
TG 16:1_36:5	Triacylglyceride 16:1_36:5	Triglycerides	HMDB05436
TG 16:1_38:3	Triacylglyceride 16:1_38:3	Triglycerides	HMDB0046079
TG 16:1_38:4	Triacylglyceride 16:1_38:4	Triglycerides	HMDB0046105
TG 16:1_38:5	Triacylglyceride 16:1_38:5	Triglycerides	HMDB05441
TG 17:0_32:1	Triacylglyceride 17:0_32:1	Triglycerides	NA
TG 17:0_34:1	Triacylglyceride 17:0_34:1	Triglycerides	NA
TG 17:0_34:2	Triacylglyceride 17:0_34:2	Triglycerides	NA
TG 17:0_34:3	Triacylglyceride 17:0_34:3	Triglycerides	NA
TG 17:0_36:3	Triacylglyceride 17:0_36:3	Triglycerides	NA
TG 17:0_36:4	Triacylglyceride 17:0_36:4	Triglycerides	NA
TG 17:1_32:1	Triacylglyceride 17:1_32:1	Triglycerides	NA
TG 17:1_34:1	Triacylglyceride 17:1_34:1	Triglycerides	NA
TG 17:1_34:2	Triacylglyceride 17:1_34:2	Triglycerides	NA
TG 17:1_34:3	Triacylglyceride 17:1_34:3	Triglycerides	NA
TG 17:1_36:3	Triacylglyceride 17:1_36:3	Triglycerides	NA
TG 17:1_36:4	Triacylglyceride 17:1_36:4	Triglycerides	NA

TG 17:1_36:5	Triacylglyceride 17:1_36:5	Triglycerides	NA
TG 17:2_34:2	Triacylglyceride 17:2_34:2	Triglycerides	NA
TG 17:2_36:2	Triacylglyceride 17:2_36:2	Triglycerides	NA
TG 17:2_36:3	Triacylglyceride 17:2_36:3	Triglycerides	NA
TG 17:2_36:4	Triacylglyceride 17:2_36:4	Triglycerides	NA
TG 18:0_30:0	Triacylglyceride 18:0_30:0	Triglycerides	HMDB0108000
TG 18:0_30:1	Triacylglyceride 18:0_30:1	Triglycerides	HMDB0044726
TG 18:0_32:0	Triacylglyceride 18:0_32:0	Triglycerides	HMDB0068957
TG 18:0_32:1	Triacylglyceride 18:0_32:1	Triglycerides	HMDB0044753
TG 18:0_32:2	Triacylglyceride 18:0_32:2	Triglycerides	HMDB0044889
TG 18:0_34:3	Triacylglyceride 18:0_34:3	Triglycerides	HMDB05425
TG 18:0_36:2	Triacylglyceride 18:0_36:2	Triglycerides	HMDB05403
TG 18:0_36:3	Triacylglyceride 18:0_36:3	Triglycerides	HMDB05405
TG 18:0_36:4	Triacylglyceride 18:0_36:4	Triglycerides	HMDB05370
TG 18:0_36:5	Triacylglyceride 18:0_36:5	Triglycerides	HMDB05426
TG 18:0_38:6	Triacylglyceride 18:0_38:6	Triglycerides	HMDB05412
TG 18:0_38:7	Triacylglyceride 18:0_38:7	Triglycerides	HMDB0063278
TG 18:1_26:0	Triacylglyceride 18:1_26:0	Triglycerides	NA
TG 18:1_28:1	Triacylglyceride 18:1_28:1	Triglycerides	HMDB0042280
TG 18:1_30:0	Triacylglyceride 18:1_30:0	Triglycerides	HMDB0042130
TG 18:1_30:1	Triacylglyceride 18:1_30:1	Triglycerides	HMDB0047773
TG 18:1_30:2	Triacylglyceride 18:1_30:2	Triglycerides	HMDB0047910
TG 18:1_31:0	Triacylglyceride 18:1_31:0	Triglycerides	HMDB0043026
TG 18:1_32:0	Triacylglyceride 18:1_32:0	Triglycerides	HMDB05360
TG 18:1_32:1	Triacylglyceride 18:1_32:1	Triglycerides	HMDB0062639
TG 18:1_32:2	Triacylglyceride 18:1_32:2	Triglycerides	HMDB05433
TG 18:1_32:3	Triacylglyceride 18:1_32:3	Triglycerides	HMDB0042354
TG 18:1_33:0	Triacylglyceride 18:1_33:0	Triglycerides	HMDB0043055
TG 18:1_33:1	Triacylglyceride 18:1_33:1	Triglycerides	HMDB0043229
TG 18:1_33:2	Triacylglyceride 18:1_33:2	Triglycerides	HMDB0043235
TG 18:1_33:3	Triacylglyceride 18:1_33:3	Triglycerides	HMDB0043243
TG 18:1_34:1	Triacylglyceride 18:1_34:1	Triglycerides	HMDB05382
TG 18:1_34:2	Triacylglyceride 18:1_34:2	Triglycerides	HMDB05384
TG 18:1_34:3	Triacylglyceride 18:1_34:3	Triglycerides	HMDB05440
TG 18:1_34:4	Triacylglyceride 18:1_34:4	Triglycerides	HMDB0048626
TG 18:1_35:2	Triacylglyceride 18:1_35:2	Triglycerides	HMDB0043266
TG 18:1_35:3	Triacylglyceride 18:1_35:3	Triglycerides	HMDB0043267
TG 18:1_36:0	Triacylglyceride 18:1_36:0	Triglycerides	HMDB05395
TG 18:1_36:2	Triacylglyceride 18:1_36:2	Triglycerides	HMDB05439
TG 18:1_36:3	Triacylglyceride 18:1_36:3	Triglycerides	HMDB05455
TG 18:1_36:4	Triacylglyceride 18:1_36:4	Triglycerides	HMDB05385

TG 18:1_36:5	Triacylglyceride 18:1_36:5	Triglycerides	HMDB05441
TG 18:1_36:6	Triacylglyceride 18:1_36:6	Triglycerides	HMDB0042359
TG 18:1_38:5	Triacylglyceride 18:1_38:5	Triglycerides	HMDB05456
TG 18:1_38:6	Triacylglyceride 18:1_38:6	Triglycerides	HMDB05462
TG 18:1_38:7	Triacylglyceride 18:1_38:7	Triglycerides	HMDB0050036
TG 18:2_28:0	Triacylglyceride 18:2_28:0	Triglycerides	HMDB0042076
TG 18:2_30:0	Triacylglyceride 18:2_30:0	Triglycerides	HMDB0042136
TG 18:2_30:1	Triacylglyceride 18:2_30:1	Triglycerides	HMDB0042316
TG 18:2_31:0	Triacylglyceride 18:2_31:0	Triglycerides	HMDB0043032
TG 18:2_32:0	Triacylglyceride 18:2_32:0	Triglycerides	HMDB05362
TG 18:2_32:1	Triacylglyceride 18:2_32:1	Triglycerides	HMDB05379
TG 18:2_32:2	Triacylglyceride 18:2_32:2	Triglycerides	HMDB05435
TG 18:2_33:0	Triacylglyceride 18:2_33:0	Triglycerides	HMDB0011703
TG 18:2_33:1	Triacylglyceride 18:2_33:1	Triglycerides	HMDB0043235
TG 18:2_33:2	Triacylglyceride 18:2_33:2	Triglycerides	HMDB0011711
TG 18:2_34:1	Triacylglyceride 18:2_34:1	Triglycerides	HMDB0045870
TG 18:2_34:2	Triacylglyceride 18:2_34:2	Triglycerides	HMDB05390
TG 18:2_34:3	Triacylglyceride 18:2_34:3	Triglycerides	HMDB05446
TG 18:2_34:4	Triacylglyceride 18:2_34:4	Triglycerides	HMDB0048758
TG 18:2_35:1	Triacylglyceride 18:2_35:1	Triglycerides	HMDB0052765
TG 18:2_35:2	Triacylglyceride 18:2_35:2	Triglycerides	HMDB0052413
TG 18:2_35:3	Triacylglyceride 18:2_35:3	Triglycerides	HMDB0043406
TG 18:2_36:1	Triacylglyceride 18:2_36:1	Triglycerides	HMDB05389
TG 18:2_36:2	Triacylglyceride 18:2_36:2	Triglycerides	HMDB05455
TG 18:2_36:3	Triacylglyceride 18:2_36:3	Triglycerides	HMDB05461
TG 18:2_36:4	Triacylglyceride 18:2_36:4	Triglycerides	HMDB0005474
TG 18:2_36:5	Triacylglyceride 18:2_36:5	Triglycerides	HMDB05447
TG 18:2_38:4	Triacylglyceride 18:2_38:4	Triglycerides	HMDB05412
TG 18:2_38:5	Triacylglyceride 18:2_38:5	Triglycerides	HMDB0050760
TG 18:2_38:6	Triacylglyceride 18:2_38:6	Triglycerides	HMDB05475
TG 18:3_30:0	Triacylglyceride 18:3_30:0	Triglycerides	HMDB0043011
TG 18:3_32:0	Triacylglyceride 18:3_32:0	Triglycerides	HMDB10417
TG 18:3_32:1	Triacylglyceride 18:3_32:1	Triglycerides	HMDB0047809
TG 18:3_34:0	Triacylglyceride 18:3_34:0	Triglycerides	HMDB0043934
TG 18:3_34:1	Triacylglyceride 18:3_34:1	Triglycerides	HMDB0045896
TG 18:3_34:2	Triacylglyceride 18:3_34:2	Triglycerides	HMDB0048096
TG 18:3_34:3	Triacylglyceride 18:3_34:3	Triglycerides	HMDB0048758
TG 18:3_35:2	Triacylglyceride 18:3_35:2	Triglycerides	HMDB0043643
TG 18:3_36:1	Triacylglyceride 18:3_36:1	Triglycerides	HMDB0046079
TG 18:3_36:2	Triacylglyceride 18:3_36:2	Triglycerides	HMDB10460
TG 18:3_36:3	Triacylglyceride 18:3_36:3	Triglycerides	HMDB0044287

TG 18:3_36:4	Triacylglyceride 18:3_36:4	Triglycerides	HMDB10490
TG 18:3_38:5	Triacylglyceride 18:3_38:5	Triglycerides	HMDB0050768
TG 18:3_38:6	Triacylglyceride 18:3_38:6	Triglycerides	HMDB0053582
TG 20:0_32:3	Triacylglyceride 20:0_32:3	Triglycerides	HMDB0045870
TG 20:0_32:4	Triacylglyceride 20:0_32:4	Triglycerides	HMDB0045896
TG 20:0_34:1	Triacylglyceride 20:0_34:1	Triglycerides	HMDB0045584
TG 20:1_32:0	Triacylglyceride 20:1_32:0	Triglycerides	NA
TG 20:1_32:1	Triacylglyceride 20:1_32:1	Triglycerides	HMDB05378
TG 20:1_32:2	Triacylglyceride 20:1_32:2	Triglycerides	HMDB05434
TG 20:1_32:3	Triacylglyceride 20:1_32:3	Triglycerides	HMDB0048073
TG 20:1_34:1	Triacylglyceride 20:1_34:1	Triglycerides	HMDB05424
TG 20:1_34:2	Triacylglyceride 20:1_34:2	Triglycerides	HMDB05439
TG 20:1_34:3	Triacylglyceride 20:1_34:3	Triglycerides	HMDB05445
TG 20:2_32:0	Triacylglyceride 20:2_32:0	Triglycerides	HMDB0043900
TG 20:2_32:1	Triacylglyceride 20:2_32:1	Triglycerides	HMDB0044068
TG 20:2_34:1	Triacylglyceride 20:2_34:1	Triglycerides	HMDB0044096
TG 20:2_34:2	Triacylglyceride 20:2_34:2	Triglycerides	HMDB0042588
TG 20:2_34:3	Triacylglyceride 20:2_34:3	Triglycerides	HMDB0048125
TG 20:3_32:0	Triacylglyceride 20:3_32:0	Triglycerides	HMDB0042169
TG 20:3_32:1	Triacylglyceride 20:3_32:1	Triglycerides	HMDB0047798
TG 20:3_32:2	Triacylglyceride 20:3_32:2	Triglycerides	HMDB0048599
TG 20:3_34:0	Triacylglyceride 20:3_34:0	Triglycerides	HMDB0043929
TG 20:3_34:1	Triacylglyceride 20:3_34:1	Triglycerides	HMDB0044091
TG 20:3_34:2	Triacylglyceride 20:3_34:2	Triglycerides	HMDB0042589
TG 20:3_34:3	Triacylglyceride 20:3_34:3	Triglycerides	HMDB0048126
TG 20:3_36:3	Triacylglyceride 20:3_36:3	Triglycerides	HMDB0049390
TG 20:3_36:4	Triacylglyceride 20:3_36:4	Triglycerides	HMDB10492
TG 20:3_36:5	Triacylglyceride 20:3_36:5	Triglycerides	HMDB0044357
TG 20:4_30:0	Triacylglyceride 20:4_30:0	Triglycerides	HMDB0042146
TG 20:4_32:0	Triacylglyceride 20:4_32:0	Triglycerides	HMDB05363
TG 20:4_32:1	Triacylglyceride 20:4_32:1	Triglycerides	HMDB05380
TG 20:4_32:2	Triacylglyceride 20:4_32:2	Triglycerides	HMDB05436
TG 20:4_33:2	Triacylglyceride 20:4_33:2	Triglycerides	HMDB0043419
TG 20:4_34:0	Triacylglyceride 20:4_34:0	Triglycerides	HMDB05370
TG 20:4_34:1	Triacylglyceride 20:4_34:1	Triglycerides	HMDB05385
TG 20:4_34:2	Triacylglyceride 20:4_34:2	Triglycerides	HMDB05391
TG 20:4_34:3	Triacylglyceride 20:4_34:3	Triglycerides	HMDB05447
TG 20:4_36:2	Triacylglyceride 20:4_36:2	Triglycerides	HMDB05412
TG 20:4_36:3	Triacylglyceride 20:4_36:3	Triglycerides	HMDB05462
TG 20:4_36:4	Triacylglyceride 20:4_36:4	Triglycerides	HMDB05475
TG 20:4_36:5	Triacylglyceride 20:4_36:5	Triglycerides	HMDB05448

TG 20:5_34:1	Triacylglyceride 20:5_34:1	Triglycerides	HMDB0047835
TG 20:5_34:2	Triacylglyceride 20:5_34:2	Triglycerides	HMDB0048629
TG 20:5_36:2	Triacylglyceride 20:5_36:2	Triglycerides	HMDB10464
TG 20:5_36:3	Triacylglyceride 20:5_36:3	Triglycerides	HMDB0044357
TG 22:3_30:2	Triacylglyceride 22:3_30:2	Triglycerides	NA
TG 22:4_32:0	Triacylglyceride 22:4_32:0	Triglycerides	HMDB0042172
TG 22:4_34:2	Triacylglyceride 22:4_34:2	Triglycerides	HMDB0042592
TG 22:5_32:0	Triacylglyceride 22:5_32:0	Triglycerides	HMDB0043910
TG 22:5_32:1	Triacylglyceride 22:5_32:1	Triglycerides	HMDB0047813
TG 22:5_34:1	Triacylglyceride 22:5_34:1	Triglycerides	HMDB0047836
TG 22:5_34:2	Triacylglyceride 22:5_34:2	Triglycerides	HMDB0047997
TG 22:5_34:3	Triacylglyceride 22:5_34:3	Triglycerides	HMDB0044498
TG 22:6_32:0	Triacylglyceride 22:6_32:0	Triglycerides	HMDB10418
TG 22:6_32:1	Triacylglyceride 22:6_32:1	Triglycerides	HMDB0042359
TG 22:6_34:1	Triacylglyceride 22:6_34:1	Triglycerides	HMDB0044107
TG 22:6_34:2	Triacylglyceride 22:6_34:2	Triglycerides	HMDB0048631
TG 22:6_34:3	Triacylglyceride 22:6_34:3	Triglycerides	HMDB0048136
Hex3Cer d18:1/16:0	Trihexosylceramide d18:1/16:0	Trihexosylceramides	HMDB0004879
Hex3Cer d18:1/18:0	Trihexosylceramide d18:1/18:0	Trihexosylceramides	HMDB0004880
Hex3Cer d18:1/22:0	Trihexosylceramide d18:1/22:0	Trihexosylceramides	HMDB0004882
Hex3Cer d18:1/24:1	Trihexosylceramide d18:1/24:1	Trihexosylceramides	HMDB0004883
Biotin (B7)	Biotin (B7)	Vitamins and cofactors	HMDB0000030
Choline	Choline	Vitamins and cofactors	HMDB0000097
Nicotinamide (B3)	Nicotinamide (B3)	Vitamins and cofactors	HMDB0001406
Pantothenic acid (B5)	Pantothenic acid (B5)	Vitamins and cofactors	HMDB0250782

SUPPLEMENTAL DATA 3

Short name	Formula
TMAO Synthesis	TMAO / (Betaine + C0 + Choline)
TMAO Synthesis (direct)	TMAO / Choline
AGAT Deficiency	Arg / Guanidinoacetic acid
Asn Synthesis	Asn / Asp
Brain Trp Availability	Trp / (Leu + Ile + Val + Tyr + Phe + Met)
Cys Synthesis	Cys / (Ser + Met)
DLD (NBS)	Pro / Phe
Fischer Ratio	(Ile + Leu + Val) / (Phe + Trp + Tyr)
GABR	Arg / (Orn + Cit)
Glutaminase Activity	Glu / Gln
Glutaminolysis Rate	(Ala + Asp + Glu + Lac + Suc) / Gln
GSH Constituents	Glu + Gly + Cys
Gly Synthesis	Gly / Ser
MTHFR Deficiency (NBS)	Met / Phe
PKU (NBS)	Tyr / Phe
Glx to N-Ac-Asp Ratio	(Gln + Glu) / N-Ac-Asp
Glu to a-Ketoglutarate Ratio	Glu / a-Ketoglutaric acid
Gly to Ala Ratio	Gly / Ala
Ratio of His to HCys+Phe+Sar	His / (HCys + Phe + Sarcosine)
Ratio of Non-Essential to Essential AAs	(Ala + Arg + Asn + Asp + Cys + Gln + Glu + Gly + Pro + Ser + Tyr) / (His + Ile + Leu + Lys + Met + Phe + Thr + Trp + Val)
Ratio of Pro to Cit	Pro / Cit
Ratio of SG to Glucose	(Gly + Ser) / Glucose
Ratio of SGA to Glucose	(Gly + Ser + Ala) / Glucose
Sum of AAs	Ala + Arg + Asn + Asp + Cys + Gln + Glu + Gly + His + Ile + Leu + Lys + Met + Phe + Pro + Ser + Thr + Trp + Tyr + Val
Sum of Aromatic AAs	Phe + Trp + Tyr
Sum of BCAAs	Ile + Leu + Val
Sum of Essential AAs	His + Ile + Leu + Lys + Met + Phe + Thr + Trp + Val
Sum of Non-Essential AAs	Ala + Arg + Asn + Asp + Cys + Gln + Glu + Gly + Pro + Ser + Tyr
Sum of Solely Glucogenic AAs	Ala + Arg + Asn + Asp + Cys + Gln + Glu + Gly + His + Met + Pro + Ser + Thr + Val
Sum of Solely Ketogenic AAs	Leu + Lys
Sum of Sulfur-Containing AAs	Met + Cys
Valinemia (NBS)	Val / Phe
1-Met-His Synthesis	1-Met-His / His
AABA Synthesis	AABA / Thr
ACY1 Deficiency	(N-Ac-Met + N-Ac-Glu + N-Ac-Gly + N-Ac-Ala + N-Ac-Leu + N-Ac-Ile + N-Ac-Val) / (Met + Glu + Gly + Ala + Leu + Ile + Val)
ACY2 Deficiency	N-Ac-Asp / Asp
Asp Methylation	N-Met-Asp / Asp

Asymmetrical Arg Methylation	ADMA / Arg
BABA Synthesis	BABA / Glu
BAIBA Synthesis	BAIBA / Val
Betaine Synthesis	Betaine / Choline
BHMT Activity	DMG / Betaine
CPS Deficiency (NBS)	Cit / Phe
Carnosine Synthesis	Carnosine / His
Cit Synthesis	Cit / Orn
Citrullinemia	Cit / (Orn + Arg)
Cystine Synthesis	Cystine / Cys
Glu Acetylation	N-Ac-Glu / Glu
GAMT Deficiency	Guanidinoacetic acid / Creatine
HArg Synthesis	HArg / (Arg + Lys)
HCys Synthesis	HCys / Met
Imidazolepropionic Acid Synthesis	Imidazolepropionic acid / His
IDO Activity	Kynurenine / Trp
BCAT Ile Catabolism	3-Met-2-oxovaleric acid / Ile
Kynureninase A Activity	Anthranilic acid / Kynurenine
BCAT Leu Catabolism	4-Met-2-oxovaleric acid / Leu
Lys Carbamylation	HCit / Lys
Met Oxidation	Met-SO / Met
Muscle Protein Degradation	1-Met-His / Creatinine
NOS activity	Cit / Arg
Orn Synthesis	Orn / Arg
OTC Deficiency (NBS)	Orn / Cit
Pro Hydroxylation	(c4-OH-Pro + t4-OH-Pro) / Pro
3-Met-His to 1-Met-His Ratio	3-Met-His / 1-Met-His
Creatine to Creatinine Ratio	Creatine / Creatinine
Creatinine to Bilirubin Ratio	Creatinine / Bilirubin
GAA to HArg Ratio	Guanidinoacetic acid / HArg
Ratio of HArg to ADMA	HArg / ADMA
Ratio of HArg to SDMA	HArg / SDMA
HSer to Creatinine Ratio	HSer / Creatinine
Kynurenine to Serotonin Ratio	Kynurenine / Serotonin
Sarcosine Synthesis from Choline	Sarcosine / Choline
Sarcosine Synthesis from Gly	Sarcosine / Gly
Sum of Aminobutyric Acids	AABA + BABA + GABA
Sum of Asym. and Sym. Arg Methylation	(ADMA + SDMA) / Arg
Sum of BC-a-ketoacids	3-Met-2-oxovaleric acid + a-Ketoisovaleric acid + 4-Met-2-oxovaleric acid
Sum of Dimethylated Arg	ADMA + SDMA
Symmetrical Arg Methylation	SDMA / Arg

Taurine Synthesis	Taurine / Cys
BCAT Val Catabolism	a-Ketoisovaleric acid / Val
12-KetoDCA to DCA Ratio	12-KetoDCA / DCA
7a-Dehydroxylation of CA	DCA / CA
Gly Conjugation of CDCA	GCDCA / CDCA
Gly Conjugation of CA	GCA / CA
Gly Conjugation of DCA	GDCA / DCA
Gly Conjugation of Primary BAs	(GCA + GCDCA) / (CA + CDCA)
Gly Conjugation of UDCA	GUDCA / UDCA
GDCA Synthesis from CA	GDCA / CA
GLCA Synthesis from CDCA	GLCA / CDCA
Ratio of CDCA to CA	CDCA / CA
Primary BA Conjugation	(GCA + GCDCA + TCA + TCDCA) / (CA + CDCA)
IsoUDCA to UDCA Ratio	IsoUDCA / UDCA
Sum of 12a-OH BAs	3-EpiDCA + 7-KetoDCA + CA + DCA + GCA + GDCA + NorDCA + TCA + TDCA + THDCA
Sum of Conjugated Primary BAs	GCA + GCDCA + TCA + TCDCA
Sum of Gly-Conjugated BAs	GCA + GCDCA + GDCA + GLCA + GUDCA
Sum of Primary BAs	CA + CDCA + GCA + GCDCA + TCA + TCDCA
Sum of Unconjugated Primary BAs	CA + CDCA
Taurine Conjugation of CDCA	TCDCA / CDCA
Taurine Conjugation of CA	TCA / CA
Taurine Conjugation of DCA	TDCA / DCA
Taurine Conjugation of Primary BAs	(TCA + TCDCA) / (CA + CDCA)
Taurine Conjugation of UDCA	TUDCA / UDCA
TDCA Synthesis from CA	TDCA / CA
TLCA Synthesis from CDCA	TLCA / CDCA
UDCA Synthesis from CDCA	UDCA / CDCA
b-Ala Synthesis	beta-Ala / Carnosine
GABA Synthesis	GABA / Glu
Histamine Synthesis	Histamine / His
Putrescine Synthesis	Putrescine / Orn
Urea to Creatinine Ratio	Urea / Creatinine
Serotonin Synthesis	Serotonin / Trp
Spermidine Synthesis	Spermidine / Putrescine
Benzoic Acid Conjugation	HipAcid / Benzoic acid
Cholesterol Synthesis	Mevalonic acid / Creatinine
HipAcid Synthesis	HipAcid / Gly
HMGCR Activity	Mevalonic acid / 3-HMGA
Ketone Body Synthesis	3-OH-Butyric acid / FA 4:0
LDH Activity	Lac / Glucose
MRC Disorders	Lac / Pyruvic acid

Pyruvate Carboxylase Deficiency	Pyruvic acid / Oxaloacetic acid
Glycolic Acid to Oxalic Acid Ratio	Glycolic acid / Oxalic acid
Lac to FA 2:0 Ratio	Lac / FA 2:0
Pyruvate to Glucose Ratio	Pyruvic acid / Glucose
Sum of Carboxylic Acids	2-OH-Butyric acid + 3-OH-Butyric acid + Glycolic acid + Glyoxylic acid + HipAcid + Lac + Mevalonic acid + Pyruvic acid
p-Cresol-SO4 Synthesis	p-Cresol-SO4 / Tyr
p-Cresol-SO4 to p-Cresol Glucuronide	p-Cresol-SO4 / p-Cresol glucuronide
Total p-Cresol Derivatives	p-Cresol-SO4 + p-Cresol glucuronide
2-OH-Glutarate Synthesis	2-OH-Glutaric acid / a-Ketoglutaric acid
3-MAG-uria Type 1	Z-3-Met-glutaconic acid / E-3-Met-glutaconic acid
ACOD1 Activity	Itaconic acid / AconAcid
CMAMMA	Malonic acid / Methylmalonic acid
Ethylmalonic Aciduria	Ethylmalonic acid / Suc
Fumarase Activity	Malic acid / Fumaric acid
GCDH Deficiency	3-OH-Glutaric acid / Glutaric acid
MDH1 Activity	Malic acid / Oxaloacetic acid
MDH2 Activity	Oxaloacetic acid / Malic acid
a-Ketoglutarate to Citrate Ratio	a-Ketoglutaric acid / Citric acid
Suc to a-Ketoglutarate Ratio	Suc / a-Ketoglutaric acid
Secondary 3MG Aciduria	3-Met-glutaric acid / (Z-3-Met-glutaconic acid + E-3-Met-glutaconic acid)
SDH Activity	Suc / Fumaric acid
FA 2:0 to Glucose Ratio	FA 2:0 / Glucose
Acetic Acid to Butyric Acid Ratio	FA 2:0 / FA 4:0
Acetic Acid to Isobutyric Acid Ratio	FA 2:0 / FA 3:0-2M
Butyric Acid to Isobutyric Acid Ratio	FA 4:0 / FA 3:0-2M
FA 8:0 to FA 10:0 Ratio	FA 8:0 / FA 10:0
FA 16:0 to FA 18:1 Ratio	FA 16:0 / FA 18:1
FA16:0 to FA 16:1 Ratio	FA 16:0 / FA 16:1
SCD-1 index	FA 18:1 / FA 18:0
Sum BCFA	FA 3:0-2M + FA 4:0-2M + FA 4:0-3M + FA 5:0-3M + FA 5:0-4M
Sum MCFA	FA 7:0 + FA 8:0 + FA 9:0 + FA 10:0 + FA 11:0 + FA 12:0
Sum of MUFAs	FA 14:1n-5 + FA 16:1 + FA 18:1 + FA 20:1n-9 + FA 24:1n-9
MCFA PPAR Modulators	FA 8:0 + FA 9:0 + FA 10:0
Sum of FA 12:0 - FA 16:0	FA 12:0 + FA 14:0 + FA 15:0 + FA 16:0
Cortisone Synthesis	Cortisone / Cortisol
Sum of Steroid Hormones	Cortisol + Cortisone + DHEAS
Indole Pathway Activity	3-IPA / Trp
5-HIAA to Creatinine Ratio	5-HIAA / Creatinine
5-HIAA / Quinolinic Acid Ratio	5-HIAA / Quinolinic acid
Serotonin Catabolism	5-HIAA / Serotonin

Serotonin Pathway Activity	5-HIAA / Trp
Sum of AAA-derived Lactic Acids	Indole-Lac + Phenyl-Lac + OH-Phenyl-Lac
NAT10 Activity	N-Ac-Cytidine / Cytidine
AHCys to Leu Ratio	AHCys / Leu
Uric Acid to Creatinine Ratio	Uric acid / Creatinine
AHCY Deficiency	AHCys / (Adenosine + HCys)
Xanthine Synthesis	Xanthine / Hypoxanthine
Cyclic Nucleotides Ratio	cAMP / cGMP
BVRA Activity	Bilirubin / Biliverdin
Phenylpyruvate Synthesis	Phenylpyruvic acid / Phe
TAT Activity	4-OH-Phenylpyruvic acid / Tyr
Homovanillate to Vanillylmandelate Ratio	Homovanillic acid / Vanillylmandelic acid
Phenylacetate to PAGln Ratio	Phenylacetic acid / Phenylacetylglutamine
Phenylpyruvate to Citrate Ratio	Phenylpyruvic acid / Citric acid
N-Ac-Putrescine to N1- and N8-Ac-Spermidine Ratio	N-Ac-Putrescine / (N1-Ac-Spermidine + N8-Ac-Spermidine)
Spermidine Acetylation	(N1-Ac-Spermidine + N8-Ac-Spermidine + N1,N8-Di-Ac-Spermidine) / Spermidine
AMSDH Saturation	Picolinic acid / Quinolinic acid
KYNA DH	Quinaldic acid / Kynurenic acid
KAT A Activity	Kynurenic acid / Kynurenine
Ratio Quinolinic Acid to KYNA	Quinolinic acid / Kynurenic acid
Kynurenic Acid to Xanthurenic Acid Ratio	Kynurenic acid / Xanthurenic acid
Picolinic Acid to Trp Ratio	Picolinic acid / Trp
Fructose to Glucose Ratio	Fructose / Glucose
Aconitase Activity	Isocitric acid / Citric acid
CS Activity	Citric acid / Oxaloacetic acid
Aconitate to Citrate Ratio	AconAcid / Citric acid
Choline to Creatinine Ratio	Choline / Creatinine
2MBG (NBS)	C5 / C3
b-Oxidation	(C2 + C3) / C0
CACT Deficiency (NBS)	(C16 + C18) / C0
CPT-1 Deficiency (NBS)	C0 / (C16 + C18)
CPT-2 Deficiency (NBS)	(C16 + C18:1) / C2
Carnitine Uptake Defect (NBS)	(C0 + C2 + C3 + C16 + C18 + C18:1) / Cit
EMA (NBS)	C4 / C8
IBD Deficiency (NBS)	C4 / C2
IVA (NBS)	C5 / C2
MA (NBS)	C3 / C2
MCAD Deficiency (NBS)	C8 / C2
MCKAT Deficiency (NBS)	C8 / C10
MMA (NBS)	C3 / C0
MC Deficiency (NBS)	C16 / C3

PA (NBS)	C3 / C16
Ratio of Acetylcarnitine to Carnitine	C2 / C0
Carnitine to Creatinine Ratio	C0 / Creatinine
SBCAD Deficiency (NBS)	C5 / C0
SCAD Deficiency (NBS)	C4 / C3
VLCAD Deficiency (NBS)	C14:1 / C16
Sum of VLCFA-Cer	Cer d16:1/22:0 + Cer d16:1/23:0 + Cer d16:1/24:0 + Cer d18:1/22:0 + Cer d18:1/23:0 + Cer d18:1/24:0 + Cer d18:1/24:1 + Cer d18:1/25:0 + Cer d18:1/26:0 + Cer d18:1/26:1 + Cer d18:2/22:0 + Cer d18:2/23:0 + Cer d18:2/24:0 + Cer d18:2/24:1
Sum of PUFA-CEs	CE 18:2 + CE 18:3 + CE 20:3 + CE 20:4 + CE 20:5 + CE 22:2 + CE 22:5 + CE 22:6
Protection against MDD	DG 18:1_18:2 / DG 18:2_20:4
Ratio of MUFA-LPCs to SFA-LPCs	(LPC 26:1 + LPC 18:1 + LPC 16:1) / (LPC 26:0 + LPC 24:0 + LPC 18:0 + LPC 17:0 + LPC 16:0)
Ratio of PUFA-LPCs to MUFA-LPCs	(LPC 20:4 + LPC 20:3 + LPC 18:2) / (LPC 26:1 + LPC 18:1 + LPC 16:1)
Ratio of PUFA-LPCs to SFA-LPCs	(LPC 20:4 + LPC 20:3 + LPC 18:2) / (LPC 26:0 + LPC 24:0 + LPC 18:0 + LPC 17:0 + LPC 16:0)
Ratio of UFA-LPCs to SFA-LPCs	(LPC 26:1 + LPC 20:4 + LPC 20:3 + LPC 18:2 + LPC 18:1 + LPC 16:1) / (LPC 26:0 + LPC 24:0 + LPC 18:0 + LPC 17:0 + LPC 16:0)
Sum of LCFA-LPCs	LPC 14:0 + LPC 16:0 + LPC 16:1 + LPC 17:0 + LPC 18:0 + LPC 18:1 + LPC 18:2 + LPC 20:3 + LPC 20:4
Sum of LPCs	LPC 14:0 + LPC 16:0 + LPC 16:1 + LPC 17:0 + LPC 18:0 + LPC 18:1 + LPC 18:2 + LPC 20:3 + LPC 20:4 + LPC 24:0 + LPC 26:0 + LPC 26:1
Sum of MUFA-LPCs	LPC 16:1 + LPC 18:1 + LPC 26:1
Sum of PUFA-LPCs	LPC 18:2 + LPC 20:3 + LPC 20:4
Sum of SFA-LPCs	LPC 14:0 + LPC 16:0 + LPC 17:0 + LPC 18:0 + LPC 24:0 + LPC 26:0
Sum of UFA-LPCs	LPC 16:1 + LPC 18:1 + LPC 18:2 + LPC 20:3 + LPC 20:4 + LPC 26:1
Sum of VLCFA-LPCs	LPC 24:0 + LPC 26:0 + LPC 26:1
Sum of MUFA-LPGs	LPG 14:1 + LPG 16:1 + LPG 17:1 + LPG 18:1 + LPG 20:1
Sum of UFA-LPGs	LPG 14:1 + LPG 16:1 + LPG 17:1 + LPG 18:1 + LPG 18:2 + LPG 20:1
PLA activity (2)	(LPI 22:1 + LPI 22:0 + LPI 20:4 + LPI 20:1 + LPI 19:0 + LPI 18:3 + LPI 18:2 + LPI 18:1 + LPI 18:0 + LPI 17:1 + LPI 17:0 + LPI 16:1 + LPI 16:0 + LPI 14:1 + LPI 14:0) / (PI 18:2_22:6 + PI 18:2_22:1 + PI 18:2_22:0 + PI 18:2_20:5 + PI 18:2_20:4 + PI 18:2_20:1 + PI 18:2_20:0 + PI 18:2_18:3 + PI 18:1_22:6 + PI 18:1_22:5 + PI 18:1_22:4 + PI 18:1_22:3 + PI 18:1_22:2 + PI 18:1_22:1 + PI 18:1_22:0 + PI 18:1_20:5 + PI 18:1_20:4 + PI 18:1_20:3 + PI 18:1_20:2 + PI 18:1_20:1 + PI 18:1_20:0 + PI 18:1_18:3 + PI 18:1_18:2 + PI 18:1_18:1 + PI 18:0_22:0 + PI 18:0_20:4 + PI 18:0_20:3 + PI 18:0_20:0 + PI 18:0_18:3 + PI 18:0_18:2 + PI 18:0_18:1 + PI 18:0_18:0 + PI 17:1_18:2 + PI 17:1_18:1 + PI 17:0_18:1 + PI 16:1_18:2 + PI 16:1_18:1 + PI 16:1_18:0 + PI 16:0_22:1 + PI 16:0_20:4 + PI 16:0_20:3 + PI 16:0_20:0 + PI 16:0_18:3 + PI 16:0_18:2 + PI 16:0_18:1 + PI 16:0_17:2 + PI 16:0_17:1 + PI 16:0_17:0 + PI 16:0_16:0 + PI 15:1_16:0 + PI 15:0_16:0 + PI 14:0_18:2 + PI 14:0_18:1)

Ratio of MUFA-LPIs to SFA-LPIs	(LPI 14:1 + LPI 16:1 + LPI 17:1 + LPI 18:1 + LPI 20:1 + LPI 22:1) / (LPI 14:0 + LPI 16:0 + LPI 17:0 + LPI 18:0 + LPI 19:0 + LPI 22:0)
Ratio of PUFA-LPIs to MUFA-LPIs	(LPI 18:2 + LPI 18:3 + LPI 20:4) / (LPI 14:1 + LPI 16:1 + LPI 17:1 + LPI 18:1 + LPI 20:1 + LPI 22:1)
Ratio of PUFA-LPIs to SFA-LPIs	(LPI 18:2 + LPI 18:3 + LPI 20:4) / (LPI 14:0 + LPI 16:0 + LPI 17:0 + LPI 18:0 + LPI 19:0 + LPI 22:0)
Ratio of UFA-LPIs to SFA-LPIs	(LPI 14:1 + LPI 16:1 + LPI 17:1 + LPI 18:1 + LPI 18:2 + LPI 18:3 + LPI 20:1 + LPI 20:4 + LPI 22:1) / (LPI 14:0 + LPI 16:0 + LPI 17:0 + LPI 18:0 + LPI 19:0 + LPI 22:0)
Sum of LCFA-LPIs	LPI 14:0 + LPI 14:1 + LPI 16:0 + LPI 16:1 + LPI 17:0 + LPI 17:1 + LPI 18:0 + LPI 18:1 + LPI 18:2 + LPI 18:3 + LPI 19:0 + LPI 20:1 + LPI 20:4
Sum of LPIs	LPI 14:0 + LPI 14:1 + LPI 16:0 + LPI 16:1 + LPI 17:0 + LPI 17:1 + LPI 18:0 + LPI 18:1 + LPI 18:2 + LPI 18:3 + LPI 19:0 + LPI 20:1 + LPI 20:4 + LPI 22:0 + LPI 22:1
Sum of MUFA-LPIs	LPI 14:1 + LPI 16:1 + LPI 17:1 + LPI 18:1 + LPI 20:1 + LPI 22:1
Sum of (L)PIs	LPI 14:0 + LPI 14:1 + LPI 16:0 + LPI 16:1 + LPI 17:0 + LPI 17:1 + LPI 18:0 + LPI 18:1 + LPI 18:2 + LPI 18:3 + LPI 19:0 + LPI 20:1 + LPI 20:4 + LPI 22:0 + LPI 22:1 + PI 14:0_18:1 + PI 14:0_18:2 + PI 15:0_16:0 + PI 15:1_16:0 + PI 16:0_16:0 + PI 16:0_17:0 + PI 16:0_17:1 + PI 16:0_17:2 + PI 16:0_18:1 + PI 16:0_18:2 + PI 16:0_18:3 + PI 16:0_20:0 + PI 16:0_20:3 + PI 16:0_20:4 + PI 16:0_22:1 + PI 16:1_18:0 + PI 16:1_18:1 + PI 16:1_18:2 + PI 17:0_18:1 + PI 17:1_18:1 + PI 17:1_18:2 + PI 18:0_18:0 + PI 18:0_18:1 + PI 18:0_18:2 + PI 18:0_18:3 + PI 18:0_20:0 + PI 18:0_20:3 + PI 18:0_20:4 + PI 18:0_22:0 + PI 18:1_18:1 + PI 18:1_18:2 + PI 18:1_18:3 + PI 18:1_20:0 + PI 18:1_20:1 + PI 18:1_20:2 + PI 18:1_20:3 + PI 18:1_20:4 + PI 18:1_20:5 + PI 18:1_22:0 + PI 18:1_22:1 + PI 18:1_22:2 + PI 18:1_22:3 + PI 18:1_22:4 + PI 18:1_22:5 + PI 18:1_22:6 + PI 18:2_18:3 + PI 18:2_20:0 + PI 18:2_20:1 + PI 18:2_20:4 + PI 18:2_20:5 + PI 18:2_22:0 + PI 18:2_22:1 + PI 18:2_22:6
Sum of PUFA-LPIs	LPI 18:2 + LPI 18:3 + LPI 20:4
Sum of SFA-LPIs	LPI 14:0 + LPI 16:0 + LPI 17:0 + LPI 18:0 + LPI 19:0 + LPI 22:0
Sum of UFA-LPIs	LPI 14:1 + LPI 16:1 + LPI 17:1 + LPI 18:1 + LPI 18:2 + LPI 18:3 + LPI 20:1 + LPI 20:4 + LPI 22:1
Sum of VLCFA-LPIs	LPI 22:0 + LPI 22:1
Sum of MUFA-PAs	PA 14:0_14:1 + PA 16:0_18:1 + PA 16:1_18:1 + PA 16:1_22:0 + PA 17:0_18:1 + PA 17:1_18:1 + PA 18:0_18:1 + PA 18:1_18:1 + PA 18:1_20:0 + PA 18:1_20:1 + PA 18:1_22:0 + PA 18:1_22:1
Ratio of PUFA-PCs O to MUFA-PCs O	(PC O-34:3 + PC O-36:3 + PC O-36:4 + PC O-36:5 + PC O-38:3 + PC O-38:4 + PC O-38:5 + PC O-38:6 + PC O-40:3 + PC O-40:4 + PC O-40:5 + PC O-40:6 + PC O-42:3 + PC O-42:4 + PC O-42:5 + PC O-44:3 + PC O-44:4 + PC O-44:5 + PC O-44:6) / (PC O-28:1 + PC O-30:1 + PC O-32:1 + PC O-34:1 + PC O-36:1 + PC O-38:1 + PC O-40:1 + PC O-42:1)
Sum of MUFA-PCs O	PC O-28:1 + PC O-30:1 + PC O-32:1 + PC O-34:1 + PC O-36:1 + PC O-38:1 + PC O-40:1 + PC O-42:1
Sum of PUFA-PCs O	PC O-34:3 + PC O-36:3 + PC O-36:4 + PC O-36:5 + PC O-38:3 + PC O-38:4 + PC O-38:5 + PC O-38:6 + PC O-40:3 + PC O-40:4 + PC O-40:5 + PC O-40:6 + PC O-42:3 + PC O-42:4 + PC O-42:5 + PC O-44:3 + PC O-44:4 + PC O-44:5 + PC O-44:6
Sum of PUFA-PCs	PC 32:3 + PC 34:3 + PC 34:4 + PC 36:3 + PC 36:4 + PC 36:5 + PC 36:6 + PC 38:3 + PC 38:4 + PC 38:5 + PC 38:6 + PC 40:3 + PC 40:4 + PC 40:5 + PC 40:6 + PC 42:4 + PC 42:5 + PC 42:6

Sum of PUFA-PC (O)s	PC 32:3 + PC 34:3 + PC 34:4 + PC 36:3 + PC 36:4 + PC 36:5 + PC 36:6 + PC 38:3 + PC 38:4 + PC 38:5 + PC 38:6 + PC 40:3 + PC 40:4 + PC 40:5 + PC 40:6 + PC 42:4 + PC 42:5 + PC 42:6 + PC O-34:3 + PC O-36:3 + PC O-36:4 + PC O-36:5 + PC O-38:3 + PC O-38:4 + PC O-38:5 + PC O-38:6 + PC O-40:3 + PC O-40:4 + PC O-40:5 + PC O-40:6 + PC O-42:3 + PC O-42:4 + PC O-42:5 + PC O-44:3 + PC O-44:4 + PC O-44:5 + PC O-44:6
Sum of UFA-PCs O	PC O-28:1 + PC O-30:1 + PC O-30:2 + PC O-32:1 + PC O-32:2 + PC O-34:1 + PC O-34:2 + PC O-34:3 + PC O-36:1 + PC O-36:2 + PC O-36:3 + PC O-36:4 + PC O-36:5 + PC O-38:1 + PC O-38:2 + PC O-38:3 + PC O-38:4 + PC O-38:5 + PC O-38:6 + PC O-40:1 + PC O-40:2 + PC O-40:3 + PC O-40:4 + PC O-40:5 + PC O-40:6 + PC O-42:1 + PC O-42:2 + PC O-42:3 + PC O-42:4 + PC O-42:5 + PC O-44:3 + PC O-44:4 + PC O-44:5 + PC O-44:6
Sum of PUFA-PEs	PE 34:3 + PE 34:4 + PE 35:3 + PE 36:3 + PE 36:4 + PE 36:5 + PE 36:6 + PE 38:3 + PE 38:4 + PE 38:5 + PE 38:6 + PE 38:7 + PE 40:3 + PE 40:4 + PE 40:5 + PE 40:6 + PE 40:7 + PE 40:8 + PE 42:7 + PE 42:8 + PE 44:6 + PE 44:7 + PE 44:11 + PE 44:12
Ratio of MUFA-PGs to SFA-PGs	(PG 15:0_18:1 + PG 16:0_16:1 + PG 16:0_18:1 + PG 16:0_19:1 + PG 16:0_22:1 + PG 16:1_16:1 + PG 16:1_18:0 + PG 16:1_18:1 + PG 16:1_22:1 + PG 17:0_18:1 + PG 17:1_18:1 + PG 18:0_18:1 + PG 18:0_22:1 + PG 18:1_18:1 + PG 18:1_20:0 + PG 18:1_20:1 + PG 18:1_22:0 + PG 18:1_22:1) / (PG 14:0_16:0 + PG 16:0_16:0)
Sum of MUFA-PGs	PG 16:1_16:1 + PG 18:1_20:1 + PG 16:0_19:1 + PG 16:0_22:1 + PG 18:0_18:1 + PG 18:0_22:1 + PG 15:0_18:1 + PG 16:1_18:1 + PG 16:0_16:1 + PG 16:1_22:1 + PG 17:0_18:1 + PG 17:1_18:1 + PG 18:1_18:1 + PG 16:0_18:1 + PG 18:1_22:1 + PG 16:1_18:0 + PG 18:1_20:0 + PG 18:1_22:0
Sum of SFA-PGs	PG 16:0_16:0 + PG 14:0_16:0
Ratio of MUFA-PIs to SFA-PIs	(PI 14:0_18:1 + PI 15:1_16:0 + PI 16:0_17:1 + PI 16:0_18:1 + PI 16:0_22:1 + PI 16:1_18:0 + PI 17:0_18:1 + PI 18:0_18:1 + PI 18:1_20:0 + PI 18:1_22:0 + PI 18:1_22:1 + PI 18:1_20:1 + PI 17:1_18:1 + PI 18:1_18:1 + PI 16:1_18:1) / (PI 15:0_16:0 + PI 16:0_16:0 + PI 16:0_17:0 + PI 16:0_20:0 + PI 18:0_18:0 + PI 18:0_20:0 + PI 18:0_22:0)
Ratio of PUFA-PIs to MUFA-PIs	(PI 14:0_18:2 + PI 16:0_17:2 + PI 16:0_18:2 + PI 16:0_18:3 + PI 16:0_20:3 + PI 16:0_20:4 + PI 16:1_18:2 + PI 17:1_18:2 + PI 18:0_18:2 + PI 18:0_18:3 + PI 18:0_20:3 + PI 18:0_20:4 + PI 18:1_18:2 + PI 18:1_18:3 + PI 18:1_20:2 + PI 18:1_20:3 + PI 18:1_20:4 + PI 18:1_20:5 + PI 18:1_22:2 + PI 18:1_22:3 + PI 18:1_22:4 + PI 18:1_22:5 + PI 18:1_22:6 + PI 18:2_18:3 + PI 18:2_20:0 + PI 18:2_20:1 + PI 18:2_20:4 + PI 18:2_20:5 + PI 18:2_22:0 + PI 18:2_22:1 + PI 18:2_22:6) / (PI 14:0_18:1 + PI 15:1_16:0 + PI 16:0_17:1 + PI 16:0_18:1 + PI 16:0_22:1 + PI 16:1_18:0 + PI 17:0_18:1 + PI 18:0_18:1 + PI 18:1_20:0 + PI 18:1_22:0 + PI 18:1_22:1 + PI 18:1_20:1 + PI 18:1_18:1 + PI 17:1_18:1 + PI 16:1_18:1)
Ratio of PUFA-PIs to SFA-PIs	(PI 14:0_18:2 + PI 16:0_17:2 + PI 16:0_18:2 + PI 16:0_18:3 + PI 16:0_20:3 + PI 16:0_20:4 + PI 16:1_18:2 + PI 17:1_18:2 + PI 18:0_18:2 + PI 18:0_18:3 + PI 18:0_20:3 + PI 18:0_20:4 + PI 18:1_18:2 + PI 18:1_18:3 + PI 18:1_20:2 + PI 18:1_20:3 + PI 18:1_20:4 + PI 18:1_20:5 + PI 18:1_22:2 + PI 18:1_22:3 + PI 18:1_22:4 + PI 18:1_22:5 + PI 18:1_22:6 + PI 18:2_18:3 + PI 18:2_20:0 + PI 18:2_20:1 + PI 18:2_20:4 + PI 18:2_20:5 + PI 18:2_22:0 + PI 18:2_22:1 + PI 18:2_22:6) / (PI 15:0_16:0 + PI 16:0_16:0 + PI 16:0_17:0 + PI 16:0_20:0 + PI 18:0_18:0 + PI 18:0_20:0 + PI 18:0_22:0)

Ratio of UFA-PIs to SFA-PIs	(PI 14:0_18:1 + PI 14:0_18:2 + PI 15:1_16:0 + PI 16:0_17:1 + PI 16:0_17:2 + PI 16:0_18:1 + PI 16:0_18:2 + PI 16:0_18:3 + PI 16:0_20:3 + PI 16:0_20:4 + PI 16:0_22:1 + PI 16:1_18:0 + PI 16:1_18:1 + PI 16:1_18:2 + PI 17:0_18:1 + PI 17:1_18:1 + PI 17:1_18:2 + PI 18:0_18:1 + PI 18:0_18:2 + PI 18:0_18:3 + PI 18:0_20:3 + PI 18:0_20:4 + PI 18:1_18:1 + PI 18:1_18:2 + PI 18:1_18:3 + PI 18:1_20:0 + PI 18:1_20:1 + PI 18:1_20:2 + PI 18:1_20:3 + PI 18:1_20:4 + PI 18:1_20:5 + PI 18:1_22:0 + PI 18:1_22:1 + PI 18:1_22:2 + PI 18:1_22:3 + PI 18:1_22:4 + PI 18:1_22:5 + PI 18:1_22:6 + PI 18:2_18:3 + PI 18:2_20:0 + PI 18:2_20:1 + PI 18:2_20:4 + PI 18:2_20:5 + PI 18:2_22:0 + PI 18:2_22:1 + PI 18:2_22:6) / (PI 15:0_16:0 + PI 16:0_16:0 + PI 16:0_17:0 + PI 16:0_20:0 + PI 18:0_18:0 + PI 18:0_20:0 + PI 18:0_22:0)
Ratio of PI 18:0_20:4 to PIs	PI 18:0_20:4 / (PI 14:0_18:1 + PI 14:0_18:2 + PI 15:0_16:0 + PI 15:1_16:0 + PI 16:0_16:0 + PI 16:0_17:0 + PI 16:0_17:1 + PI 16:0_17:2 + PI 16:0_18:1 + PI 16:0_18:2 + PI 16:0_18:3 + PI 16:0_20:0 + PI 16:0_20:3 + PI 16:0_20:4 + PI 16:0_22:1 + PI 16:1_18:0 + PI 16:1_18:1 + PI 16:1_18:2 + PI 17:0_18:1 + PI 17:1_18:1 + PI 17:1_18:2 + PI 18:0_18:0 + PI 18:0_18:1 + PI 18:0_18:2 + PI 18:0_18:3 + PI 18:0_20:0 + PI 18:0_20:3 + PI 18:0_20:4 + PI 18:0_22:0 + PI 18:1_18:1 + PI 18:1_18:2 + PI 18:1_18:3 + PI 18:1_20:0 + PI 18:1_20:1 + PI 18:1_20:2 + PI 18:1_20:3 + PI 18:1_20:4 + PI 18:1_20:5 + PI 18:1_22:0 + PI 18:1_22:1 + PI 18:1_22:2 + PI 18:1_22:3 + PI 18:1_22:4 + PI 18:1_22:5 + PI 18:1_22:6 + PI 18:2_18:3 + PI 18:2_20:0 + PI 18:2_20:1 + PI 18:2_20:4 + PI 18:2_20:5 + PI 18:2_22:0 + PI 18:2_22:1 + PI 18:2_22:6)
Sum of MUFA-PIs	PI 14:0_18:1 + PI 15:1_16:0 + PI 16:0_17:1 + PI 16:0_18:1 + PI 16:0_22:1 + PI 16:1_18:0 + PI 17:0_18:1 + PI 18:0_18:1 + PI 18:1_20:0 + PI 18:1_22:0 + PI 16:1_18:1 + PI 17:1_18:1 + PI 18:1_18:1 + PI 18:1_20:1 + PI 18:1_22:1
Sum of PIs	PI 14:0_18:1 + PI 14:0_18:2 + PI 15:0_16:0 + PI 15:1_16:0 + PI 16:0_16:0 + PI 16:0_17:0 + PI 16:0_17:1 + PI 16:0_17:2 + PI 16:0_18:1 + PI 16:0_18:2 + PI 16:0_18:3 + PI 16:0_20:0 + PI 16:0_20:3 + PI 16:0_20:4 + PI 16:0_22:1 + PI 16:1_18:0 + PI 16:1_18:1 + PI 16:1_18:2 + PI 17:0_18:1 + PI 17:1_18:1 + PI 17:1_18:2 + PI 18:0_18:0 + PI 18:0_18:1 + PI 18:0_18:2 + PI 18:0_18:3 + PI 18:0_20:0 + PI 18:0_20:3 + PI 18:0_20:4 + PI 18:0_22:0 + PI 18:1_18:1 + PI 18:1_18:2 + PI 18:1_18:3 + PI 18:1_20:0 + PI 18:1_20:1 + PI 18:1_20:2 + PI 18:1_20:3 + PI 18:1_20:4 + PI 18:1_20:5 + PI 18:1_22:0 + PI 18:1_22:1 + PI 18:1_22:2 + PI 18:1_22:3 + PI 18:1_22:4 + PI 18:1_22:5 + PI 18:1_22:6 + PI 18:2_18:3 + PI 18:2_20:0 + PI 18:2_20:1 + PI 18:2_20:4 + PI 18:2_20:5 + PI 18:2_22:0 + PI 18:2_22:1 + PI 18:2_22:6
Sum of PUFA-PIs	PI 14:0_18:2 + PI 16:0_17:2 + PI 16:0_18:2 + PI 16:0_18:3 + PI 16:0_20:3 + PI 16:0_20:4 + PI 16:1_18:2 + PI 17:1_18:2 + PI 18:0_18:2 + PI 18:0_18:3 + PI 18:0_20:3 + PI 18:0_20:4 + PI 18:1_18:2 + PI 18:1_18:3 + PI 18:1_20:2 + PI 18:1_20:3 + PI 18:1_20:4 + PI 18:1_20:5 + PI 18:1_22:2 + PI 18:1_22:3 + PI 18:1_22:4 + PI 18:1_22:5 + PI 18:1_22:6 + PI 18:2_18:3 + PI 18:2_20:0 + PI 18:2_20:1 + PI 18:2_20:4 + PI 18:2_20:5 + PI 18:2_22:0 + PI 18:2_22:1 + PI 18:2_22:6
Sum of SFA-PIs	PI 15:0_16:0 + PI 16:0_16:0 + PI 16:0_17:0 + PI 16:0_20:0 + PI 18:0_18:0 + PI 18:0_20:0 + PI 18:0_22:0

Sum of UFA-PIs	PI 14:0_18:1 + PI 14:0_18:2 + PI 15:1_16:0 + PI 16:0_17:1 + PI 16:0_17:2 + PI 16:0_18:1 + PI 16:0_18:2 + PI 16:0_18:3 + PI 16:0_20:3 + PI 16:0_20:4 + PI 16:0_22:1 + PI 16:1_18:0 + PI 16:1_18:1 + PI 16:1_18:2 + PI 17:0_18:1 + PI 17:1_18:1 + PI 17:1_18:2 + PI 18:0_18:1 + PI 18:0_18:2 + PI 18:0_18:3 + PI 18:0_20:3 + PI 18:0_20:4 + PI 18:1_18:1 + PI 18:1_18:2 + PI 18:1_18:3 + PI 18:1_20:1 + PI 18:1_20:2 + PI 18:1_20:3 + PI 18:1_20:4 + PI 18:1_20:5 + PI 18:1_22:0 + PI 18:1_22:1 + PI 18:1_22:2 + PI 18:1_22:3 + PI 18:1_22:4 + PI 18:1_22:5 + PI 18:1_22:6 + PI 18:2_18:3 + PI 18:2_20:0 + PI 18:2_20:1 + PI 18:2_20:4 + PI 18:2_20:5 + PI 18:2_22:0 + PI 18:2_22:1 + PI 18:2_22:6
Ratio of PUFA-PSs to MUFA-PSs	(PS 36:3 + PS 36:4 + PS 36:5 + PS 38:4 + PS 38:5 + PS 38:6 + PS 38:7 + PS 40:4 + PS 40:5 + PS 40:6 + PS 40:7 + PS 40:8) / (PS 34:1 + PS 36:1)
Sum of MUFA-PSs	PS 34:1 + PS 36:1
Sum of PUFA-PSs	PS 36:3 + PS 36:4 + PS 36:5 + PS 38:4 + PS 38:5 + PS 38:6 + PS 38:7 + PS 40:4 + PS 40:5 + PS 40:6 + PS 40:7 + PS 40:8
Sum of UFA-PSs	PS 34:1 + PS 34:2 + PS 36:1 + PS 36:2 + PS 36:3 + PS 36:4 + PS 36:5 + PS 38:4 + PS 38:5 + PS 38:6 + PS 38:7 + PS 40:4 + PS 40:5 + PS 40:6 + PS 40:7 + PS 40:8
SPBP d18:1 to Cer d18:1/24:1 Ratio	SPBP d18:1 / Cer d18:1/24:1
SPHK activity (4)	SPBP d18:1 / SPB d18:1
SPHK activity (6)	SPBP d14:0 / SPB d14:0
SPHK activity (8)	SPBP d17:0 / SPB d17:0
Sum of SphoPs	SPBP d14:1 + SPBP d16:1 + SPBP d17:1 + SPBP d18:1
LPP3 activity (4)	SPB d18:1 / SPBP d18:1
Sum of Spha	SPB d14:0 + SPB d16:0 + SPB d17:0 + SPB d18:0
Ratio of OC-FA SMs to EC-FA SMs	(SM 33:1 + SM 35:1 + SM 41:1 + SM 41:2 + SM 43:1) / (SM 34:1 + SM 34:2 + SM 36:1 + SM 36:2 + SM 38:3 + SM 42:1 + SM 42:2 + SM 44:1 + SM 44:2)
Sum of EC-FA SMs	SM 34:1 + SM 34:2 + SM 36:1 + SM 36:2 + SM 38:3 + SM 42:1 + SM 42:2 + SM 44:1 + SM 44:2
Sum of LCFA-SMs	SM 33:1 + SM 35:1 + SM 34:1 + SM 34:2 + SM 36:1 + SM 36:2
Sum of OC-FA SMs	SM 33:1 + SM 35:1 + SM 41:1 + SM 41:2 + SM 43:1
Sum of SMs	SM 33:1 + SM 35:1 + SM 41:1 + SM 41:2 + SM 43:1 + SM 34:1 + SM 34:2 + SM 36:1 + SM 36:2 + SM 38:3 + SM 42:1 + SM 42:2 + SM 44:1 + SM 44:2
Sum of VLCFA-SMs	SM 43:1 + SM 42:1 + SM 42:2 + SM 44:1 + SM 44:2

SUPPLEMENTAL DATA 4

Analyte class	Number of molecules
Acylcarnitines	40
Alkaloids	2
Amine oxides	1
Amino acid-related	77
Amino acids	20
Bile acids	24
Biogenic amines	10
Carboxylic acids	8
Catechols	3
Ceramides	29
Cholesteryl esters	22
Cresols	2
Dicarboxylic acids	25
Diglycerides	41
Dihexosylceramides	9
Dihydroceramides	8
Fatty acids	39
Hexosylceramides	20
Hormones	5
Indoles and derivatives	17
Lysophosphatidic acids	8
Lysophosphatidylcholines	12
Lysophosphatidylethanolamines	43
Lysophosphatidylglycerols	10
Lysophosphatidylinositols	15
Lysophosphatidylserines	12
Monoglycerides	12
Nucleobase-related	14
Nucleobases	5
Nucleotides	2
Organic acids	16
Phenolic acids	22
Phenoxy compounds	2
Phosphatidic acids	41
Phosphatidylcholines	76
Phosphatidylethanolamines	95
Phosphatidylglycerols	64
Phosphatidylinositols	53
Phosphatidylserines	18

Polyamines	7
Pyridinecarboxylic acids	6
Sphinganine and sphingosine phosphates	8
Sphinganines and sphingosines	8
Sphingomyelins	14
Sugars	7
Tricarboxylic acids	3
Triglycerides	242
Trihexosylceramides	6
Vitamins and cofactors	8
